# A Systematic Review and Meta-Analysis of the Association Between Physical Capability, Social Support, Loneliness, Depression, Anxiety, and Life Satisfaction in Older Adults

**DOI:** 10.1093/geront/gnae128

**Published:** 2024-09-05

**Authors:** Santi Sulandari, Rachel O Coats, Amy Miller, Alexander Hodkinson, Judith Johnson

**Affiliations:** School of Psychology, University of Leeds, Leeds, UK; Faculty of Psychology, Universitas Muhammadiyah Surakarta, Surakarta City, Central Java, Indonesia; School of Psychology, University of Leeds, Leeds, UK; School of Psychology, University of Leeds, Leeds, UK; Division of Population Health, Health Services Research and Primary Care, Faculty of Biology, Medicine and Health, National Institute for Health and Care Research (NIHR) School for Primary Care Research, School of Health Sciences, Manchester Academic Health Science Centre, University of Manchester, Manchester, UK; Division of Population Health, Health Services Research and Primary Care, National Institute for Health Research Greater Manchester Patient Safety Research Collaboration, University of Manchester, Manchester, UK; School of Psychology, University of Leeds, Leeds, UK; Division of Nursing, Midwifery and Social Work, School of Health Sciences, Faculty of Biology, Medicine and Health, University of Manchester, UK

**Keywords:** Cross-cultural studies, Function/mobility, Successful aging, Well-being

## Abstract

**Background and Objectives:**

Physical capability, social support, loneliness, depression, and anxiety predict life satisfaction in older adults. Currently, no systematic review and meta-analysis have been conducted to investigate the strength of these associations globally. Therefore, this study quantified the strength of these associations.

**Research Design and Methods:**

A systematic literature search was conducted using MEDLINE, EMBASE, APA PsycINFO, Web of Science, and Scopus. We included observational studies assessing the association between physical capability, social support, loneliness, depression, and anxiety with life satisfaction in adults aged 65+.

**Results:**

In total, 10,552 articles were identified, of which 78 studies in 164,478 participants were included in the systematic review and 57 were included in the meta-analysis. Greater life satisfaction was significantly associated with greater physical capabilities (odds ratio [OR] = 2.64; 95% confidence interval [CI]: 2.01–3.45; *p* < .001, *k* = 35, *n* = 33,732), higher social support (OR = 3.27; 95% CI: 2.59–4.13, *k* = 20 studies, *n* = 13,228), reduced loneliness (OR = 3.30; 95% CI: 2.53–4.30, *k* = 11, *n* = 33,638), depression (OR = 4.76; 95% CI: 3.10–7.32, *k* = 24, *n* = 64,097), and anxiety (OR = 5.10; 95% CI: 2.21–11.78, *k* = 5, *n* = 43,368). The strength of associations did not vary between Western and Eastern countries, year of publication, or quality. Gender was a moderator: Loneliness was more strongly associated with life satisfaction in females. Age was also a moderator; the association between social support and life satisfaction weakened with increasing age.

**Discussion and Implications:**

Improving the physical capabilities of older individuals, fostering social support, and alleviating feelings of loneliness, depression, and anxiety may help build life satisfaction in older individuals, which policy-makers and healthcare professionals should prioritize when implementing strategies.

## Background

Globally, the proportion of individuals aged 65 or above is projected to rise from 10% in 2022 to 16% in 2050 (United Nations Department of Economic and Social Affairs, 2022). Healthcare expenses increase substantially during the later stages of life. An analysis conducted across Organization for Economic Cooperation and Development (OECD Better Life Index) countries and Brazil, Russia, India, China, and South Africa (BRIICS) countries projects that in 2060, 60% of healthcare expenses will be allocated to individuals aged 65 and above (de la Maisonneuve & Martins, 2014). Expenses associated with providing care for older individuals escalate as they grow older (Office for Budget Responsibility, 2016). Additionally, Jacobson et al. (2021) revealed that financial constraints resulting from rising healthcare costs lead to a higher likelihood of older Americans abstaining from seeking medical care.

Due to these projected aging-related pressures on health systems, there has been a growing interest in promoting and maintaining aging well. Life satisfaction (LS) could be a useful concept to focus on when investigating how we can better support older adults to “age well.” Life satisfaction is the cognitive-judgment component of subjective well-being (Diener et al., 1985). Hall (2014) defined it as “an endorsement of, or positive attitude toward one’s life overall” (p. 3599). Low life satisfaction was associated with a higher risk of suicide and a desire to alter one’s circumstances (Diener, 2012). Additionally, life satisfaction in older people is related to physical and mental health (Choudhary, 2015) and quality of life (Vinsalia & Handajani, 2021). Finally, although there are differences between individuals, previous studies have found people’s life satisfaction declines as they grow older (Baird et al., 2010; Park et al., 2019).

Studies have identified many factors that contribute to older people’s life satisfaction. Better physical and mental health are associated with higher life satisfaction (Choudhary, 2015). For example, a study by Puvill et al. (2019) in Europe showed that limitations in the activities of daily living (ADLs), instrumental activities of daily living, mental health, depression, and social resources were associated with older adults’ life satisfaction. Similarly, a study on Korean older adults (Shin & Sok, 2012) revealed that perceived health conditions, self-esteem, and depression, as well as demographic data like age and monthly allowance, were determinants of life satisfaction of older persons. In Europe, Fagerström et al. (2007) emphasized that lacking social contact and sensing health issues are a hindrance, and having poor self-esteem could lead to lower life satisfaction. Furthermore, in Vietnam, a study showed that factors contributing to life satisfaction were health, income, and social relationships of older people (Trinh et al., 2022).

Of the wide range of factors that have been associated with life satisfaction in older people, some have been identified as consistent determinants. These include the physical health indicator of physical capability (Elmstahl et al., 2020; Puvill et al., 2019; Pynnönen et al., 2021). They also include social support (Fagerström et al., 2007; Park & Sok, 2020; Tian & Wang, 2021), loneliness (Fukuzawa & Sugawara, 2022; Rochelle, 2022), and the mental health variables of depression and anxiety (Dong et al., 2020; Hannaford et al., 2018; Park & Sok, 2020). Physical capability can be defined as the ability to carry out the physical chores of daily living (Cooper et al., 2011), for example, dressing, eating, toileting, grooming, and mobility. Social support is the actual or perceived availability of social resources that may be used to provide comfort or help (Lepore, 2012). It is also a particularly relevant concept for older adults, due to concerns that social support reduces over the lifespan, potentially resulting in loneliness. Loneliness is defined as feelings of isolation and longing for companionship that follows a sense that one’s social requirements are not being supplied by the amount or quality of one’s social contacts (Hawkley & Cacioppo, 2010; Pinquart & Sorensen, 2001). Depression can be understood as a multifactorial disorder characterized by distinct behavioral or motor symptoms that render the individual less likely to receive reinforcement from their environment, which leads to challenges with daily functioning (Bernard, 2018). It can involve feelings of sadness, hopelessness, and worthlessness, coupled with a lack of interest or pleasure. Anxiety is often measured alongside depression and captures an adaptive reaction to a threat, which can become maladaptive under certain situations (House & Stark, 2002), for example, experiencing immense tension and worry, which interferes with daily life.

Studies examining the association of physical capability, social support, loneliness, depression, and anxiety, with life satisfaction in older people have been widely conducted in several countries, for example, China (Jin et al., 2018; Qin et al., 2020; Tian & Wang, 2021), Vietnam (Trinh et al., 2022), Turkey (Tümer et al., 2022), England (Smith et al., 2004), Finland (Pynnönen et al., 2021), Iran (Pahlevan Sharif et al., 2021), Sweden (Dong et al., 2020; Elmstahl et al., 2020), Scotland (Hannaford et al., 2018), The Netherlands (Puvill et al., 2016), Poland and Germany (Bidzan-Bluma et al., 2020), and Korea (Park et al., 2012; Park & Sok, 2020).

However, despite this relatively extensive research, systematic reviews on the association between life satisfaction in older people and its factors are lacking. One previous systematic review of older people’s life satisfaction was conducted by Khodabakhsh (2021). This found that 19 main factors were associated with older adults’ life satisfaction including depression, physical capability, and social support. However, this review only included studies from Asia, so the generalizability of these findings to other continents is unclear. They also did not conduct meta-analyses, so the strength of the associations was not examined. This is important: Although a range of variables appears to be associated with life satisfaction, it is unclear which variables are most closely associated with this outcome variable and which interventions to enhance aging well should be prioritized. A meta-analytic review of key factors could help to identify priority areas.

There is also a need for systematic assessment of the role of potential influencing variables on the associations between these variables and life satisfaction, such as country of origin (Western vs Eastern countries). This is important, as Western countries are predominantly individualistic in their culture and Eastern countries are predominantly collectivist, which has been observed to influence aging-related well-being. For example, in their systematic review and meta-analysis of the association between subjective age and the combined measures of cognition, subjective well-being, and depression, Debreczeni and Bailey (2021) conducted subgroup analysis and found that the overall effect of the association was stronger in collectivist cultures than individualist cultures. Cultural variations between Eastern and Western nations can also lead to differing findings on older people’s life satisfaction (OECD Better Life Index, 2020; Ortiz-Ospina & Roser, 2024; Somaraju et al., 2021) and may potentially have an effect on its association with related factors. Additionally, the quality and effectiveness of its social policy and healthcare systems may play an important role in the overall level of satisfaction among older people in each country (Barrington-Leigh, 2021; Livani & Graham, 2019). Therefore, there is a need to examine this moderator. Furthermore, it is also important to investigate the role of publication year, age, gender, and quality of the studies in meta-analyses to understand potential heterogeneity in outcomes (Daoud et al., 2018; Hallford et al., 2016; Sá et al., 2019; Wiktorowicz et al., 2022; Zamani et al., 2019).

To address these gaps, we conducted the first systematic review and meta-analysis examining the strength of associations of physical capability, social support (perceived social support and loneliness), and mental health (depression and anxiety), with life satisfaction from studies conducted across the globe. We also compared findings between Eastern and Western countries, to understand if associations were internationally generalizable or varied between cultures.

## Method

**Figure 1.**
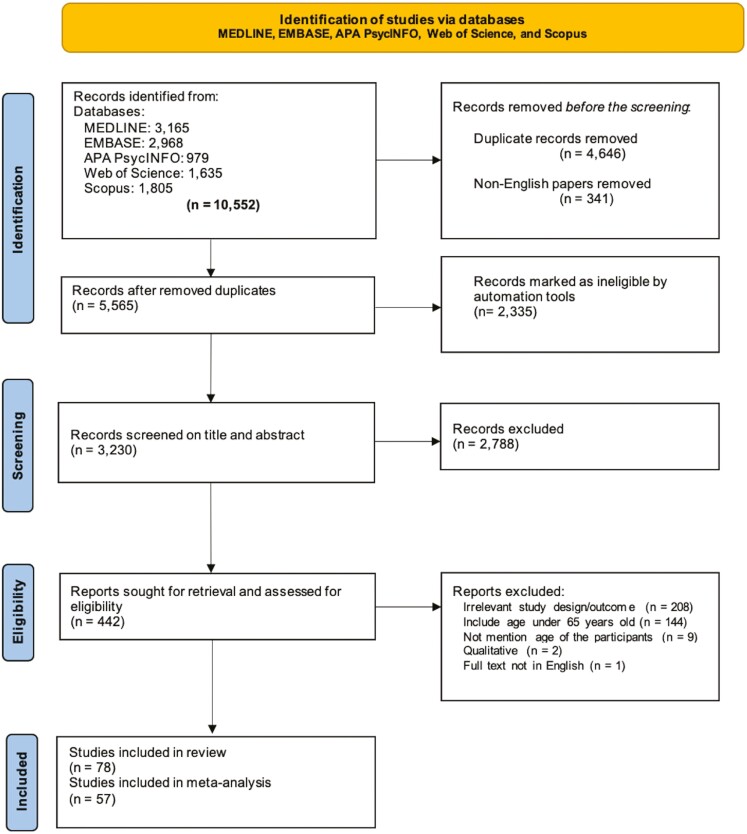
Study selection flow diagram (PRISMA 2020) showing records identified and screened for eligible studies. PRISMA = Preferred Reporting Items for Systematic Reviews and Meta-Analysis.

We reported our findings in accordance with the Preferred Reporting Items for Systematic Reviews and Meta-Analysis (PRISMA) guidelines (Liberati et al., 2009; Page et al., 2021), see Figure 1 for PRISMA flow diagram and Supplementary Table 1 for PRISMA checklist. This study was registered on PROSPERO with registration number CRD42022337584.

### Search Strategy and Study Eligibility

Figure 1 shows the flow diagram for the study selection process. Electronic literature searches were performed in five scientific databases: MEDLINE, EMBASE, APA PsycINFO, Web of Science, and Scopus from inception to July 2, 2022, updated to April 01, 2024. The searches included combinations of subject-heading terms and text words: “depression,” “anxiety,” “physical capability,” “social support,” “loneliness,” “life satisfaction,” and “older adult.” This search strategy was agreed upon with the information specialist team at Leeds Institute of Health Sciences at the University of Leeds, UK. The detailed search strategies are presented in Supplementary Table 2.

### Inclusion and Exclusion Criteria

The studies were included if they met the inclusion criteria: Quantitative studies (observational cohort/cross-sectional studies), published in the English language, peer-reviewed, including older adults aged 65 years and above, provided association data between physical capability, social support, loneliness, depression, anxiety, and life satisfaction; for further information, see Supplementary B. The decision to limit this review to English language studies was due to the fact that resources available for translation and linguistic competence were limited, making it impossible to evaluate research in languages other than English. Furthermore, concentrating on English-language publications helped to verify that data extraction and interpretation were consistent throughout the studies examined. The category of physical capability included ADL, frailty, and physical capability/function. Social support in this present study focused on perceived overall social support. We excluded studies that investigated general health, senses, and chronic disease that had no relation to physical capability. Frailty was included where it was conceptualized as being related to daily activities, but excluded where it was measured according to body weight. We excluded studies where social support was measured only in relation to one group of people (e.g., family OR friends) or where it was measured only according to the number of people listed in a participant’s social network. We excluded studies that only included participants within a particular range of the life satisfaction outcome measure, for example, samples with low life satisfaction.

Initially, 10,552 records were identified from the databases (Figure 1). After removing duplicates (*n* = 4,646) and non-English papers (*n* = 341), there were 5,565 papers. This study used a three-stage selection process to include papers in the review. After conducting manual screening on 20% of the articles using our inclusion criteria (*n* = 1,113), Rayyan was used for automatic screening, see Valizadeh et al. (2022) for more details, and articles that were automatically rated as one and a half stars or less were excluded (*n* = 2,335). In the second round, 10% of titles/abstracts (*n* = 324) were independently double screened by both S. Sulandari and A. Miller to ensure consistency. Inter-rater agreement with κ statistic (κ = 0.80) was applied and any disagreement was resolved by consensus. Screening of the remaining titles/abstracts was carried out by S. Sulandari. Manual title and abstract screening led to a further 2,788 articles being excluded. In the third round, 442 full-text articles were retrieved and assessed for eligibility. Ten percent of the eligible full-text articles were independently double screened by S. Sulandari and A. Miller, the Kappa value was 1.00 (100% agreement). S. Sulandari continued the full-text screening. Of 442 full-text articles screened, 364 were excluded and 78 observational studies of 164,478 older adults were included in the systematic review (the citation for each study is provided in Supplementary C.1).

### Data Extraction and Quality Assessment

A data extraction form was devised in Excel and piloted in five randomly selected studies. Data extraction covered information relating to: author and year of publication, country, setting, outcome measure, relevant exposure measure (mental health/physical capability/social support), study design, sample age, mean age, sample size, gender/sex, summary of the availability of the relevant exposures, relevant key finding(s), summary of the quality assessment category (see Supplementary Tables 3 and 4). All data extractions were undertaken by two reviewers (S. Sulandari and A. Miller). The second reviewer (A. Miller) independently extracted 20% of the data from papers and this was then cross-checked by S. Sulandari to ensure consistency. Any divergence of opinions was ultimately resolved through the attainment of a consensus.

Articles were assessed for quality using the National Institutes of Health quality assessment tool for observational cohorts and cross-sectional studies. This tool consists of 14 items and examines the research question, study population, groups recruited from the same population and uniform eligibility criteria, sample size justification, exposure assessed prior to outcome measurement, sufficient timeframe to see an effect, different levels of the exposure of interest, exposure measures and assessment, repeated exposure assessment, outcome measures, blinding of outcome assessors, follow-up rate, and statistical analyses. The response for each item was categorized into: “Yes,” “No,” “NA” (not applicable), and “NR” (not reported). In the final rating, studies were categorized as “good,” “fair,” or “poor.” This overall rating of the study’s quality was based on the evaluation of each individual feature of the study design.

### Data Analysis

The outcome variable of interest was life satisfaction, with the association with physical capability, social support, loneliness, depression, and anxiety expressed as an odds ratio (OR). In this study, we compared the likelihood of an event occurring between different groups or conditions, for example, whether people with better physical capabilities were significantly more satisfied compared with those who had poorer physical capability. The following variables were reported as binary variables: physical capability (poorer physical capability vs better physical capability), social support (less social support vs better social support), loneliness (lonely vs less lonely), depression (depressed vs. less depressed), anxiety (anxious vs. less anxious), and life satisfaction (less satisfied vs satisfied). These variables were then assessed as ORs, as ORs are preferred when quantifying the relationship between two binary outcomes (Bland & Altman, 2000; Norton et al., 2018). This approach has been used in previous robust meta-analyses assessing the association between two variables (Hodkinson et al., 2022; Vila, 2021). When the raw metadata were not available, descriptive and test statistics indicating the association of the variable of interest were extracted instead and transformed into the OR with the corresponding standard error. When studies included more than one indicator for the examined variable, effect sizes were averaged avoiding double counting on the control (Donker et al., 2014; Holzberger et al., 2020; Malti & Krettenauer, 2013) using the Comprehensive Meta-Analysis software (Borenstein et al., 2022), resulting in a combined measure of effect sizes. But, for studies that had more than one sample group such as female and male groups, then both data sets were included in the analysis separately.

Comprehensive Meta-Analysis Software was used to perform the meta-analysis. Due to the variability in study design, target population, and variable measurements employed, a sensitivity analysis was conducted to assess the impact of removing individual studies on the overall pooled result in the pooled analysis. This methodology enables the identification of specific elements within an individual study that could potentially introduce bias to the overall aggregated outcome. Additionally, a cumulative analysis was also applied to assess the impact of including each study individually on the overall pooled result. A DerSimonian and Laird random-effects model was applied in this meta-analysis due to high levels of heterogeneity (DerSimonian & Laird, 1986). The assessment of the heterogeneity of effect sizes was conducted utilizing the *Q* statistic and the *I*^2^ statistic. *I*^2^ was interpreted as 0%–29%, 30%–59%, 60%–89%, and >89%, indicating low, moderate, substantial, and high heterogeneity, respectively (Higgins & Thompson, 2002). Additionally, prediction intervals were also calculated in meta-analyses involving more than 10 studies to express the amount of heterogeneity (Riley et al., 2011). Moderator analysis was also performed using subgroup analysis for Western studies compared with Eastern studies and studies of fair quality compared with studies of good quality. Univariate meta-regression was also used to assess the effect of the publication year, mean age, and proportion of female participants. For mean age and proportion of female participants moderators, only studies that provided this data could be included in these moderator analyses. The Western and Eastern countries were categorized in line with previous studies using the World Bank classification system (Daoud et al., 2018; Zhao et al., 2021). Additionally, United Nations specifications were also referred as to whether they are considered Eastern (e.g., Asia-Pacific) or Western countries (e.g., Eastern European States, Western European, and other States; United Nations Department for General Assembly and Conference Management, 2023). However, Türkiye was in both groups “Asia-Pacific” and “Western European and other States,” so we classified it as an Eastern country as the most populated areas were in Asia.

Publication bias was assessed in funnel plot. Egger’s test was also employed to assess for publication bias (Sterne et al., 2001). Additionally, trim-and-fill method, classic fail-safe N, and Begg and Mazumdar rank correlation were also examined.

## Results

The mean age of participants was 77.46 years old (data on mean age were missing for 23 studies). The countries where the studies were conducted included: Australia (1), Belgium (1), Brazil (1), Canada (4), China (11), Finland (1), Germany (3), Ireland (2), Japan (3), Korea (12), Norway (1), Portugal (1), Russia (1), Slovenia (1), Spain (6), Sweden (5), Taiwan (1), Turkey (6), United Kingdom (5), United States (11), Switzerland (1). Sixty-five studies included participants from the community and five studies focused on individuals residing in care homes (such as family healthcare centers, long-term care facilities, or old-age homes), whereas three other studies’ settings were not specified. Additionally, five studies included participants from a combination of settings, including the community/private dwellings, and care homes. There was a total of 65 cross-sectional investigations, with 8 of them incorporating baseline data from existing longitudinal research. Additionally, there were 10 longitudinal studies, 1 prospective cohort study, 1 retrospective cohort study, and 1 experimental study. Fifty-seven studies (where the relevant data were available) were included in the meta-analysis. Twenty-one studies were not included in the meta-analysis due to not providing amenable data for meta-analysis (see Supplementary C.2). There were 35 studies on physical capability, 20 on social support, 11 on loneliness, 24 studies on depression, and 5 on anxiety. The studies were dominated by female participants (55% of the total participants of included studies), counted from 54 studies included in the meta-analysis. Information about the gender of participants for the other three studies was not available. Forty-six studies had samples that were predominantly female (more than 50%). A descriptive summary with data tables can be found in Supplementary Tables 3 and 4, which were produced to summarize the literature.

### Quality Assessment

Twenty percent (*n* = 16) of the eligible articles were independently double rated for quality assessment by S. Sulandari and A. Miller to ensure consistency, and any disagreements were resolved through the process of reaching a consensus. An agreement was achieved and S. Sulandari continued to extract the remaining eligible studies. In the quality assessment check, 43 studies were categorized as “good” and 35 studies were categorized as “fair” (Supplementary Table 5).

### Meta-Analysis of the Association of Physical Capability and Life Satisfaction

**Figure 2.**
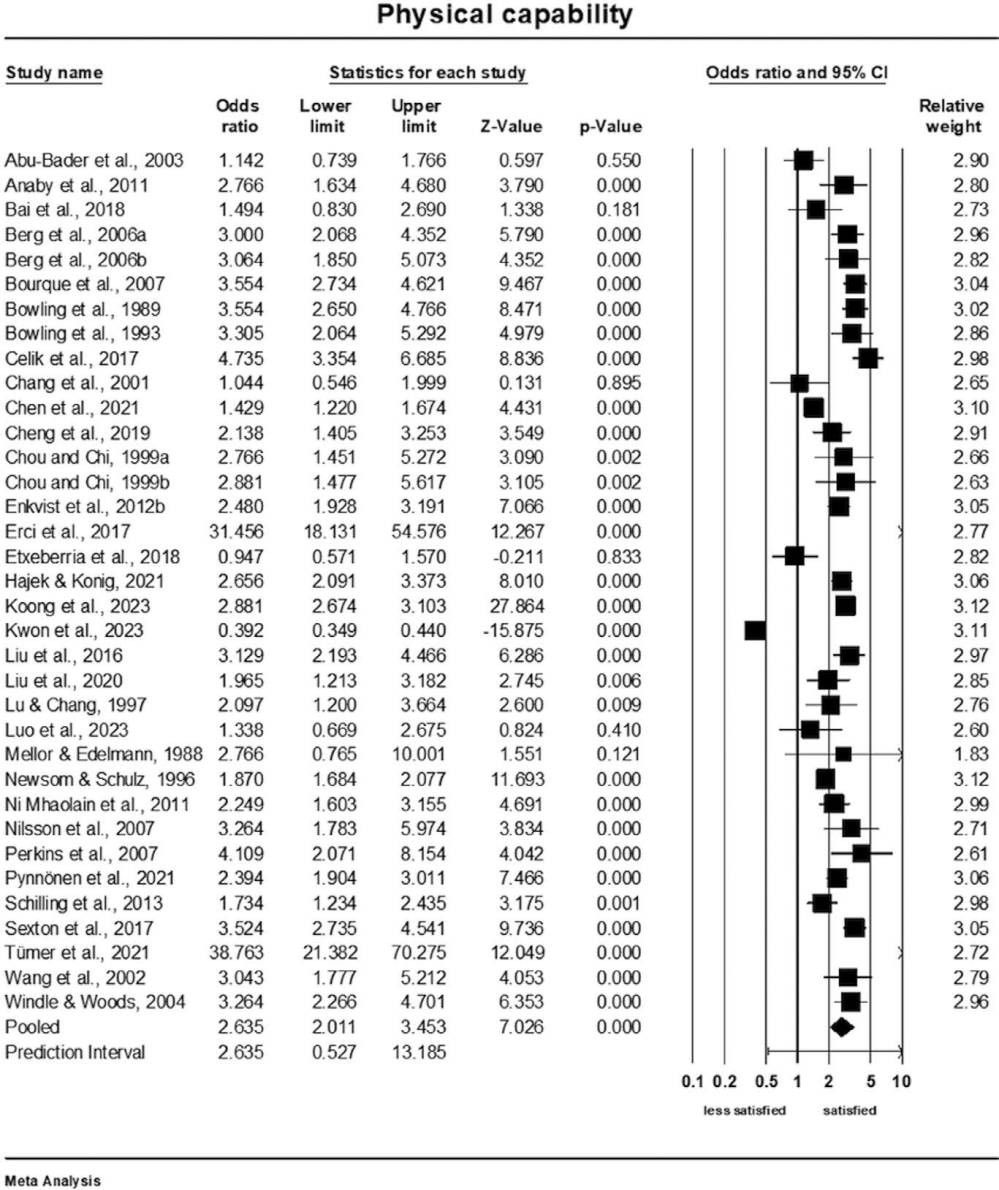
Forest plot of the association of physical capability and life satisfaction.

Older adults who had better physical capability related to daily activities were nearly three times more satisfied than those with physical limitations (OR = 2.64; 95% confidence interval [CI]: 2.01–3.45, *Q*-value = 1,212.28; *p* < .001, *I*^2^ = 97%, *k* = 35 studies, *n* = 33,732), see Figure 2. The prediction interval was 0.53–13.19, which means that the true effect size fell in this interval. The prediction interval provided context for understanding the mean effect size due to high levels of heterogeneity. A sensitivity analysis was conducted to assess the impact of excluding each study individually on the overall pooled result. The analysis revealed that none of the individual studies had a significant influence on the pooled outcome (Supplementary Figure 1). The cumulative analysis also suggested that the results remained unchanged as each study was included in the pool (Supplementary Figure 2).

### Meta-Analysis of the Association of Social Support and Life Satisfaction

**Figure 3.**
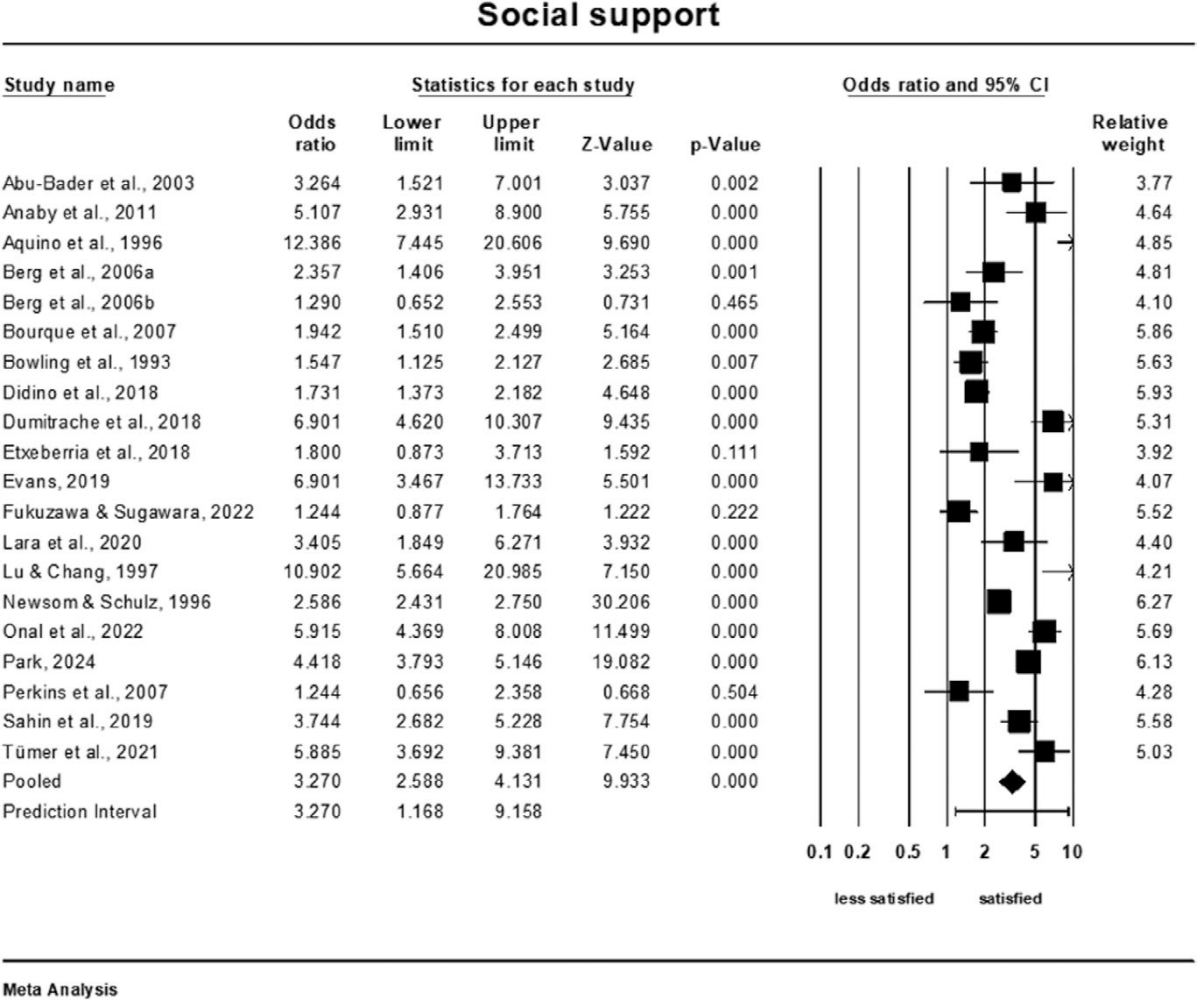
Forest plot of the association of social support and life satisfaction.

Older adults who perceived themselves as having more social support were more than three times more satisfied than those who perceived themselves as having less social support (OR = 3.27; 95% CI: 2.59–4.13, *Q*-value = 228.88; *p* < .001, *I*^2^= 92%, *k* = 20, *n* = 13,228), see Figure 3. The prediction interval was 1.17–9.16. A sensitivity analysis was conducted to assess the impact of excluding each study individually on the overall pooled result. The analysis revealed that none of the individual studies had a significant influence on the pooled outcome (Supplementary Figure 3). The cumulative analysis also suggested that the results remained unchanged as each study was included in the pool (Supplementary Figure 4).

### Meta-Analysis of the Association of Loneliness and Life Satisfaction

**Figure 4.**
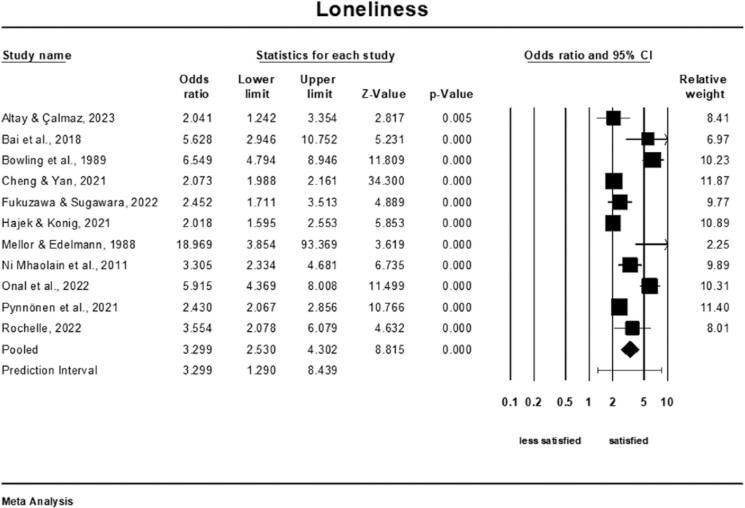
Forest plot of the association of loneliness and life satisfaction.

Older adults who reported lower levels of loneliness were almost three and a half times more satisfied than those with higher levels of loneliness (OR = 3.30; 95% CI: 2.53–4.30, *Q*-value = 121.60; *p* < .001, *I*^2^ = 92%, *k* = 11, *n* = 33,638), see Figure 4. The prediction interval was 1.29–8.44. A sensitivity analysis was conducted to assess the impact of excluding each study individually on the overall pooled result. The analysis revealed that none of the individual studies had a significant influence on the pooled outcome (Supplementary Figure 5). The cumulative analysis also suggested that the results remained unchanged as each study was included in the pool (Supplementary Figure 6).

### Meta-Analysis of the Association of Depression and Life Satisfaction

**Figure 5.**
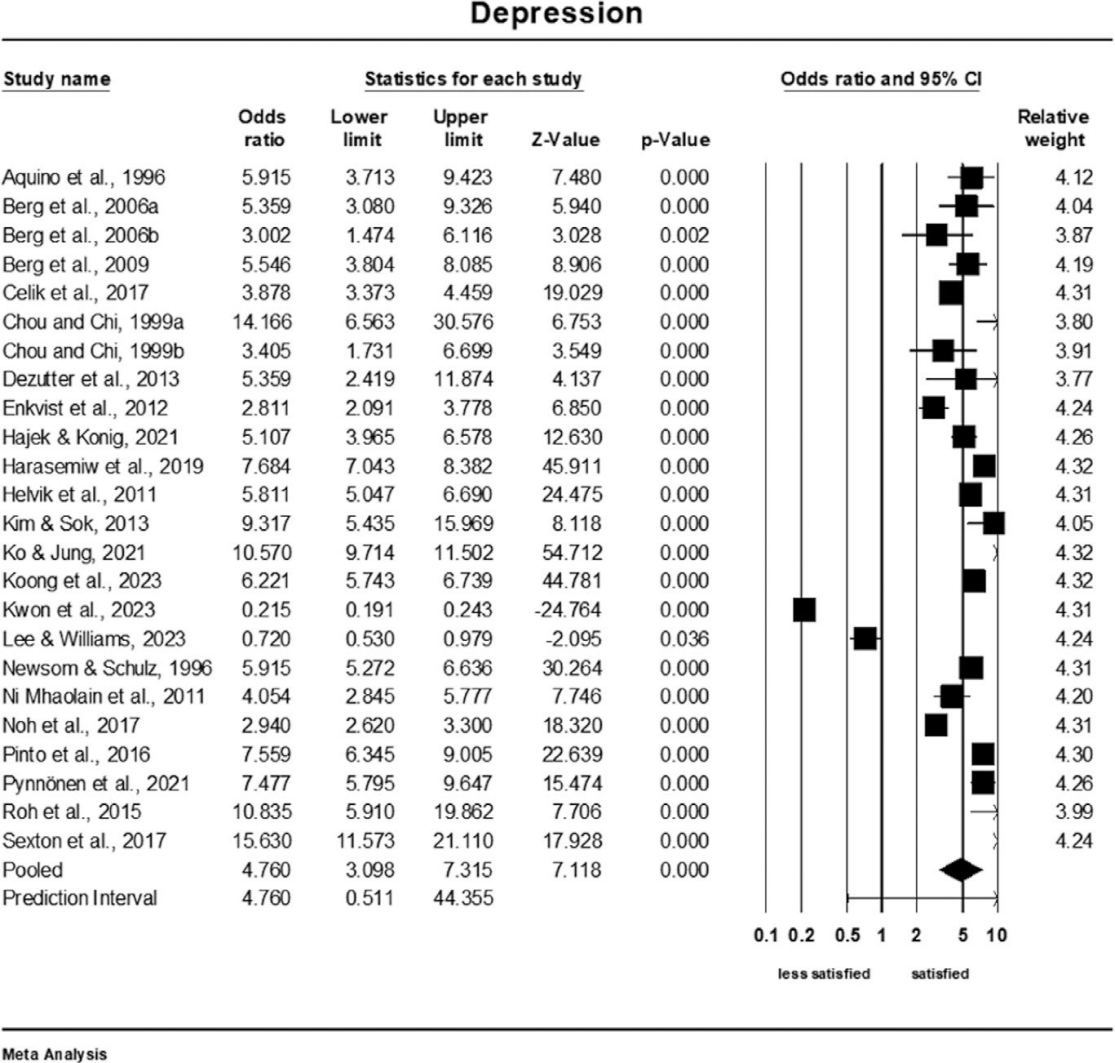
Forest plot of the association of depression and life satisfaction.

Older adults who were less depressed were nearly five times more satisfied than those who were more depressed (OR = 4.76; 95% CI: 3.10–7.32, *Q*-value = 3,372.64; *p* < .001, *I*^2^ = 99%, *k* = 24, *n* = 64,097), see Figure 5. The prediction interval was 0.51–44.36. A sensitivity analysis was conducted to assess the impact of excluding each study individually on the overall pooled result. The analysis revealed that none of the individual studies had a significant influence on the pooled outcome (Supplementary Figure 7). The cumulative analysis also suggested that the results remained unchanged as each study was included in the pool (Supplementary Figure 8).

### Meta-Analysis of the Association of Anxiety and Life Satisfaction

**Figure 6.**
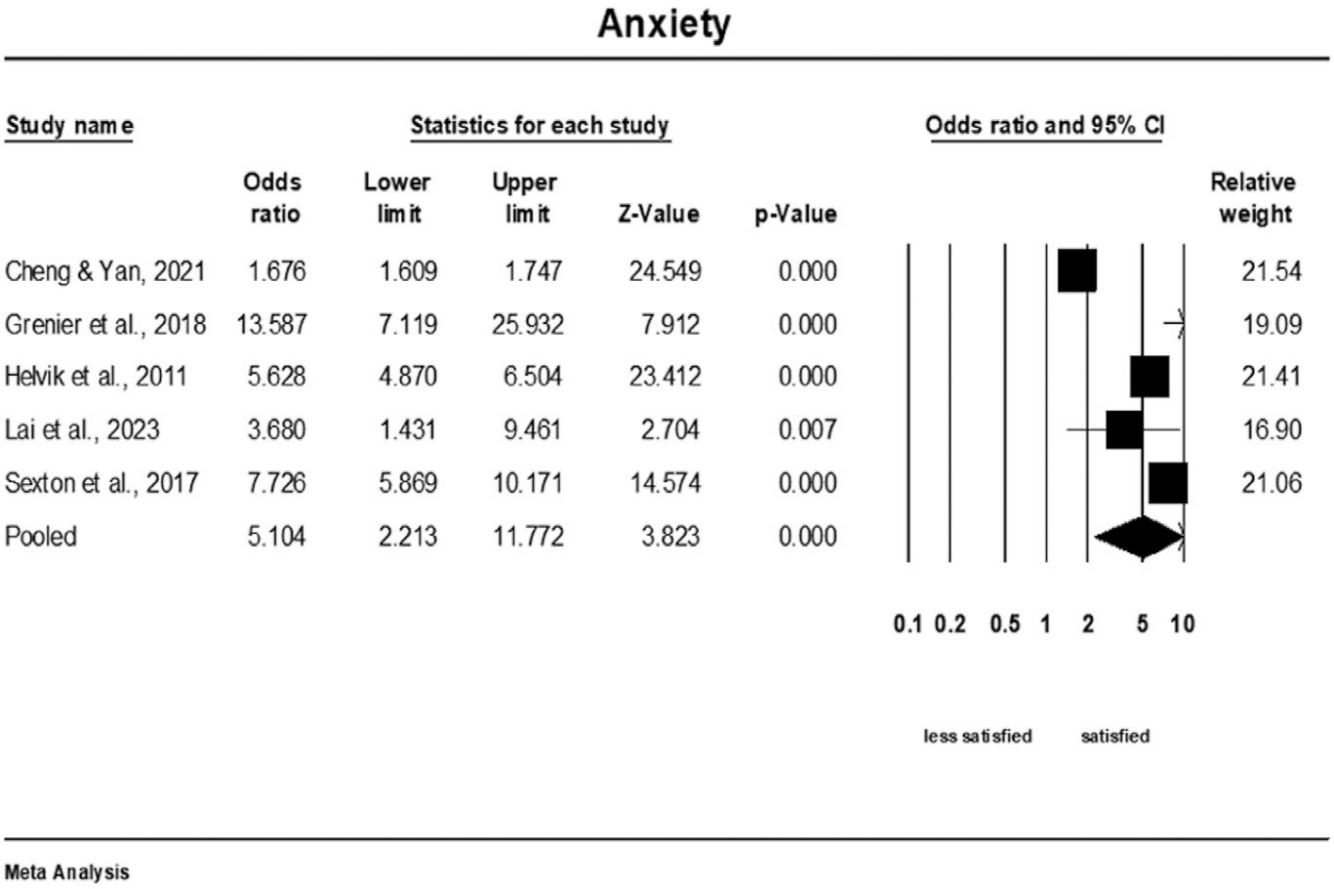
Forest plot of the association of anxiety and life satisfaction.

Older adults who had lower levels of anxiety were nearly five and a half times more satisfied than those who had higher levels of anxiety (OR = 5.10; 95% CI: 2.21–11.78, *Q*-value = 389.32; *p* < .001, *I*^2^ = 99%, *k* = 5, *n* = 43,368), see Figure 6. Prediction intervals were not calculated as fewer than 10 studies were included in this meta-analysis. Sensitivity analysis excluding each study individually revealed that none of the individual studies had a significant influence on the pooled outcome (Supplementary Figure 9). The cumulative analysis also suggested that the results remained unchanged as each study was included in the pool (Supplementary Figure 10).

The studies used various scales of measurement; therefore, the data were standardized through the use of standardized mean difference (SMD). The SMD pooled estimates have been calculated and results pooled using the SMD are provided in Supplementary Table 6 and Supplementary Figures 11–15. No changes in significance were found when pooling using SMD.

### Moderator Analysis

Moderator analysis was conducted to assess whether country group (Western vs Eastern), year of publication, mean age, quality assessment, and proportion of female participants moderated the association between physical capability, social support, loneliness, depression, and life satisfaction. Due to the limited number of studies of anxiety in relation to life satisfaction, moderator analysis was not conducted for this association.

Subgroup analysis was performed to examine whether country group (Western vs Eastern) moderated the association between physical capabilities, social support, loneliness, depression, and life satisfaction. Table 1 indicated that no statistically significant moderations were identified, see Supplementary Figures 16–19) for the subgroup analyses Forest plots. The association between physical capability and life satisfaction and the association between social support and life satisfaction in Eastern countries was stronger than in Western countries but these differences were not statistically significant. Meanwhile, the association between loneliness and life satisfaction and the association between depression and life satisfaction were stronger in Western countries than in Eastern countries, but these differences were not statistically significant.

**Table 1.** Subgroup Analyses

Variable	*k*	OR	95% CI	*Q*	*I* ^2^ (%)	τ^2^	*p* Values
*Association between physical capability and life satisfaction*							
Country group (overall)		2.50	2.16–2.90	0.32	97	0.61	.57
Eastern	14	2.96	1.62–5.41				
Western	21	2.47	2.12–2.88				
Quality assessment (overall)		2.61	2.22–3.07	0.01	97	0.61	.93
Fair	17	2.68	1.43–5.02				
Good	18	2.61	2.20–3.08				
*Association between social support and life satisfaction*							
Country group (overall)		3.18	2.52–4.01	2.21	92	0.23	.14
Eastern	6	4.31	2.71–6.86				
Western	14	2.87	2.20–3.76				
Quality assessment (overall)		2.90	2.26–3.70	3.16	92	0.23	.08
Fair	10	4.41	2.61–7.45				
Good	10	2.57	1.95–3.40				
*Association between loneliness and life satisfaction*							
Country group (overall)		3.37	2.45–4.62	0.102	92	0.39	.75
Eastern	6	3.21	2.07–4.95				
Western	5	3.56	2.24–5.65				
Quality assessment (overall)		3.15	2.40–4.14	0.85	92	0.39	.36
Fair	6	3.98	2.26–7.03				
Good	5	2.94	2.15–4.01				
*Association between depression and life satisfaction*							
Country group(overall)		5.98	5.06–7.07	1.64	99	1.11	.20
Eastern	9	3.30	1.31–8.33				
Western	15	6.10	5.15–7.23				
Quality assessment (overall)		5.76	4.64–7.15	1.49	99	1.11	.22
Fair	6	2.54	0.67–9.62				
Good	18	5.89	4.73–7.33				

*Notes*: CI = confidence interval; OR = odds ratio.

Another subgroup analysis was also performed examining whether quality assessment group (Fair vs Good) moderated the association between physical capability, social support, loneliness, depression, and life satisfaction. The associations between physical capability and life satisfaction in “Fair” and “Good” quality studies were similar: this moderator was not statistically significant. Even though the overall effect sizes were stronger in the “Fair” group than the “Good” group for social support and loneliness in relation to life satisfaction, and stronger in the “Good” group than the “Fair” group for depression in relation to life satisfaction, these were not statistically significantly different in this subgroup analyses, see Table 1. Therefore, none of the effect sizes of the association between physical capability, social support, loneliness, depression, and life satisfaction were moderated by study quality, see Supplementary Figures 20–23 for the subgroup analyses Forest plots.

Meta-regression was conducted to assess whether some of the variation in effect size in each predictor could be explained by the year of publication, mean age, and proportion of female participants, see Table 2. In a regression model that included the intercept and year of publication as the predictors, year of publication did not influence the associations between physical capability and life satisfaction, social support and life satisfaction, loneliness and life satisfaction, or depression and life satisfaction. In a regression model that included the intercept and mean age as the predictors, mean age did not influence the associations between physical capability and life satisfaction, loneliness and life satisfaction, or depression and life satisfaction. Greater age of participants was significantly associated with a weaker association between social support and life satisfaction (Supplementary Figure 24); however, this subset of studies was highly heterogeneous, with a zero R-square analog, which suggests that they do not explain a large portion of the variability between the effect sizes. A high level of heterogeneity probably occurred because there were still other variables that moderated the association. Additionally, in a regression model that included the intercept and female proportion as the predictors, the female proportion did not influence the associations between physical capabilities and life satisfaction, social support and life satisfaction, or depression and life satisfaction. However, studies that included more female participants were significantly associated with a stronger association between loneliness and life satisfaction (Supplementary Figure 25). This subset of studies was highly heterogeneous, with gender as a moderator explaining 20% of the variance between the effect sizes.

**Table 2.** Meta-Regression of Associations Between Variables

Variable	*k*	β	*SE*	CI	*Q*	*I* ^2^ (%)	*p* Value
*Association between physical capability and life satisfaction*							
Year of publication	35	−0.002	0.02	−0.03 to 0.03	0.01	97	.91
Mean age	26	−0.003	0.01	−0.02 to 0.03	0.07	84	.80
Female proportion	33	−0.01	0.01	−0.02 to 0.001	0.91	90	.34
*Association between social support and life satisfaction*							
Year of publication	20	−0.003	0.01	−0.02 to 0.03	0.04	91	.84
Mean age	17	−0.07	0.01	−0.13 to −0.02	6.99	93	.008**
Female proportion	20	−0.02	0.01	−0.04 to 0.003	2.66	92	.10
*Association between loneliness and life satisfaction*							
Year of publication	8	−0.05	0.03	−0.10 to 0.004	3.21	85	.07
Mean age	8	−0.02	0.04	−0.09 to 0.05	0.19	78	.67
Female proportion	11	−0.02	0.01	0.004 to −0.04	5.87	90	.02*
*Association between depression and life satisfaction*							
Year of publication	24	−0.03	0.03	−0.08 to 0.03	1.04	99	.31
Mean age	18	−0.03	0.03	−0.09 to 0.02	1.40	96	.24
Female proportion	22	0.02	0.01	−0.0008 to 0.04	3.53	97	.06

*p < .05.

**p < .01.

### Publication Bias

For detailed results regarding publication bias, see Supplementary K; further description and Supplementary Figures 26–35 (for funnel plots and Tweedie’s “fill and trim”) included. Overall, there was no evidence to indicate publication bias significantly influenced any of the analyses.

A funnel plot with a symmetrically distributed combined effect size indicated the absence of publication bias. The funnel plots in this present study indicated predominantly symmetrical distributions for the analyses of physical capability, social support, and anxiety in relation to life satisfaction, but a less symmetrical distribution for the studies of loneliness and depression with life satisfaction. Duval and Tweedie’s “fill and trim” method suggested that the values were not significantly different from the original overall random-effects OR for physical capability (2.64; 95% CI: 2.01–3.46), social support (3.27; 95% CI: 2.59–4.13), anxiety (5.10; 95% CI: 2.21–11.78), or loneliness (2.90; 95% CI: 2.25–3.74). For depression, the values (OR = 3.46, 95% CI: 2.35–5.09) were lower than the original overall random-effects OR of 4.76 (95% CI: 3.10–7.32) indicating possible publication bias.

The Eggers tests suggested that there was no evidence of publication bias for physical capability (2.25; 95% CI: 0.99–5.49, *t* = 1.42, *df* = 33, *p* = .08), social support (1.12; 95% CI: −1.17 to 3.41, *t *=* *1.03, *df* = 18, *p* = .16), depression (−1.32; 95% CI: −10.21 to 7.57, *t* =* *0.31, *df* = 22, *p* = .38), and anxiety (9.05; 95% CI: −4.51 to 22.61, *t *= 2.12, *df* = 2, *p* = .06), but there was possible publication bias in loneliness (3.03; 95% CI: 0.85–5.21, *t = *3.14, *df* = 9, *p* = .006). Begg and Mazumdar rank correlation tests suggested there was no publication bias for any of the analyses. Kendall’s tau *b* was −0.12 (*p* = .15) for physical capability; 0.05 (*p* = .39) for social support; 0.25 (*p* = .14) for loneliness; 0.09 (*p* = .19) for depression; and −0.10 (*p* = .81) for anxiety.

## Discussion

### Principal Findings

Our findings suggested that individuals who were more physically capable, received more social support, were less lonely, less depressed, and less anxious were more likely to be satisfied with their lives. The associations between physical capability, social support, loneliness, depression, anxiety, and life satisfaction in older adults did not differ by cultural groups (Western vs Eastern), quality of assessment, or year of publication. However, the association between loneliness and life satisfaction was moderated by gender, such that the association is stronger in studies that included more female participants. Additionally, greater age of participants was significantly associated with a weaker association between social support and life satisfaction; however, this subset of studies was highly heterogeneous and may have occurred because there are other variables that moderate the association.

### Comparisons With Similar Research

Our findings extend the literature in three main ways. First, our findings support previous research suggesting that physical capability, social support, loneliness, depression, and anxiety are associated with life satisfaction in older adults (Dong et al., 2020; Elmstahl et al., 2020; Fukuzawa & Sugawara, 2022; Hannaford et al., 2018; Park & Sok, 2020; Puvill et al., 2019; Pynnönen et al., 2021; Rochelle, 2022; Shin & Sok, 2012; Tian & Wang, 2021). Our findings extend this previous knowledge by conducting the first meta-analysis to examine the strength of these associations. Some previous reviews into life satisfaction in older adults focused on partnership status (Stahnke & Cooley, 2020), social cohesion and an age-friendly environment (Nedeljko et al., 2022), effect of brisk walking (Bai et al., 2021), and reminiscence interventions (Wu et al., 2023). Only one previous review (Khodabakhsh, 2021) focused on assessing the factors that contribute to life satisfaction and identified depression, physical capability, and social support as some of the key factors affecting life satisfaction. However, this review solely considered studies conducted in Asia and did not conduct a meta-analysis of the studies. Through meta-analysis, this present study revealed that the strength of the associations between physical capability, social support, loneliness, depression, anxiety, and life satisfaction was high, and depression and anxiety had the strongest association. This finding is important because it highlights mental health as a priority factor for interventions to consider.

Second, country group (Western vs Eastern) was chosen as a moderator as previous research (Hong et al., 2019; Ji et al., 2022) has emphasized the necessity of taking culture into account when investigating life satisfaction in older adults. This is the first time that such a large-scale evaluation of cultural differences has been undertaken in relation to investigating life satisfaction in older adults. Our results revealed that there were no significant differences according to country group. These findings could suggest that the factors that contribute to life satisfaction in older adults are internationally relevant and not strongly influenced by culture. However, it is also possible that this lack of difference could be due to high levels of heterogeneity between included studies.

Previous cross-cultural qualitative studies have examined the meaning of life satisfaction, but they have primarily focused on students rather than older adults (Brailovskaia et al., 2021). Another prior cross-cultural mixed-method study (Thomas & Chambers, 1989) investigated life satisfaction in older adults, incorporating a qualitative technique, but it solely concentrated on older males. In addition, this study did not specifically investigate the factors that contribute to the life satisfaction of older adults, but focused more on the participants’ past as well as present circumstances. Hence, it would be useful to conduct further cross-cultural qualitative work to explore how older adults from Eastern and Western cultures perceive the factors that contribute to life satisfaction. Finally, most of the prior cross-cultural studies that assessed life satisfaction in older adults used quantitative methods (Ji et al., 2022; Schwarz et al., 2010). However, these studies did not focus on the association between life satisfaction and physical capability, social support, loneliness, depression, and anxiety. Therefore, future studies that employ a cross-cultural quantitative approach are needed to investigate the differences between cultures in these associations.

Third, lower levels of loneliness were associated with higher levels of life satisfaction, and gender moderated this association such that it was stronger in studies with more female participants. One possible explanation is that social support is more important to females (Antonucci & Akiyama, 1987; Eagly & Crowley, 1986). Moreover, males tend to be reluctant to confess their emotions of loneliness (Cramer & Neyedley, 1998), so older men may have feelings of loneliness, yet they do not express them or possibly they are able to find satisfaction in life through other factors such as financial well-being (Calasanti et al., 2021). However, due to the high level of heterogeneity in included studies, interpretations of these findings should be cautious.

### Implications

Our findings suggest that public health practitioners, policy-makers, and healthcare professionals who are focused on developing interventions to increase life satisfaction could usefully focus on increasing physical capability and social support, and reducing depression, loneliness, and anxiety in older adults. Interventions that have been found to be useful in impacting these outcomes are integrative reminiscence therapy (Meléndez Moral et al., 2015) for depression and life satisfaction; self-help group intervention (Sahar et al., 2017) for health status physically and mentally; community interventions including educational workshops, mindfulness, yoga, walking and visits to urban gardens for loneliness, social support, depression, and global mental health (Rodríguez-Romero et al., 2021); and restorative home care for physical function (Parsons et al., 2013).

### Strengths

The review executed comprehensive systematic searches and used a rigorous selection approach to ensure the inclusion of eligible and relevant studies. It took an international approach to selecting studies and included a large number of studies from a wide range of countries; therefore, the findings have global relevance.

### Limitations and Future Directions

There were methodological disparities among studies, including variations in study design, target population, and variable measurements, likely to contribute to heterogeneity. In general, the presence of a high level of heterogeneity in each conducted meta-analysis poses challenges in interpreting the findings. Additionally, the assessment of the aforementioned exposures and outcomes was characterized by significant variability in the tools or questionnaires employed. This lack of consistency hindered our ability to conduct meaningful subgroup or sensitivity analyses based on the tools that were used. Moreover, the variation of outcome measures could also be a potential limitation in effect size estimation. It should also be noted that as we included observational studies rather than experimental studies, causality between the factors we treated and independent and dependent variables cannot be demonstrated. Future studies need to consider the above limitations, for example, consensus work should be undertaken to identify gold-standard tools for measuring the variables studied in the review. Additionally, even though our review identified a strong association between anxiety and life satisfaction, available studies were too sparse in number to enable subgroup and moderation analyses. Future studies should focus on understanding this association. Furthermore, the review was limited by its restriction to English language studies, which excluded non-English language studies; this decision may have generated linguistic bias, possibly excluding high-quality studies published in other languages. This limitation was due to limited resources which were available to us for translation. This is common practice within reviews, but it is important, moving forward, that systems are developed that will enable the routine translation and inclusion of such studies in reviews. Future research should consider a larger range of linguistic sources.

## Conclusion

Physical capability, social support, loneliness, depression, and anxiety were significantly associated with life satisfaction in older adults. It is crucial for policy-makers and healthcare professionals to prioritize the implementation of strategies aimed at enhancing the physical capabilities of older individuals, promoting social support, and mitigating the negative impacts of loneliness, depression, and anxiety in both Western and Eastern groups. These endeavors could play a vital role in guaranteeing the overall life satisfaction of older adults.

## Supplementary Material

gnae128_suppl_Supplementary_Tables_S1-S6_Figures_S1-S35

## Data Availability

The data, analytic methods, or materials are available to other researchers for replication purposes, they can be accessed by contacting the authors directly, and the studies reported in the manuscript were preregistered on PROSPERO with registration number CRD42022337584.
