# Supplementary material for: A Systematic Review and Meta-Analysis of the Association Between Physical Capability, Social Support, Loneliness, Depression, Anxiety, and Life Satisfaction in Older Adults
Source: Gerontologist. 2024 Sep 5;64(11):gnae128. doi: 10.1093/geront/gnae128 (PMC11512076; doi:10.1093/geront/gnae128)
Supplement: gnae128_suppl_Supplementary_Tables_S1-S6_Figures_S1-S35 [file gnae128_suppl_supplementary_tables_s1-s6_figures_s1-s35.docx]

**Supplementary Files**

**Supplementary A: PRISMA Checklist (Table S1) and Search strategy (Table S2)**

**Table S1. PRISMA Checklist**

Currently, the page numbers were assigned based on the anonymous file. Nevertheless, the online submission process has the potential to automatically alter it.

| **Section and Topic** | **Item #** | **Checklist item** | **Location where item is reported** |
| --- | --- | --- | --- |
| **TITLE** | | |  |
| Title | 1 | Identify the report as a systematic review. | 1 |
| **ABSTRACT** | | |  |
| Abstract | 2 | See the PRISMA 2020 for Abstracts checklist. | 2 |
| **INTRODUCTION** | | |  |
| Rationale | 3 | Describe the rationale for the review in the context of existing knowledge. | 3-6 |
| Objectives | 4 | Provide an explicit statement of the objective(s) or question(s) the review addresses. | 6 |
| **METHODS** | | |  |
| Eligibility criteria | 5 | Specify the inclusion and exclusion criteria for the review and how studies were grouped for the syntheses. | 7 |
| Information sources | 6 | Specify all databases, registers, websites, organisations, reference lists and other sources searched or consulted to identify studies. Specify the date when each source was last searched or consulted. | 7 |
| Search strategy | 7 | Present the full search strategies for all databases, registers and websites, including any filters and limits used. | 7 |
| Selection process | 8 | Specify the methods used to decide whether a study met the inclusion criteria of the review, including how many reviewers screened each record and each report retrieved, whether they worked independently, and if applicable, details of automation tools used in the process. | 8-9 |
| Data collection process | 9 | Specify the methods used to collect data from reports, including how many reviewers collected data from each report, whether they worked independently, any processes for obtaining or confirming data from study investigators, and if applicable, details of automation tools used in the process. | 8-9 |
| Data items | 10a | List and define all outcomes for which data were sought. Specify whether all results that were compatible with each outcome domain in each study were sought (e.g. for all measures, time points, analyses), and if not, the methods used to decide which results to collect. | 8-11 |
|  | 10b | List and define all other variables for which data were sought (e.g. participant and intervention characteristics, funding sources). Describe any assumptions made about any missing or unclear information. | 8-11 |
| Study risk of bias assessment | 11 | Specify the methods used to assess risk of bias in the included studies, including details of the tool(s) used, how many reviewers assessed each study and whether they worked independently, and if applicable, details of automation tools used in the process. | 12 |
| Effect measures | 12 | Specify for each outcome the effect measure(s) (e.g. risk ratio, mean difference) used in the synthesis or presentation of results. | 10-11 |
| Synthesis methods | 13a | Describe the processes used to decide which studies were eligible for each synthesis (e.g. tabulating the study intervention characteristics and comparing against the planned groups for each synthesis (item #5)). | 8-11 |
|  | 13b | Describe any methods required to prepare the data for presentation or synthesis, such as handling of missing summary statistics, or data conversions. | 8-11 |
|  | 13c | Describe any methods used to tabulate or visually display results of individual studies and syntheses. | 8-11 |
|  | 13d | Describe any methods used to synthesize results and provide a rationale for the choice(s). If meta-analysis was performed, describe the model(s), method(s) to identify the presence and extent of statistical heterogeneity, and software package(s) used. | 8-11 |
|  | 13e | Describe any methods used to explore possible causes of heterogeneity among study results (e.g. subgroup analysis, meta-regression). | 11-12 |
|  | 13f | Describe any sensitivity analyses conducted to assess robustness of the synthesized results. | 11-12 |
| Reporting bias assessment | 14 | Describe any methods used to assess risk of bias due to missing results in a synthesis (arising from reporting biases). | 12 |
| Certainty assessment | 15 | Describe any methods used to assess certainty (or confidence) in the body of evidence for an outcome. | 11-12 |
| **RESULTS** | | |  |
| Study selection | 16a | Describe the results of the search and selection process, from the number of records identified in the search to the number of studies included in the review, ideally using a flow diagram. | 12 |
|  | 16b | Cite studies that might appear to meet the inclusion criteria, but which were excluded, and explain why they were excluded. | 12 |
| Study characteristics | 17 | Cite each included study and present its characteristics. | 9 |
| Risk of bias in studies | 18 | Present assessments of risk of bias for each included study. | 13 |
| Results of individual studies | 19 | For all outcomes, present, for each study: (a) summary statistics for each group (where appropriate) and (b) an effect estimate and its precision (e.g. confidence/credible interval), ideally using structured tables or plots. | 13-17 |
| Results of syntheses | 20a | For each synthesis, briefly summarise the characteristics and risk of bias among contributing studies. | 13-17 |
|  | 20b | Present results of all statistical syntheses conducted. If meta-analysis was done, present for each the summary estimate and its precision (e.g. confidence/credible interval) and measures of statistical heterogeneity. If comparing groups, describe the direction of the effect. | 13-17 |
|  | 20c | Present results of all investigations of possible causes of heterogeneity among study results. | 16-17 |
|  | 20d | Present results of all sensitivity analyses conducted to assess the robustness of the synthesized results. | 13-15 |
| Reporting biases | 21 | Present assessments of risk of bias due to missing results (arising from reporting biases) for each synthesis assessed. | 18 |
| Certainty of evidence | 22 | Present assessments of certainty (or confidence) in the body of evidence for each outcome assessed. | 18 |
| **DISCUSSION** | | |  |
| Discussion | 23a | Provide a general interpretation of the results in the context of other evidence. | 19-20 |
|  | 23b | Discuss any limitations of the evidence included in the review. | 22 |
|  | 23c | Discuss any limitations of the review processes used. | 22 |
|  | 23d | Discuss implications of the results for practice, policy, and future research. | 22 |
| **OTHER INFORMATION** | | |  |
| Registration and protocol | 24a | Provide registration information for the review, including register name and registration number, or state that the review was not registered. | 7 |
|  | 24b | Indicate where the review protocol can be accessed, or state that a protocol was not prepared. | 23 |
|  | 24c | Describe and explain any amendments to information provided at registration or in the protocol. | 7 |
| Support | 25 | Describe sources of financial or non-financial support for the review, and the role of the funders or sponsors in the review. | 23 |
| Competing interests | 26 | Declare any competing interests of review authors. | 23 |
| Availability of data, code and other materials | 27 | Report which of the following are publicly available and where they can be found: template data collection forms; data extracted from included studies; data used for all analyses; analytic code; any other materials used in the review. | 7, 35-100 |

**Table S2. Search strategy in MEDLINE database. For the search strategy in the other databases, this search strategy was adopted according to the respective guidelines of each database.**

| **No.** | **Search Strategy** |
| --- | --- |
| 1 | (depress* or anxiety or anxious*).tw. |
| 2 | Depression/ |
| 3 | Anxiety/ |
| 4 | 1 or 2 or 3 |
| 5 | (physical capabilit* or activit* of daily living* or frailty).tw. |
| 6 | "Activities of Daily Living"/ |
| 7 | Frailty/ |
| 8 | 5 or 6 or 7 |
| 9 | (social support* or famil* support* or neighbo?rhood* support* or communit* support* or friend* support* or lonely or loneliness).tw. |
| 10 | Social Support/ |
| 11 | Loneliness/ |
| 12 | 9 or 10 or 11 |
| 13 | (life satisfaction or satisfaction of life).tw. |
| 14 | Personal Satisfaction/ |
| 15 | 13 or 14 |
| 16 | (older people or elderly or older adult*).tw. |
| 17 | Aged/ |
| 18 | 16 or 17 |
| 19 | 4 or 8 or 12 |
| 20 | 15 and 18 and 19 |
| 21 | (Quantitative or t-test or chi square or covariance or anova or instrument or mean or median or standard deviation or measure or hypothesis or conclusion or result or statistical analysis or survey or experiment or sample or subject* or population or correlation or variable or statistic or association study or cross sectional).tw. |
| 22 | 20 and 21 |
| 23 | limit 22 to English language |

**Supplementary B:** Further detail on inclusion an exclusion criterion

Physical capability was assessed numerically by assessing variables including ADL, frailty, and physical capability or function, which involves assigning scores or ratings based on the individual's ability to perform various daily tasks or a person's ability to perform these tasks independently. Lower scores represented greater dependence and higher scores represented greater independence. For social support, higher scores reflected greater social support. For loneliness, depression, and anxiety, higher scores reflected lower levels of loneliness, depression, and anxiety. For studies that measured these factors in the reverse direction, we transformed them to this numerical standard, for detail of measures used in each study see Supplementary file D, Table S3.

**Supplementary C. Citations for each study**

**C.1. Citation of included study in systematic review**

1. Abu-Bader, S. H., Rogers, A., & Barusch, A. S. (2003). Predictors of life satisfaction in frail elderly. *Journal of Gerontological Social Work, 38*(3), 3-17. https://doi.org/10.1300/J083v38n03_02
2. Adams, T. R., Rabin, L. A., Da Silva, V. G., Katz, M. J., Fogel, J., & Lipton, R. B. (2016). Social support buffers the impact of depressive symptoms on life satisfaction in old age [Article]. *Clinical Gerontologist, 39*(2), 139-157. https://doi.org/10.1080/07317115.2015.1073823
3. Altay, B., & Çalmaz, A. (2023). Perception of loneliness and life satisfaction in the elderly during the COVID-19 pandemic process. *Psychogeriatrics, 23*(1), 177-186. https://doi.org/doi:10.1111/psyg.12911
4. Anaby, D., Miller, W. C., Jarus, T., Eng, J. J., & Noreau, L. (2011). Participation and well-being among older adults living with chronic conditions. *Social Indicators Research*, *100*(1), 171-183. https://doi.org/10.1007/s11205-010-9611-x
5. Aquino, J. A., Russell, D. W., Cutrona, C. E., & Altmaier, E. M. (1996). Employment status, social support, and life satisfaction among the elderly. *Journal of Counseling Psychology, 43*, 480-489.
6. Bai, X., Yang, S., & Knapp, M. (2018). Sources and directions of social support and life satisfaction among solitary Chinese older adults in Hong Kong: the mediating role of sense of loneliness. *Clinical Interventions in Aging, 13*, 63-71. https://doi.org/10.2147/CIA.S148334
7. Berg, A. I., Hassing, L. B., McClearn, G. E., & Johansson, B. (2006). What matters for life satisfaction in the oldest-old?. *Aging & Mental Health, 10*(3), 257-264. https://doi.org/10.1080/13607860500409435
8. Berg, A. I., Hoffman, L., Hassing, L. B., McClearn, G. E., & Johansson, B. (2009). What matters, and what matters most, for change in life satisfaction in the oldest-old? A study over 6 years among individuals 80+. *Aging & Mental Health, 13*(2), 191-201. https://doi.org/10.1080/13607860802342227
9. Berglund, H., Hasson, H., Wilhelmson, K., Duner, A., & Dahlin-Ivanoff, S. (2016). The impact of socioeconomic conditions, social networks, and health on frail older people's life satisfaction: A cross-sectional study. *Health Psychology Research, 4*(1), 5578. https://doi.org/10.4081/hpr.2016.5578
10. Bourque, P., Léger, C., Pushkar, D., & Béland, F. (2007). Self-reported sensory impairment and life satisfaction in older French-speaking adults. *The Canadian journal of nursing research, 39*(4), 155-171. https://pubmed.ncbi.nlm.nih.gov/18277793/
11. Bowling, A., Farquhar, M., Grundy, E., & Formby, J. (1993). Changes in life satisfaction over a two and a half year period among very elderly people living in London. *Social Science & Medicine, 36*(5), 641-655. https://doi.org/https://doi.org/10.1016/0277-9536(93)90061-8
12. Bowling, A. N. N., Farquhar, M., & Grundy, E. (1996). Associations with changes in life satisfaction among three samples of elderly people living at home. *International Journal of Geriatric Psychiatry, 11*(12), 1077-1087. https://doi.org/https://doi.org/10.1002/(SICI)1099-1166(199612)11:12<1077::AID-GPS466>3.0.CO;2-D
13. Bowling, A. P., Edelmann, R. J., Leaver, J., & Hoekel, T. (1989). Loneliness, mobility, well-being and social support in a sample of over 85 year olds. *Personality and Individual Differences, 10*(11), 1189-1192. https://doi.org/https://doi.org/10.1016/0191-8869(89)90085-8
14. Celik, S. S., Celik, Y., Hikmet, N., & Khan, M. M. (2018). Factors affecting life satisfaction of older adults in Turkey. *The International Journal of Aging and Human Development, 87*(4), 392-414. https://doi.org/10.1177/0091415017740677
15. Chang, M., Kim, H., Shigematsu, R., Nho, H., Nishijima, T., & Tanaka, K. (2001). Functional fitness may be related to life satisfaction in older Japanese adults. *The International Journal of Aging and Human Development, 53*(1), 35-49. https://doi.org/10.2190/3xjl-x2yt-2p2c-etef
16. Chen, M., Fu, Y., & Chang, Q. (2021). Life satisfaction among older adults in urban China: does gender interact with pensions, social support and self-care ability? *Ageing and Society, 42*(9), 2026-2045. https://doi.org/10.1017/s0144686x20001877
17. Cheng, A., Leung, Y., Crawford, J. D., Harrison, F., Sachdev, P., & Brodaty, H. (2019). The psychological health of 207 near-centenarians (95-99) and centenarians from the Sydney Centenarian Study. *Australian & New Zealand Journal of Psychiatry, 53*(10), 976-988. https://doi.org/10.1177/0004867419848831
18. Cheng, G., & Yan, Y. (2021). Sociodemographic, health-related, and social predictors of subjective well-being among Chinese oldest-old: a national community-based cohort study. *BMC Geriatrics, 21*(1), 124. https://doi.org/10.1186/s12877-021-02071-7
19. Chou, K. L., & Chi, I. (1999). Determinants of life satisfaction in Hong Kong Chinese elderly: A longitudinal study. *Aging & Mental Health, 3*(4), 328-335. https://doi.org/10.1080/13607869956109
20. Cihlar, V., Micheel, F., & Mergenthaler, A. (2023). Multidimensional vulnerability among older adults in Germany : Social support buffers the negative association with life satisfaction. *Zeitschrift fur Gerontologie und Geriatrie, 56*(8), 654-660. https://doi.org/10.1007/s00391-022-02142-3
21. Dezutter, J., Wiesmann, U., Apers, S., & Luyckx, K. (2013). Sense of coherence, depressive feelings and life satisfaction in older persons: a closer look at the role of integrity and despair. *Aging & Mental Health, 17(*1), 839-843. https://doi.org/10.1080/13607863.2013.792780
22. Didino, D., Taran, E. A., Gorodetski, K., Melikyan, Z. A., Nikitina, S., Gumennikov, I., Korovina, O., & Casati, F. (2018). Exploring predictors of life satisfaction and happiness among Siberian older adults living in Tomsk Region. *European Journal of Ageing, 15*(2), 175-187. https://doi.org/10.1007/s10433-017-0447-y
23. Dumitrache, C. G., Rubio, L., & Cordón-Pozo, E. (2019). Successful aging in Spanish older adults: The role of psychosocial resources [Article]. *International Psychogeriatrics, 31*(2), 181-191. https://doi.org/10.1017/S1041610218000388
24. Dumitrache, C. G., Rubio, L., & Rubio-Herrera, R. (2016). Perceived health status and life satisfaction in old age, and the moderating role of social support. *Aging & Mental Health*, 21(1), 751-757. https://doi.org/10.1080/13607863.2016.1156048
25. Dumitrache, C. G., Rubio, L., & Rubio-Herrera, R. (2018). Extroversion, social support and life satisfaction in old age: a mediation model. *Aging & Mental Health, 22*(8), 1063-1071. https://doi.org/10.1080/13607863.2017.1330869
26. Dumitrache, C. G., Windle, G., & Rubio Herrera, R. (2015). Do social resources explain the relationship between optimism and life satisfaction in community-dwelling older people? Testing a Multiple Mediation Model. *Journal of Happiness Studies, 16*(3), 633-654. https://doi.org/10.1007/s10902-014-9526-3
27. Enkvist, A., Ekstrom, H., & Elmstahl, S. (2012). What factors affect life satisfaction (LS) among the oldest-old? *Archives of Gerontology and Geriatrics, 54*(1), 140-145. https://doi.org/10.1016/j.archger.2011.03.013
28. Erci, B., Yilmaz, D., & Funda, B. (2017). The power of self-care in elderly and life satisfaction hope levels effect. *Journal of Psychiatric Nursing 8*(2):72–76. https://doi.org/10.14744/phd.2017.52714
29. Etxeberria, I., Etxebarria, I., & Urdaneta, E. (2019). Subjective well-being among the oldest old: The role of personality traits. *Personality and Individual Differences, 146*, 209-216. https://doi.org/10.1016/j.paid.2018.04.042
30. Evans, R. J. (2009). A comparison of rural and urban older adults in Iowa on specific markers of successful aging. *Journal of Gerontological Social Work, 52*(4), 423-438. https://doi.org/10.1080/01634370802609197
31. Fukuzawa, A., & Sugawara, I. (2022). Social support and participation as factors relating to ikigai and life satisfaction in lonely older Japanese. *Ageing International, 48*(2), 465-481. <https://doi.org/10.1007/s12126-022-09486-6>
32. Got, a, H., Tsugawa, Y., Xu, H. Y., & Reuben, D. B. (2023). Life satisfaction among persons living with dementia and those without dementia. *Journal of the American Geriatrics Society, 71*(4), 1105-1116. https://doi.org/doi:10.1111/jgs.18174
33. Grenier, S., Desjardins, F., Raymond, B., Payette, M. C., Rioux, M. E., Landreville, P., Gosselin, P., Richer, M. J., Gunther, B., Fournel, M., & Vasiliadis, H. M. (2018). Six-month prevalence and correlates of generalized anxiety disorder among primary care patients aged 70 years and above: Results from the ESA-services study. *International Journal of Geriatric Psychiatry, 34*(2), 315-323. https://doi.org/10.1002/gps.5023
34. Hajek, A., & Konig, H. H. (2021). Determinants of psychosocial factors among the oldest old - Evidence from the representative "Survey on quality of life and subjective well-being of the very old in North Rhine-Westphalia (NRW80+)". *International Journal of Geriatric Psychiatry, 37*(1). https://doi.org/10.1002/gps.5631
35. Harasemiw, O., Newall, N., Mackenzie, C. S., Shooshtari, S., & Menec, V. (2019). Is the association between social network types, depressive symptoms and life satisfaction mediated by the perceived availability of social support? A cross-sectional analysis using the Canadian Longitudinal Study on Aging. *Aging & Mental Health, 23*(10), 1413-1422. https://doi.org/10.1080/13607863.2018.1495176
36. Helvik, A. S., Engedal, K., Krokstad, S., & Selbaek, G. (2011). A comparison of life satisfaction in elderly medical inpatients and the elderly in a population-based study: Nord-Trondelag Health Study 3. *Scandinavian Journal of Public Health, 39*(4), 337-344. https://doi.org/10.1177/1403494811405093
37. Heo, J., Chun, S., Lee, S., Lee, K. H., & Kim, J. (2015). Internet use and well-being in older adults [Article]. *Cyberpsychology, Behavior, and Social Networking, 18*(5), 268-272. https://doi.org/10.1089/cyber.2014.0549
38. Huang, F., & Fu, P. (2021). Intergenerational support and subjective wellbeing among oldest-old in China: the moderating role of economic status [Article]. *BMC Geriatrics, 21*(1), Article 252. https://doi.org/10.1186/s12877-021-02204-y
39. Jung, M., Muntaner, C., & Choi, M. (2010). Factors related to perceived life satisfaction among the elderly in South Korea [Article]. *Journal of Preventive Medicine and Public Health, 43*(4), 292-300. https://doi.org/10.3961/jpmph.2010.43.4.292
40. Kaucic, B. M., Ovsenik, M., & Filej, B. (2019). Is the holistic physical factor linked to life satisfaction in older age?. *Medycyna Pracy, 70*(5), 535-543. https://doi.org/10.13075/mp.5893.00806
41. Kim, S.-Y., & Sok, S. (2013). Factors influencing the life satisfaction in the older Korean women living alone. *Contemporary Nurse, 44*(1), 111-119.
42. Ko, H., & Jung, S. (2021). Association of social frailty with physical health, cognitive function, psychological health, and life satisfaction in community-dwelling older Koreans. *International Journal of Environmental Research and Public Health, 18*(2). <https://doi.org/10.3390/ijerph18020818>
43. Koong, H. S., Lim, M., & Seo, K. (2022). Dual mediating effect of electronic device utilization and life satisfaction on the relationship between instrumental activities of daily living and depression in older adults*. International Journal of Environmental Research & Public Health [Electronic Resource], 19*(17), 25. https://doi.org/doi:https://dx.doi.org/10.3390/ijerph191710617
44. Krause, N. (2004). Lifetime trauma, emotional support, and life satisfaction among older adults. *The Gerontologist, 44*(5), 615-623. <https://doi.org/10.1093/geront/44.5.615>
45. Kwon, M., Moon, W. H., & Kim, S. A. (2023). Mediating effect of life satisfaction and depression on the relationship between cognition and activities of daily living in Korean male older adults. *Journal of Men's Health, 19*, 73-81. <https://doi.org/doi:https://dx.doi.org/10.22514/jomh.2023.118>
46. Lai, C. Y. Y., Chen, L. H., Lai, F. H. Y., Fung, A. W. T., & Ng, S. S. M. (2023). The association between satisfaction with life and anxiety symptoms among Chinese elderly: a moderated mediation analysis. *BMC Geriatrics, 23*(1), 855. <https://doi.org/doi:https://dx.doi.org/10.1186/s12877-023-04490-0>
47. Lalani, N., Dongjuan, X., Cai, Y., & Arling, G. W. (2023). Structural equation model of coping and life satisfaction of community-dwelling older people during the COVID-19 pandemic. *Journal of Patient-Reported Outcomes, 7*(1), 46. https://doi.org/doi:https://dx.doi.org/10.1186/s41687-023-00583-x
48. Lara, R., Vazquez, M. L., Ogallar, A., & Godoy-Izquierdo, D. (2020). Psychosocial resources for hedonic balance, life satisfaction and happiness in the elderly: A Path Analysis. *International Journal of Environmental Research and Public Health*, *17*(16). https://doi.org/10.3390/ijerph17165684
49. Lee, E.-K. O., & Lee, J. (2013). Education, Functional Limitations, and Life Satisfaction Among Older Adults in South Korea. *Educational Gerontology, 39*(1), 514-526. https://doi.org/10.1080/03601277.2012.701154
50. Lee, H. J., Lyu, J., Lee, C. M., & Burr, J. A. (2014). Intergenerational financial exchange and the psychological well-being of older adults in the Republic of Korea [Article]. *Aging and Mental Health, 18*(1), 30-39. <https://doi.org/10.1080/13607863.2013.784955>
51. Lee, E., & Williams, J. M. (2023). Life satisfaction and depression among older adults during covid-19: examining awareness and use of community mental health welfare centers. Journal of Social Service Research, 49(6), 653-664. https://doi.org/doi:10.1080/01488376.2023.2263479
52. Liu, L. H., Kao, C. C., & Ying, J. C. (2020). Functional capacity and life satisfaction in older adult residents living in long-term care facilities: The Mediator of Autonomy. *Journal of Nursing Research, 28*(4), e102. https://doi.org/10.1097/jnr.0000000000000362
53. Liu, Z., Wu, D., Huang, J., Qian, D., Chen, F., Xu, J., Li, S., Jin, L., & Wang, X. (2016). Visual impairment, but not hearing impairment, is independently associated with lower subjective well-being among individuals over 95 years of age: A population-based study. *Archives of Gerontology and Geriatrics, 62*, 30-35. https://doi.org/10.1016/j.archger.2015.10.011
54. Lu, L., & Chang, C.-J. (1997). Social support, health and satisfaction among the elderly with chronic conditions in Taiwan. *Journal of Health Psychology, 2*(4), 471-480.
55. Lu, N., Spencer, M., Sun, Q., & Lou, V. W. Q. (2020). Family social capital and life satisfaction among older adults living alone in urban China: the moderating role of functional health [Article]. *Aging and Mental Health, 25*(4), 695-702. <https://doi.org/10.1080/13607863.2019.1709155>
56. Luo, M., Kim, E.-K., Weibel, R., Martin, M., & Röcke, C. (2023). GPS-derived daily mobility and daily well-being in community-dwelling older adults. *Gerontology, 69*(7), 875-887. https://doi.org/10.1159/000527827
57. Mellor, K. S., & Edelmann, R. J. (1988). Mobility, social support, loneliness and well-being amongst two groups of older adults. *Personality and Individual Differences, 9*(1), 1-5. https://doi.org/10.1016/0191-8869(88)90024-4
58. Newsom, J. T., & Schulz, R. (1996). Social support as a mediator in the relation between functional status and quality of life in older adults. *Psychology and aging, 11*(1), 34-44. https://doi.org/10.1037/0882-7974.11.1.34
59. Ni Mhaolain, A. M., Gallagher, D., H, O. C., Chin, A. V., Bruce, I., Hamilton, F., Teehee, E., Coen, R., Coakley, D., Cunningham, C., Walsh, J. B., & Lawlor, B. A. (2012). Subjective well-being amongst community-dwelling elders: what determines satisfaction with life? Findings from the Dublin Healthy Aging Study. *International Psychogeriatrics, 24*(2), 316-323. https://doi.org/10.1017/S1041610211001360
60. Nilsson, I., Bernspång, B., Fisher, A. G., Gustafson, Y., & Löfgren, B. (2007). Occupational engagement and life satisfaction in the oldest-old: The Umeå 85+ study. OTJR: *Occupational Therapy Journal of Research, 27*(4), 131-139. https://doi.org/10.1177/153944920702700403
61. Noh, J.-W., Kim, K.-B., Lee, J. H., Kim, M. H., & Kwon, Y. D. (2017). Relationship of health, sociodemographic, and economic factors and life satisfaction in young-old and old-old elderly: a cross-sectional analysis of data from the Korean Longitudinal Study of Aging. *Journal of Physical Therapy Science, 29*(9), 1483-1489. https://doi.org/10.1589/jpts.29.1483
62. Onal, O., Evcil, F. Y., Dogan, E., Develi, M., Uskun, E., & Kisioglu, A. N. (2022). The effect of loneliness and perceived social support among older adults on their life satisfaction and quality of life during the COVID-19 pandemic. *Educational Gerontology, 48*(1), 331-343. https://doi.org/10.1080/03601277.2022.2040206
63. Park, S. (2024). Impact of social support on life satisfaction in older adults: Considering socioeconomic status as moderator. Asian Journal of Social Psychology, 27(1), 90-100. https://doi.org/doi:10.1111/ajsp.12583
64. Park, S., & Sok, S. R. (2020). Relation modeling of factors influencing life satisfaction and adaptation of korean older adults in long-term care facilities. *International Journal of Environmental Research & Public Health*, *17*(1). <https://doi.org/10.3390/ijerph17010317>
65. Perkins, E. A., Small, B. J., Balducci, L., Extermann, M., Robb, C., & Haley, W. E. (2007). Individual differences in well-being in older breast cancer survivors. *Critical Reviews in Oncology/Hematology, 62*(1), 74-83. https://doi.org/10.1016/j.critrevonc.2006.11.002
66. Pinto, J. M., Fontaine, A. M., & Neri, A. L. (2016). The influence of physical and mental health on life satisfaction is mediated by self-rated health: A study with Brazilian elderly. *Archives of Gerontology and Geriatrics, 65*, 104-110. https://doi.org/10.1016/j.archger.2016.03.009
67. Pynnönen, K., Kokko, K., Saajanaho, M., Törmäkangas, T., Portegijs, E., & Rantanen, T. (2021). Do opposite ends of same factors underlie life satisfaction vs. depressive symptoms among older people?. *Aging Clinical and Experimental Research, 33*(9), 2557-2564. https://doi.org/10.1007/s40520-020-01765-z
68. Rochelle, T. L. (2022). Social participation, loneliness and well-being among older adults in Hong Kong: a longitudinal examination. *Psychology, Health & Medicine*, 1-11. https://doi.org/10.1080/13548506.2022.2058028
69. Roh, S., Lee, Y. S., Lee, K. H., Shibusawa, T., & Yoo, G. J. (2015). Friends, depressive symptoms, and life satisfaction among older Korean Americans. *Journal of Immigrant and Minority Health, 17*(4), 1091-1097. https://doi.org/10.1007/s10903-014-0021-z
70. Şahin, D. S., Özer, Ö., & Yanardağ, M. Z. (2019). Perceived social support, quality of life and satisfaction with life in elderly people. *Educational Gerontology, 45*(1), 69-77. https://doi.org/10.1080/03601277.2019.1585065
71. Schilling, O. K., Wahl, H.-W., & Oswald, F. (2013). Change in life satisfaction under chronic physical multi-morbidity in advanced old age: Potential and limits of adaptation. *Journal of Happiness Studies, 14*(1), 19-36. https://doi.org/10.1007/s10902-011-9313-3
72. Sexton, E., Bennett, K., Fahey, T., & Cahir, C. (2017). Does the EQ-5D capture the effects of physical and mental health status on life satisfaction among older people? A path analysis approach. *Quality of Life Research, 26*(5), 1177-1186. https://doi.org/10.1007/s11136-016-1459-3
73. Shin, S. H., & Sok, S. R. (2012). A comparison of the factors influencing life satisfaction between Korean older people living with family and living alone. *International Nursing Review, 59*(2), 252-258. https://doi.org/https://doi.org/10.1111/j.1466-7657.2011.00946.x
74. Tavares, A. I. (2022). Health and life satisfaction factors of Portuguese older adults [Article]. *Archives of Gerontology and Geriatrics, 99*, Article 104600. https://doi.org/10.1016/j.archger.2021.104600
75. Tumer, A., Donmez, S., Gumussoy, S., & Balkaya, N. A. (2021). The relationship among aging in place, loneliness, and life satisfaction in the elderly in Turkey. *Perspectives in Psychiatric Care, 58*(2), 822-829. https://doi.org/10.1111/ppc.12855
76. Wang, C. W., Iwaya, T., Kumano, H., Suzukamo, Y., Tobimatsu, Y., & Fukudo, S. (2002). Relationship of health status and social support to the life satisfaction of older adults. *The Tohoku Journal of Experimental Medicine, 198*(3), 141-149. https://doi.org/10.1620/tjem.198.141
77. Wangliu, Y. (2023). Does intergenerational support affect older people's social participation? an empirical study of an older Chinese population. *SSM - Population Health, 22*, 101368. https://doi.org/doi:https://dx.doi.org/10.1016/j.ssmph.2023.101368
78. Windle, G., & Woods, R. T. (2004). Variations in subjective wellbeing: the mediating role of a psychological resource. *Ageing and Society, 24*(4), 583-602. <https://doi.org/10.1017/s0144686x04002107>

**C.2. Citation of excluded studies for the meta-analysis**

1. Adams, T. R., Rabin, L. A., Da Silva, V. G., Katz, M. J., Fogel, J., & Lipton, R. B. (2016). Social support buffers the impact of depressive symptoms on life satisfaction in old age [Article]. *Clinical Gerontologist, 39*(2), 139-157. https://doi.org/10.1080/07317115.2015.1073823
2. Berglund, H., Hasson, H., Wilhelmson, K., Duner, A., & Dahlin-Ivanoff, S. (2016). The impact of socioeconomic conditions, social networks, and health on frail older people's life satisfaction: A cross-sectional study. *Health Psychology Research, 4*(1), 5578. https://doi.org/10.4081/hpr.2016.5578
3. Bowling, A. N. N., Farquhar, M., & Grundy, E. (1996). Associations with changes in life satisfaction among three samples of elderly people living at home. *International Journal of Geriatric Psychiatry, 11*(12), 1077-1087. https://doi.org/https://doi.org/10.1002/(SICI)1099-1166(199612)11:12<1077::AID-GPS466>3.0.CO;2-D
4. Cihlar, V., Micheel, F., & Mergenthaler, A. (2023). Multidimensional vulnerability among older adults in Germany : Social support buffers the negative association with life satisfaction. *Zeitschrift fur Gerontologie und Geriatrie, 56*(8), 654-660. https://doi.org/10.1007/s00391-022-02142-3
5. Dumitrache, C. G., Rubio, L., & Cordón-Pozo, E. (2019). Successful aging in Spanish older adults: The role of psychosocial resources [Article]. *International Psychogeriatrics, 31*(2), 181-191. https://doi.org/10.1017/S1041610218000388
6. Dumitrache, C. G., Rubio, L., & Rubio-Herrera, R. (2016). Perceived health status and life satisfaction in old age, and the moderating role of social support. *Aging & Mental Health, 21*(1), 751-757. https://doi.org/10.1080/13607863.2016.1156048
7. Dumitrache, C. G., Windle, G., & Rubio Herrera, R. (2015). Do social resources explain the relationship between optimism and life satisfaction in community-dwelling older people? Testing a multiple mediation model. *Journal of Happiness Studies, 16*(3), 633-654. <https://doi.org/10.1007/s10902-014-9526-3>
8. Got, a, H., Tsugawa, Y., Xu, H. Y., & Reuben, D. B. (2023). Life satisfaction among persons living with dementia and those without dementia. *Journal of the American Geriatrics Society, 71*(4), 1105-1116. https://doi.org/doi:10.1111/jgs.18174
9. Heo, J., Chun, S., Lee, S., Lee, K. H., & Kim, J. (2015). Internet use and well-being in older adults [Article]. *Cyberpsychology, Behavior, and Social Networking, 18*(5), 268-272. https://doi.org/10.1089/cyber.2014.0549
10. Huang, F., & Fu, P. (2021). Intergenerational support and subjective wellbeing among oldest-old in China: the moderating role of economic status [Article]. *BMC Geriatrics, 21*(1), Article 252. https://doi.org/10.1186/s12877-021-02204-y
11. Jung, M., Muntaner, C., & Choi, M. (2010). Factors related to perceived life satisfaction among the elderly in South Korea [Article]. *Journal of Preventive Medicine and Public Health, 43*(4), 292-300. https://doi.org/10.3961/jpmph.2010.43.4.292
12. Kaucic, B. M., Ovsenik, M., & Filej, B. (2019). Is the holistic physical factor linked to life satisfaction in older age?. *Medycyna Pracy, 70*(5), 535-543. https://doi.org/10.13075/mp.5893.00806
13. Krause, N. (2004). Lifetime trauma, emotional support, and life satisfaction among older adults. *The Gerontologist*, 44(5), 615-623. https://doi.org/10.1093/geront/44.5.615
14. Lalani, N., Dongjuan, X., Cai, Y., & Arling, G. W. (2023). Structural equation model of coping and life satisfaction of community-dwelling older people during the COVID-19 pandemic. *Journal of Patient-Reported Outcomes, 7*(1), 46. <https://doi.org/doi:https://dx.doi.org/10.1186/s41687-023-00583-x>
15. Lee, E.-K. O., & Lee, J. (2013). Education, Functional Limitations, and Life Satisfaction Among Older Adults in South Korea. *Educational Gerontology, 39*(1), 514-526. https://doi.org/10.1080/03601277.2012.701154
16. Lee, H. J., Lyu, J., Lee, C. M., & Burr, J. A. (2014). Intergenerational financial exchange and the psychological well-being of older adults in the Republic of Korea [Article]. *Aging &Mental Health, 18*(1), 30-39. https://doi.org/10.1080/13607863.2013.784955
17. Lu, N., Spencer, M., Sun, Q., & Lou, V. W. Q. (2020). Family social capital and life satisfaction among older adults living alone in urban China: the moderating role of functional health [Article]. *Aging & Mental Health, 25*(4), 695-702. https://doi.org/10.1080/13607863.2019.1709155
18. Park, S., & Sok, S. R. (2020). Relation modeling of factors influencing life satisfaction and adaptation of Korean older adults in long-term care facilities. *International Journal of Environmental Research and Public Health, 17*(1). https://doi.org/10.3390/ijerph17010317
19. Shin, S. H., & Sok, S. R. (2012). A comparison of the factors influencing life satisfaction between Korean older people living with family and living alone. *International Nursing Review, 59*(2), 252-258. https://doi.org/https://doi.org/10.1111/j.1466-7657.2011.00946.x
20. Tavares, A. I. (2022). Health and life satisfaction factors of Portuguese older adults [Article]. *Archives of Gerontology and Geriatrics, 99*, Article 104600. <https://doi.org/10.1016/j.archger.2021.104600>
21. Wangliu, Y. (2023). Does intergenerational support affect older people's social participation? An empirical study of an older Chinese population. SSM - Population Health, 22, 101368. https://doi.org/doi:https://dx.doi.org/10.1016/j.ssmph.2023.101368

**Supplementary D: Details of data extraction**

**Table S3. Information of the data extraction (Part 1)**

| **Study ID** | **Author (year)** | **Country** | **Setting** | **Outcome** | **Relevant predictors’ measure** | | | **Study design** | **Age  (years)** | **Mean age** | **Sample size** |
| --- | --- | --- | --- | --- | --- | --- | --- | --- | --- | --- | --- |
|  |  |  |  | **Life satisfaction measure** | **Mental health measure** | **Physical capability measure** | **Social support measure** |  |  |  |  |
|  | Abu-Bader et al., 2003 | United States | Community-dwelling | LSI-Z (14 items) | NA | ADL (The index of ADL); IADL (NR); Mobility: sub-scale from Iowa Self-Assessment Inventory | Social support: sub-scale from Iowa Self-Assessment Inventory | Cross-sectional | > 65 | 78.00 | 99 |
|  | Adams et al., 2016 | United States | Community-dwelling | SWLS | GDS-Short form; The Adult Manifest Anxiety Scale–Elderly version (AMAS-E) | NA | The self-report Medical Outcomes Study (MOS) | Cross-sectional | > 70 | 80.80 | 237 |
|  | Altay & Çalmaz, 2023 | Turkiye | Community-dwellling | Life Satisfaction Scale (LSS) | NA | NA | Loneliness: Loneliness Scale for the Elderly (LSE) | Cross-sectional | 65–86 | 70.15 | 216 |
|  | Anaby et al., 2011 | Canada | Community-dwelling | SWLS | NA | Mobility: sub-scale from Assessment of Life habits (LIFE-H) | The Interpersonal Support Evaluation List (ISEL) | Cross-sectional | 65 - 90 | 75.00 | 200 |
|  | Aquino et al., 1996 | United States | Community-dwelling | 10 items based on factor analysis reported by Lohman (1980) | Zung Self-Rating Depression Scale | NA | The Social Provisions Scale | Cross-sectional | 65-97 | 71.90 | 292 |
|  | Bai et al., 2018 | China | Community-dwelling | SWLS | NA | The Chinese version of the seven-item Lawton Instrumental Activities of Daily Living (IADL) | Chinese version of the Sense of loneliness Scale | Cross-sectional | > 65 | 79.60 | 151 |
|  | Berg et al., 2006 | Sweden | Community-dwelling | Life satisfaction Index-Z (13-item version) | Depression: The Centre for Epidemiologic Studies Depression Scale (CES-D) | ADL: IADL and PADL (Lawton scale) | Social support: social support as one of the sub-scales in social network measure | Cross-sectional | 80-98 | 83.00 | 315 |
|  | Berg et al., 2009 | Sweden | Community-dwelling | Life satisfaction Index-Z (13-item version) | CES-D | NA | NA | Longitudinal | > 80 | 83.00 | 412 |
|  | Berglund et al., 2016 | Sweden | Community-dwelling | LiSat-11 scale | Geriatric Depression Scale (GDS)-Swedish modification | Sonn’s ADL staircase | NA | Cross-sectional | > 65 | NR | 179 |
|  | Bourque et al., 2007 | Canada | Community-dwelling | Single-item measure "Are you satisfied with your life in general?" | NA | Rosow-Breslau Functional Health Index & five items from the Physical Performance Scale | The social support measure (Seeman and Berkman, 1988) | Cross-sectional | > 65 | 74.38 | 826 |
|  | Bowling et al., 1989 | United Kingdom | Community-dwelling | LSI-A | NA | The Mobility Scale | A single Likert rating for loneliness | Cross-sectional | > 85 | NR | 662 |
|  | Bowling et al., 1993 | United Kingdom | Community-dwelling | Neugarten’s Life Satisfaction Scale | NA | Activities of Daily Living Scale | has someone would help if needed; "has someone would understand" | Longitudinal | > 85 | NR | 640 |
|  | Bowling et al., 1996 | United Kingdom | Community-dwelling | Neugarten’s Life Satisfaction Scale | NA | The functional ability scale | NA | Longitudinal | > 65 | NR | 1,370 |
|  | Celik et al., 2017 | Turkey | Community-dwelling | Using three variable from Turkey Health Survey (Borenstein) | "Feeling of depression" derived from section in THS | "Personal care competency" & "Household activities functionality" derived from section in THS | NA | Cross-sectional | 65-74 | NR | 2,959 |
|  | Chang et al., 2001 | Japan | Community-dwelling | The life satisfaction scale for older Japanese adults | NA | ADL measurement (Kim & Tanaka, 1994; Kim & Tanaka, 1995) | NA | Cross-sectional | > 65 | 74.50 | 123 |
|  | Chen et al., 2021 | China | Community-dwelling | SWLS-Chinese version | ADL and IADL scales | NA | NA | Cross-sectional | > 65 | 72.90 | 2047 |
|  | Cheng & Yan, 2021 | China | Community-dwelling | Single-item measure “how do you rate your life at present” | Using item in the affective aspects measure | NA | Using item in the affective aspects measure | Prospective cohort study | > 80 | NR | 30,317 |
|  | Cheng et al., 2019 | Australia | Private dwelling, assisted living, and others | Satisfaction with life scale (SWLS) | NA | ADL, Bayer-ADL | NA | Cross-sectional | > 70 | 87.95 | 1239 |
|  | Chou and Chi, 1999 | China | Community-dwelling | LSI-A | CES-D | Physical impartment: mobility items (Rosow-Breslow scale), IADL (Lawton &Brody), Performance items (Nagi index) | NA | Longitudinal | > 70 | 79.10 | 260 |
|  | Dezutter et al., 2013 | Belgium | Community-dwelling | SWLS | CES-D | NA | NA | Cross-sectional | 65-95 | 76.50 | 100 |
|  | Cihlar et al., 2023 | Germany | Community-dwellling | Measured using an 11-point scale from 0 (“not at all satisfied”) to 10 (“completely satisfied”) | NA | NA | Social support: the Oslo 3-item social support scale | Cross-sectional | > 65 | NR | 5826 |
|  | Didino et al., 2018 | Russia | Community-dwelling | Single-item measure “Taking all things together, how happy would you say you are?” | NA | NA | Single-item measure “To what extent do you receive help and support from people you are close to when you need?” | Cross-sectional | 65 | 74.40 | 489 |
|  | Dumitrache et al., 2015 | Spain | Community-dwelling | SWLS-Spanish version | NA | NA | Medical Outcomes Study Social Support Survey Instrument (MOS) | Cross-sectional | 65-99 | 74.88 | 406 |
|  | Dumitrache et al., 2016 | Spain | Community-dwelling | SWLS-Spanish version | NA | NA | Medical Outcomes Study Social Support Survey Instrument (MOS) | Cross-sectional | 65-99 | 74.88 | 406 |
|  | Dumitrache et al., 2018 | Spain | Community-dwelling | SWLS-Spanish version | NA | NA | Medical Outcomes Study Social Support Survey Instrument (MOS) | Cross-sectional | 65-99 | 74.88 | 406 |
|  | Dumitrache et al., 2019 | Spain | Community-dwelling | SWLS-Spanish version | NA | NA | Medical Outcomes Study Social Support Survey Instrument (MOS) | Cross-sectional | 65-99 | 74.88 | 406 |
|  | Enkvist et al., 2012 | Sweden | Community-dwelling | LSI-A | Depression: The Comprehensive Psychiatric Rating Scale (CPRS) | ADL and IADL scales | NA | Longitudinal baseline predictor to LS baseline | 78–93 | 83.10 | 681 |
|  | Erci et al., 2017 | Turkey | Family health-care centre | Life satisfaction scale-adapted to Turkish by Köker | Self-care ability scale | NA | NA | Cross-sectional | 65-69 | NR | 320 |
|  | Etxeberria et al., 2018 | Spain | Community-dwelling | The Spanish version of the Life Satisfaction Scale | NA | BADL: The Barthel Index (BI); IADL: Lawton Instrumental Activities of Daily Living Scale | Functional Social Support Questionnaire (DUKE-UNK-11) | Cross-sectional | 85-104 | 92.53 | 102 |
|  | Evans, 2009 | United States | Community-dwelling | Life Satisfaction Index–Z (LSI-Z) | NA | NA | Duke Social Support Index (DSSI) | Cross-sectional | > 65 | 79.58 | 140 |
|  | Fukuzawa & Sugawara, 2022 | Japan | Community-dwelling | Single-item measure "“Overall, I am satisfied with my current life" | NA | NA | Social support questions and a direct measure of loneliness, “I feel isolated from others," | Cross-sectional | > 75 | 79.06 | 418 |
|  | Got et al., 2023 | United States | Community-dwellling | Satisfaction with Life Scale (SWLS) | Center for Epidemiological Studies Depression (CES-D) | Basic activities of daily living (BADL) limitation & instrumental activities of daily living (IADL) limitation | NA | Cross-sectional (using data from existing longitudinal study) | > 70 | NR | 5871 |
|  | Grenier et al., 2018 | Canada | Community-dwelling | SWLS | Anxiety: The computer-assisted ESA diagnostic Questionnaire (ESA-Q) | NA | NA | Cross-sectional | > 70 | 75.60 | 1193 |
|  | Hajek & Konig, 2021 | Germany | Community-dwelling | Single-item measure | The “depression in old age scale” (DIA‐S) | Functional impartment: IADL | a single item measure loneliness | Cross-sectional | > 80 | 85.80 | 952 |
|  | Harasemiw et al., 2019 | Canada | Community-dwelling | SWLS | CESD-10 scale | NA | Sub-scales of MOS | Cross-sectional (using baseline data from existing longitudinal study) | 65-85 | NR | 8,782 |
|  | Helvik et al., 2011 | Norway | Community-dwelling | Single-item measure ‘When you think about your present situation, are you on the whole satisfied or dissatisfied with your life?’ | Hospital Anxiety and Depression scale (HAD) | NA | NA | Cross-sectional | 65-101 | 80.70 | 10,958 |
|  | Heo et al., 2015 | United States | Community-dwelling | SWLS | NA | NA | Loneliness: 11 items developed by Hughes et al (2004) | Cross-sectional (using data from existing longitudinal study) | 65-105 | 77.87 | 5,203 |
|  | Huang & Fu, 2021 | China | Community-dwelling | Part of Chinese longitudinal Healthy Longevity Survey (CLHLS) | NA | Part of Chinese longitudinal Healthy Longevity Survey (CLHLS) | Part of Chinese longitudinal Healthy Longevity Survey (CLHLS) | Cross-sectional (using data from existing longitudinal study) | > 80 | 91.46 | 8,794 |
|  | Jung et al., 2010 | Korea | Community-dwelling | General life satisfaction questions | NA | Questions regarding the ADL | NA | Cross-sectional | > 65 | 74.30 | 3,278 |
|  | Kaucic et al., 2019 | Slovenia | Social care institutions & community-dwelling | SWLS-Chinese version | NA | Perform daily basic and instrumental activities index | NA | Cross-sectional | > 65 | NR | 656 |
|  | Kim & Sok, 2013 | Korea | Community-dwelling | The Life Satisfaction Instrument developed by Yun (1982) | The Korean Geriatric Depression Scale Short Form | NA | NA | Cross-sectional | > 65 | NR | 243 |
|  | Ko & Jung, 2021 | Korea | Residential facility | Life satisfaction questions | 15-item Geriatric Depression Scale-Short Form Korean Version (GDSSF-K) | NA | NA | Cross-sectional | > 65 | 75.50 | 10,081 |
|  | Koong et al., 2023 | Korea | Not specified | Seven items life satisfaction scale | Korean short version of the Geriatric Depression Scale (SGDS-K) | Korean instrumental activities of daily living (K-IADL) | NA | Cross-sectional | > 65 | 73.68 | 9906 |
|  | Krause, 2004 | United States | Community-dwelling | A brief four-item measure of life satisfaction | NA | NA | Four indicators of emotional support | Longitudinal | > 65 | 74.70 | 1,397 |
|  | Kwon et al., 2023 | Korea | Not specified | Seven items life satisfaction scale | Korean short version of the Geriatric Depression Scale (SGDS-K) | Korean Activities of Daily Living (K-ADL) | NA | Cross-sectional | > 65 | NR | 4035 |
|  | Lai et al., 2023 | China | Community-dwellling | Satisfaction with Life Scale (SWLS) | Hamilton Anxiety Rating Scale (HAM‐A) | NA | NA | Cross-sectional | > 65 | 70.96 | 67 |
|  | Lalani et al., 2023 | United States | Community-dwellling | Diener’s Satisfaction with Life scale | NA | IADL difficulty was measured by the count of domains with any difficulty | NA | Cross-sectional (using data from existing longitudinal study) | 65–74 | NR | 1351 |
|  | Lara et al., 2020 | Spain | Any setting | The Life Satisfaction Scale (LSS) | NA | NA | The MOS Social Support Scale (Lawton et al.) | Cross-sectional | 65-96 | NR | 154 |
|  | Lee & Lee, 2013 | Korea | Community-dwelling | A composite life satisfaction scale | CES-D-short version 10 items | NA | NA | Cross-sectional (using data from existing longitudinal study) | > 65 | 73.84 | 4152 |
|  | Lee et al., 2014 | Korea | Community-dwelling | Single-item measure in general, "how satisfied are you with your quality of life when compared to others in your age group?’ | CES-D 10 items | ADL/IADL limitation's categories | NA | Cross-sectional | > 65 | 72.90 | 3,791 |
|  | Lee & Williams, 2023 | Korea | Community-dwellling | 10-items life satisfaction measure | Center for Epidemiological Studies-Depression (CES-D-11) | NA | NA | Cross-sectional | 66-85 | NR | 545 |
|  | Liu et al., 2016 | China | Community-dwelling | LSI-A | NA | Katz Index of Activities of Daily Living [ADL] | NA | Cross-sectional | 95-107 | 97.50 | 442 |
|  | Liu et al., 2020 | China | Long-term care facility | Single-item measure "“How do you feel about your life in the past 2 weeks?” | NA | Barthel ADL Index | NA | Cross-sectional | 65-100 | 76.76 | 228 |
|  | Lu & Chang, 1997 | Taiwan | Community-dwelling | 5-point ‘faces’ scales (Andrews & Withey, 1976) | NA | ADL & IADL (Fillenbaum, 1988) | Adopting Hanson & colleagues' (1989) questionnaire | Cross-sectional | > 65 | 70.79 | 172 |
|  | Lu et al., 2020 | China | Community-dwelling | Single-item measure “How satisfied are you with your current life?” | NA | Barthel Index | NA | Cross-sectional | > 70 | 78.80 | 2775 |
|  | Luo et al., 2023 | Switzerland | Community-dwellling | Single item measure: “How satisfied are you with your life today”? | NA | Physical functioning question, six items from the scales of the ADL and two items from the SF-12 | NA | Experimental design | 65–89 | 73.16 | 109 |
|  | Mellor & Edelmann, 1988 | United Kingdom | Residential house and community dwelling | LSI-B | NA | The Mobility Scale | The UCLA Loneliness Scale (LS) | Cross-sectional | 67-97 | 81.50 | 36 |
|  | Newsom & Schulz, 1996 | United States | Community-dwelling | Two-items measure | A 10-item version of the CES-D | The IADL, exercise tolerance, upper extremity, and timed walk measures | A 6-item version of the Interpersonal Support Evaluation List (ISEL) | Cross-sectional (using baseline sample from existing longitudinal study) | > 65 | 72.80 | 4,734 |
|  | Ni Mhaolain et al., 2011 | Ireland | Community-dwelling | Life satisfaction index (LSI-A) | CES-D | Lawton IADL | loneliness: "I feel lonely" item | Cross-sectional | > 65 | 75.45 | 466 |
|  | Nilsson et al., 2007 | Sweden | Community-dwelling | Life satisfaction questionnaire (LiSat-9) | NA | ADL Taxonomy | NA | Cross-sectional | 85-98 | NRC | 156 |
|  | Noh et al., 2017 | Korea | Community-dwelling | Single-item measure “On the scale, please point out which point best represents your overall life satisfaction level.” | CES-D (translated to Korean) | K-ADL: Korean version of activities of daily living | NA | Cross-sectional (using data from existing longitudinal study) | > 65 | 74.50 | 4134 |
|  | Onal et al., 2022 | Turkey | Community-dwelling | Turkish Version of the Satisfaction with Life Scale (SWLS-T) | NA | NA | Social support: MSPSS &loneliness: LSE | Cross-sectional | > 65 | 72.21 | 686 |
|  | Park, 2024 | Korea | Not specified | Single item measure: "In general, how satisfied are you with your life?" | NA | NA | 15 items measured three types of social support | Longitudinal study | 65-96 | 75.47 | 2837 |
|  | Park & Sok, 2020 | Korea | Elderly care facilities | MUNSH measure the degree of life satisfaction | KHSME | NA | NA | Cross-sectional | > 65 | 82.26 | 229 |
|  | Perkins et al., 2007 | United States | Community-dwelling | Life satisfaction Index-Z (13-item version) | NA | The Physical impartment subscale of the Medical Outcomes Study (SF-36) | Social support: support received from others | Cross-sectional | > 70 (78.2) | 78.23 | 127 |
|  | Pinto et al., 2016 | Brazil | Community-dwelling | A question concerning level of overall life satisfaction and five other items concerning domain referenced satisfaction | Geriatric Depression Scale (GDS-15) | NA | NA | Cross-sectional | > 65 | 72.70 | 2,164 |
|  | Pynnönen et al., 2021 | Finland | Community-dwelling | SWLS | CES-D 20 items | Short Physical Performance Battery (SPPB) | Single-item measure “How often do you feel lonely?” | Cross-sectional | > 75 | NR | 1021 |
|  | Rochelle, 2022 | China | Elderly centre and NGO | SWLS | NA | NA | The Revised UCLA (R-UCLA) Loneliness Scale | longitudinal | 65-93 | 76.03 | 200 |
|  | Roh et al., 2015 | United States | Community-dwelling | SWLS-Korean version | GDS-Korean version | NA | NA | Cross-sectional | > 65 | 72.50 | 200 |
|  | Sahin et al., 2019 | Turkey | Community-dwelling | SWLS | NA | NA | Multidimensional Scale of Perceived Social Support (MSPSS) | Cross-sectional | > 65 | 72.50 | 517 |
|  | Schilling et al., 2013 | Germany | Community-dwelling | Single-item measure asking for present satisfaction with life | NA | The ADL Staircase (Sonn and Hulter-A ̊ sberg 1991) | NA | Longitudinal | > 80 | NR | 451 |
|  | Sexton et al., 2017 | Ireland | Community-dwelling | Life satisfaction index-18 items version | Hospital anxiety and depression scale (HADS) | Activity limitation: Vulnerable Elderly Survey (ADL & IADL) | NA | Retrospective cohort studies | > 70 | 77.60 | 884 |
|  | Shin & Sok, 2011 | Korea | Community-dwelling | The Standard Life Satisfaction Instrument developed by Yun (1982) | Revised of the Korean Simple Depression Scale for the older people | NA | NA | Cross-sectional | > 65 | NR | 300 |
|  | Tavares, 2022 | Portugal | Community-dwelling | Single-item measure “Are you satisfied with your life?” | Three questions of depression indicators | Limitation on general ability | NA | Cross-sectional | > 70 | NR | 3959 |
|  | Tümer et al., 2021 | Turkey | Old‐age home | Life Satisfaction Scale (LSS) | NA | Physical competence: sub-scale from APS | Social support: perceived social support sub-scale from APS, loneliness: LSFE | Cross-sectional | > 65 | NR | 291 |
|  | Wang et al., 2002 | Japan | Community-dwelling | Life satisfaction scale-K (LSIK) | NA | Barthel Index, physical functioning scale | NA | Cross-sectional | 65-86 | 72.90 | 142 |
|  | Wangliu, 2023 | China | Community-dwellling | Single item measure: "“How do you rate your life at present?” | NA | NA | Financial, caring, & emotional support questions | Follow-up survey | > 65 | 83.83 | 3142 |
|  | Windle & Woods, 2004 | United Kingdom | Community-dwelling | Life Satisfaction Index (Wood, Wylie & Sheafor 1969) | NA | The physical functioning scale of the Short Form 36 questionnaire (SF-36) | Loneliness: A seven-item scale (Wenger et al. 1996) | Cross-sectional | > 70 | 78.00 | 423 |

**Table S4. Information of the data extraction (Part 2)**

| **Study ID** | **Author (year)** | **Gender/sex** | **Relevant predictors** | | | **Relevant key finding(s)** | **Risk of bias** |
| --- | --- | --- | --- | --- | --- | --- | --- |
|  |  |  | **Mental health** | **Physical capability** | **Social support** |  |  |
|  | Abu-Bader et al., 2003 | 87 (88%) women & 12 (12%) men | NA | ADL, IADL, Mobility | Social support | Social support was significantly correlated with life satisfaction while ADL, IADL, and mobility did not. | Fair |
|  | Adams et al., 2016 | 163 (68.8%) female & 74 (31.2%) | Depression, anxiety | NA | Social support | Depressive symptoms, anxiety, and social support significantly predicted life satisfaction. | Good |
|  | Altay & Çalmaz, 2023 | 68.5% female & 31.5% male | NA | NA | Loneliness | Loneliness was found to have a weak and significant negative association with life satisfaction. | Fair |
|  | Anaby et al., 2011 | 130 (65%) female & 70 (35%) male | NA | Mobility | Social support | Mobility and social support were significantly associated with life satisfaction. | Fair |
|  | Aquino et al., 1996 | 60% female & 40% male | Depression | NA | Social support | Depression and social support were significantly associated with life satisfaction | Fair |
|  | Bai et al., 2018 | female (85%) male (15%) | NA | IADL | Loneliness | Functional health, measuring IADL, was not associated with life satisfaction. Loneliness significantly correlated with life satisfaction | Fair |
|  | Berg et al., 2006 | 203 (64%) women & 112 (36%) men | Depression | IADL, PADL | Social support | Depression, IADL, and PADL were significantly associated with life satisfaction in men and women whereas social support was only significantly correlated in women. | Good |
|  | Berg et al., 2009 | NR | Depression | NA | NA | Initial depressive symptoms were related to lower levels of life satisfaction. | Good |
|  | Berglund et al., 2016 | 66% female & 44% male | Depression | PADL, IADL | NA | Depression significantly contributed to frail older people’s life satisfaction while PADL and IADL were not. | Good |
|  | Bourque et al., 2007 | 531 (64%) women & 295 (36%) men | NA | Functional limitation | Social support | Functional limitation and social support were significantly associated with life satisfaction | Good |
|  | Bowling et al., 1989 | 596 (90%) female & 66 (10%) male | NA | Mobility | Loneliness | Mobility and loneliness were correlated with life satisfaction in a sample of over 85-year-olds | Fair |
|  | Bowling et al., 1993 | 493 (79%) female & 133 (21%) male | NA | ADL | NA | ADL and social support's item "have someone would help if needed" were significantly associated with life satisfaction while item "has someone would understand" was not statistically significant. | Fair |
|  | Bowling et al., 1996 | NR | NA | ADL | NA | Functional status explained the remainder of the explained variance of life satisfaction, although this was relatively little. | Fair |
|  | Celik et al., 2017 | 54.5% female & 45.5% male | Depression | ADL, IADL | NA | Depression and household activities functionality significantly predicted life satisfaction but not personal care competency. However, those three variables were correlated with life satisfaction. | Good |
|  | Chang et al., 2001 | 79 (64%) women & 44 (36%) men | NA | ADL | NA | No significant relationship between overall scores of life satisfaction and functional fitness was found. | Fair |
|  | Chen et al., 2021 | 1,077 (52.6%) female & 971 (47.4%) male | NA | Self-care ability (ADL, IADL) | NA | Self-care ability was significantly associated with life satisfaction. | Good |
|  | Cheng & Yan, 2021 | 17,663 (58.3) female & 12,654 (41.7) male | Anxiety | NA | Loneliness | Anxiety and loneliness were significantly associated with life satisfaction. | Good |
|  | Cheng et al., 2019 | 705 (57%) female & 534 (43%) male | NA | ADL, Bayer ADL | NA | ADL and Bayer ADL were significantly correlated with life satisfaction. | Fair |
|  | Chou and Chi, 1999 | 134 (51.5%) female & 126 (48.5%) | Depression | Functional impartment | NA | Depression and functional impartment were significantly correlated with life satisfaction. | Good |
|  | Cihlar et al., 2023 | NR | NA | NA | Social support | Social support considerably moderates the negative relationship between vulnerability and life satisfaction. | Good |
|  | Dezutter et al., 2013 | 61% women & 39% men | Depression | NA | NA | Depression was associated with life satisfaction in older persons. | Fair |
|  | Didino et al., 2018 | 324 (66%) female & 165 (44%) male | NA | NA | Social support | Receiving help and emotional support significantly predicted life satisfaction. | Fair |
|  | Dumitrache et al., 2015 | 62.1% women & 37.9% men | NA | NA | Social support | Emotional, affectionate, and tangible support significantly predicted life satisfaction in old age. | Fair |
|  | Dumitrache et al., 2016 | 62.1% women & 37.9% men | NA | NA | Social support | Social support significantly predicted life satisfaction in old age. | Fair |
|  | Dumitrache et al., 2018 | 62.1% women & 37.9% men | NA | NA | Social support | Social support was significantly correlated with life satisfaction in old age. | Fair |
|  | Dumitrache et al., 2019 | 62.1% female & 37.9% male | NA | NA | Social support | Social support significantly predicted life satisfaction in old age. | Fair |
|  | Enkvist et al., 2012 | 390 (61%) female & 247 (39%) male | Depression | NA | ADL, IADL | Depression, ADL, and IADL were associated with life satisfaction. | Fair |
|  | Erci et al., 2017 | 167 (52.20%) female & 153 (47.80%) male | NA | Self-care ability | NA | Self-care ability was significantly correlated with life satisfaction. | Fair |
|  | Etxeberria et al., 2018 | 60.8% female & 39.2% male | NA | BADL, IADL | Social support | BADL, IADL, and social support were not correlated with life satisfaction in the oldest old. | Fair |
|  | Evans, 2009 | 96 (68.57%) women & 44 (31.43%) | NA | NA | Social support | Subjective level of social support was a strong positive predictor of life satisfaction. | Fair |
|  | Fukuzawa & Sugawara, 2022 | 183 (44.9%) women & 225 (55.1) men | NA | NA | Social support, loneliness | There was a significant correlation between loneliness with life satisfaction (LS) while social support was not significantly correlated. | Good |
|  | Grenier et al., 2018 | 689 (58%) women & 504 (42%) men | Anxiety | NA | NA | Significant differences were found on life satisfaction between older patients with GAD and those without anxiety disorder. | Fair |
|  | Got et al., 2023 | NR | Depression | IADL, BADL | NA | IADL significantly predicted life satisfaction in non-dementia participants while depression significantly predicted life satisfaction in non-dementia and dementia participants | Good |
|  | Hajek & Konig, 2021 | 424 (44.5%) women & 528 (55.5%) men | Depression | IADL | Loneliness | Depression, IADL, and loneliness were significantly correlated with life satisfaction of the very old in North Rhine‐Westphalia. | Fair |
|  | Harasemiw et al., 2019 | 53.30% female & 46.70% male | Depression | NA | affectionate, emotional, positive social interaction, & tangible support | Depression was moderately corelated with LS. Among all the relevant social support sub-scales, tangible support did not predict life satisfaction in married older adults while in unmarried group, emotional support that did not significantly predicted life satisfaction. | Good |
|  | Helvik et al., 2011 | 5,911 (54%) & 5,047 (46%) male | Depression, anxiety | NA | NA | Depression and anxiety had a strong impact on satisfaction with life. | Good |
|  | Heo et al., 2015 | NR | NA | NA | Loneliness | Loneliness was found to have significant effect on life satisfaction. | Fair |
|  | Huang & Fu, 2021 | NR | NA | ADL | Financial, instrumental and emotional support | ADL, Financial, instrumental and emotional support significantly predicted the life satisfaction. | Good |
|  | Jung et al., 2010 | 2,023 (61.7) women & 1,255 (38.3) men | NA | ADL | NA | ADL in male group did not significantly predict the life satisfaction while in female group was significant. | Good |
|  | Kaucic et al., 2019 | 470 (71.6%) female & 186 (28.4) male | NA | The functional ability | NA | The functional ability did not significantly contribute to life satisfaction. | Fair |
|  | Kim & Sok, 2013 | 243 (100%) women | Depression | NA | NA | Depression was significantly correlated with life satisfaction in older women who living alone. | Good |
|  | Ko & Jung, 2021 | 6,035 (59.9%) women & 4046 (40.1%) men | Depression | NA | NA | Depression was significantly correlated with life satisfaction in Community-Dwelling Older Koreans. | Good |
|  | Koong et al., 2023 | 5937 (59.9%) feamle & 3696 (40.1%) female | Depression | IADL | NA | Increasing dependence on others for IADL and depression were negatively correlated with life satisfaction. | Good |
|  | Krause, 2004 | 42% men & 58% women | NA | NA | Emotional support | Older adults who receive more emotional support from significant others tend to be more satisfied with their lives than older people who do not get much emotional support | Fair |
|  | Kwon et al., 2023 | NR | Depression | ADL | NA | ADL and depression correlated significantly with life satisfaction | Fair |
|  | Lai et al., 2023 | 56 (83.6%) female & 11 (16.4%) male | Anxiety | NA | NA | A higher level of life satisfaction was associated with less anxiety symptoms | Good |
|  | Lalani et al., 2023 | 801 (59.3%) female & 550 (40.7%) male | NA | IADL | NA | Older people with more IADL limitations reported lower life satisfaction | Good |
|  | Lara et al., 2020 | 77 (50%) female & 77 (50%) male | NA | NA | Social support | Social support significantly associated with life satisfaction. | Good |
|  | Lee & Lee, 2013 | 2417 (58%) women & 1735 (42%) men | Depression | NA | NA | Depression significantly predicted life satisfaction in both groups (less educated & better educated participants) | Good |
|  | Lee et al., 2014 | 59% female & 41 male | NA | ADL, IADL | NA | Any ADL/IADL limitation significantly predicted life satisfaction. | Good |
|  | Lee & Williams, 2023 | 202 (37%) female & 343 (63%) male | Depression | NA | NA | Depression was positively associated with life satisfaction among older adults | Good |
|  | Liu et al., 2016 | 343 (77.6%) female & 99 (22.4%) male | NA | ADL | NA | ADL (dependent) significantly was correlated with life satisfaction of the individuals over 95 years old. | Good |
|  | Liu et al., 2020 | 112 (49.1%) female & 116 (50.9%) male | NA | Physical function-ADL | NA | Functional capacity was significantly correlated with life satisfaction in older people living in long-term care facility | Good |
|  | Lu & Chang, 1997 | 91 males (52.9%) & 81 females (47.1%) | NA | Daily activities | Social support | Daily activities and social support significantly associated with Life satisfaction. | Fair |
|  | Lu et al., 2020 | 68.3% female & 31.7% male | NA | Functional health | NA | The functional health significantly predicted life satisfaction. | Good |
|  | Luo et al., 2023 | 41 (47%) female & 58 (53%) male | NA | Physical functioning | NA | Physical functioning did not correlate significantly with life satisfaction | Good |
|  | Mellor & Edelmann, 1988 | 31 (86%) female & 5 (14%) male | NA | Mobility | loneliness | Mobility and loneliness in older adults significantly correlated with life satisfaction. | Fair |
|  | Newsom & Schulz, 1996 | 57% women & 43% men | Depression | Physical impartment | Belonging support, appraisal support, tangible support | Depression, physical impartment, and social support were significantly correlated with life satisfaction (LS). | Good |
|  | Ni Mhaolain et al., 2011 | 55.4% female and 44.6% male | Depression | IADL | Loneliness | Depression, IADL, and loneliness significantly contributed to life satisfaction. | Good |
|  | Nilsson et al., 2007 | 108 (69%) women & 48 (31%) men | NA | ADL | NA | Older people with lower ADL had statistically lower life satisfaction than did those with higher ADL. | Fair |
|  | Noh et al., 2017 | 2,380 (57.6%) female & 1,754 (42.4%) male | Depression | ADL | NA | Depression and activity daily living significantly affected life satisfaction. | Fair |
|  | Onal et al., 2022 | 346 (50.4%) females & 340 (49.6%) males | NA | NA | Social support, loneliness | Perceived social support and loneliness were significantly correlated with older adults' life satisfaction during the COVID-19 pandemic. | Good |
|  | Park, 2024 | 1708 (68%) female & 830 (32%) male | NA | NA | Emotional, informational, instrumental social support | Emotional, informational, instrumental social support correlated significantly with life satisfaction | Good |
|  | Park & Sok, 2020 | 154 (67.2%) females & males 75 (32.8%) | Depression, anxiety | NA | NA | Depression and anxiety significantly affected life satisfaction of Korean Older Adults in Long-Term Care Facilities. | Good |
|  | Perkins et al., 2007 | 100% women | NA | Physical impartment | Social support | Physical impartment was significantly associated with life satisfaction whereas social support did not. | Good |
|  | Pinto et al., 2016 | 65.7% women | Depression | NA | NA | Depression had a significant association with on life satisfaction. | Good |
|  | Pynnönen et al., 2021 | 585 (57.3%) female & 436 (42.7) male | Depression | Physical performance | Loneliness | Physical performance and loneliness significantly contributed to life satisfaction. | Good |
|  | Rochelle, 2022 | 153 (76.5%) female & 47 (23.5%) male | NA | NA | Loneliness | Loneliness was found to have significant association with life satisfaction. | Fair |
|  | Roh et al., 2015 | 85 (42.5%) female & 115 (57.5%) male | Depression | NA | NA | Depression was significantly associated with life satisfaction on older Korean American | Good |
|  | Sahin et al., 2019 | 246 (47.6%) female & 271 (52.4%) male | NA | NA | Social support | Social support was significantly associated with life satisfaction. | Good |
|  | Schilling et al., 2013 | 354 (78.5%) female & 97 (21.5%) male | NA | ADL | NA | The ADL was significantly associated with life satisfaction in advanced old age. | Good |
|  | Sexton et al., 2017 | 474 (53.6%) female & 410 (46.4%) male | Depression, anxiety | Activity limitation (ADL & IADL) | NA | Activity limitations, anxiety, and depression had significant association on life satisfaction. | Good |
|  | Shin & Sok, 2011 | 185 (61,7%) female & 115 (38,3%) male | Depression | NA | NA | Depression was found to be one of the factors related to the life satisfaction of older people living with their family and those living alone. | Fair |
|  | Tavares, 2022 | 61.7% female & 38.3 male | Depression | Physical limitation | NA | Depression and physical limitation are significant drivers of life satisfaction | Fair |
|  | Tümer et al., 2021 | 156 (53.6%) female & 135 (46.4%) male | NA | Physical competence | Physical competence, social support, loneliness | Physical competence, social support, and loneliness significantly correlated with life satisfaction. | Fair |
|  | Wang et al., 2002 | 86 (61%) female & 56 (39%) male | NA | ADL, physical functioning | NA | ADL and physical functioning were significantly associated with life satisfaction in female participants. | Fair |
|  | Wangliu, 2023 | "42.3% | NA | NA | Financial, caring, & emotional support questions | Life satisfaction mediated the relationship between emotional support and social participation | Good |
|  | Windle & Woods, 2004 | NR | NA | Physical function | Loneliness | Physical function and loneliness correlated significantly with life satisfaction. | Good |

**Supplementary E. Quality assessments**

**Table S5. Quality Assessments for each included study**

| **Study ID** | **Author, year** | Was the research question or objective in this paper clearly stated? | Was the study population clearly specified and defined? | Was the participation rate of eligible persons at least 50%? | Were all the subjects selected or recruited from the same or similar populations (including the same time period)? Were inclusion and exclusion criteria for being in the study prespecified and applied uniformly to all participants? | Was a sample size justification, power description, or variance and effect estimates provided? | For the analyses in this paper, were the exposure(s) of interest measured prior to the outcome(s) being measured? | Was the timeframe sufficient so that one could reasonably expect to see an association between exposure and outcome if it existed? | For exposures that can vary in amount or level, did the study examine different levels of the exposure as related to the outcome (e.g., categories of exposure, or exposure measured as continuous variable)? | Were the exposure measures (independent variables) clearly defined, valid, reliable, and implemented consistently across all study participants? | Was the exposure(s) assessed more than once over time? | Were the outcome measures (dependent variables) clearly defined, valid, reliable, and implemented consistently across all study participants? | Were the outcome assessors blinded to the exposure status of participants? | Was loss to follow-up after baseline 20% or less? | Were key potential confounding variables measured and adjusted statistically for their impact on the relationship between exposure(s) and outcome(s)? | | **Summary of the quality** |
| --- | --- | --- | --- | --- | --- | --- | --- | --- | --- | --- | --- | --- | --- | --- | --- | --- | --- |
|  | Abu-Bader et al., 2003 | Yes | Yes | Yes | Yes | Yes | NA | NA | Yes | No | NA | Yes | NA | NA | Yes | Fair | |
|  | Adams et al., 2016 | Yes | Yes | Yes | Yes | Yes | NA | NA | Yes | Yes | NA | Yes | NA | NA | Yes | Fair | |
|  | Altay & Çalmaz, 2023 | Yes | Yes | NR | Yes | Yes | NA | NA | Yes | Yes | NA | Yes | NA | NA | No | Fair | |
|  | Anaby et al., 2011 | Yes | Yes | NR | Yes | No | NA | NA | Yes | Yes | NA | Yes | NA | NA | Yes | Fair | |
|  | Aquino et al., 1996 | Yes | No | NR | Yes | No | NA | NA | Yes | Yes | NA | Yes | NA | NA | Yes | Fair | |
|  | Bai et al., 2018 | Yes | Yes | NR | Yes | No | NA | NA | Yes | Yes | NA | Yes | NA | NA | Yes | Fair | |
|  | Berg et al., 2006 | Yes | Yes | Yes | Yes | Yes | NA | NA | Yes | Yes | NA | Yes | NA | NA | Yes | Fair | |
|  | Berg et al., 2009 | Yes | Yes | Yes | Yes | Yes | Yes | Yes | Yes | Yes | Yes | Yes | NA | No | Yes | Fair | |
|  | Berglund et al., 2016 | Yes | Yes | Yes | Yes | Yes | NA | NA | Yes | Yes | NA | Yes | NA | NA | Yes | Fair | |
|  | Bourque et al., 2007 | Yes | Yes | Yes | Yes | Yes | NA | NA | Yes | Yes | NA | Yes | NA | NA | Yes | Fair | |
|  | Bowling et al., 1989 | Yes | Yes | No | Yes | No | NA | NA | Yes | Yes | NA | Yes | NA | NA | No | Fair | |
|  | Bowling et al., 1993 | Yes | Yes | NR | Yes | No | NA | Yes | Yes | Yes | Yes | Yes | NA | No | Yes | Fair | |
|  | Bowling et al., 1996 | Yes | Yes | NR | Yes | No | NA | Yes | Yes | Yes | Yes | Yes | NA | No | Yes | Fair | |
|  | Celik et al., 2017 | Yes | Yes | Yes | Yes | Yes | NA | NA | Yes | Yes | NA | Yes | NA | NA | Yes | Fair | |
|  | Chang et al., 2001 | Yes | Yes | NR | Yes | No | NA | NA | Yes | Yes | NA | Yes | NA | NA | No | Fair | |
|  | Chen et al., 2021 | Yes | Yes | Yes | Yes | Yes | NA | NA | Yes | Yes | NA | Yes | NA | NA | Yes | Fair | |
|  | Cheng & Yan, 2021 | Yes | Yes | Yes | Yes | Yes |  | NA | Yes | Yes | NA | Yes | NA | NA | Yes | Fair | |
|  | Cheng et al., 2019 | Yes | Yes | NR | No | No | NA | NA | Yes | Yes | NA | Yes | NA | NA | Yes | Fair | |
|  | Chou and Chi, 1999 | Yes | Yes | Yes | Yes | Yes | NA | Yes | Yes | Yes | No | Yes | NA | No | Yes | Fair | |
|  | Cihlar et al., 2023 | Yes | Yes | Yes | Yes | No | NA | NA | Yes | Yes | NA | Yes | NA | NA | Yes | Good | |
|  | Dezutter et al., 2013 | Yes | Yes | NR | Yes | No | NA | NA | Yes | Yes | NA | Yes | NA | No | Yes | Fair | |
|  | Didino et al., 2018 | Yes | Yes | NR | Yes | No | NA | NA | Yes | Yes | NA | Yes | NA | NA | Yes | Fair | |
|  | Dumitrache et al., 2015 | Yes | Yes | NR | Yes | Yes | NA | NA | Yes | Yes | NA | Yes | NA | NA | Yes | Fair | |
|  | Dumitrache et al., 2016 | Yes | Yes | NR | Yes | Yes | NA | NA | Yes | Yes | NA | Yes | NA | NA | Yes | Fair | |
|  | Dumitrache et al., 2018 | Yes | Yes | NR | Yes | Yes | NA | NA | Yes | Yes | NA | Yes | NA | NA | Yes | Fair | |
|  | Dumitrache et al., 2019 | Yes | Yes | NR | Yes | Yes | NA | NA | Yes | Yes | NA | Yes | NA | NA | Yes | Fair | |
|  | Enkvist et al., 2012 | Yes | No | NR | Yes | No | NA | Yes | Yes | Yes | No | Yes | NA | No | Yes | Fair | |
|  | Erci et al., 2017 | Yes | Yes | NR | Yes | Yes | NA | NA | Yes | Yes | NA | Yes | NA | NA | No | Fair | |
|  | Etxeberria et al., 2018 | Yes | No | NR | Yes | No | NA | NA | Yes | Yes | NA | Yes | NA | NA | Yes | Fair | |
|  | Evans, Ronnie J., 2019 | Yes | Yes | NR | Yes | No | NA | NA | Yes | Yes | NA | Yes | NA | NA | Yes | Fair | |
|  | Fukuzawa & Sugawara, 2022 | Yes | Yes | NR | Yes | No | NA | NA | Yes | Yes | NA | Yes | NA | NA | Yes | Fair | |
|  | Gotanda et al., 2023 | Yes | Yes | Yes | Yes | Yes | NA | NA | Yes | Yes | NA | Yes | NA | NA | Yes | Good | |
|  | Grenier et al., 2018 | Yes | Yes | Yes | Yes | Yes | NA | NA | Yes | Yes | NA | Yes | NA | NA | Yes | Good | |
|  | Hajek & Konig, 2021 | Yes | Yes | No | Yes | Yes | NA | NA | Yes | Yes | NA | Yes | NA | NA | Yes | Good | |
|  | Harasemiw et al., 2019 | Yes | Yes | Yes | Yes | Yes | NA | NA | Yes | Yes | NA | Yes | NA | NA | Yes | Good | |
|  | Helvik et al., 2011 | Yes | Yes | Yes | Yes | Yes | NA | NA | Yes | Yes | NA | Yes | NA | NA | Yes | Good | |
|  | Heo et al., 2015 | Yes | Yes | NR | Yes | No | NA | NA | Yes | Yes | NA | Yes | NA | NA | Yes | Good | |
|  | Huang & Fu, 2021 | Yes | Yes | NR | Yes | No | NA | NA | Yes | Yes | NA | Yes | NA | NA | Yes | Good | |
|  | Jung et al., 2010 | Yes | Yes | No | Yes | Yes | NA | NA | Yes | Yes | NA | Yes | NA | NA | Yes | Good | |
|  | Kaucic et al., 2019 | Yes | Yes | NR | Yes | Yes | NA | NA | Yes | Yes | NA | Yes | NA | NA | Yes | Good | |
|  | Kim & Sok, 2013 | Yes | Yes | NR | Yes | Yes | NA | NA | Yes | Yes | NA | Yes | NA | NA | Yes | Good | |
|  | Ko & Jung, 2021 | Yes | Yes | Yes | Yes | Yes | NA | NA | Yes | Yes | NA | Yes | NA | NA | Yes | Good | |
|  | Koong et al., 2023 | Yes | Yes | Yes | Yes | Yes | NA | NA | Yes | Yes | NA | Yes | NA | NA | Yes | Good | |
|  | Krause, 2004 | Yes | Yes | NR | Yes | No | NA | Yes | Yes | Yes | NR | Yes | NA | No | Yes | Good | |
|  | Kwon et al., 2023 | Yes | Yes | NR | Yes | No | NA | NA | Yes | Yes | NA | Yes | NA | NA | Yes | Fair | |
|  | Lai et al., 2023 | Yes | Yes | NR | Yes | Yes | NA | NA | Yes | Yes | NA | Yes | NA | NA | Yes | Good | |
|  | Lalani et al., 2023 | Yes | Yes | Yes | Yes | Yes | NA | NA | Yes | Yes | NA | Yes | NA | NA | Yes | Good | |
|  | Lara et al., 2020 | Yes | Yes | NR | Yes | Yes | NA | NA | Yes | Yes | NA | Yes | NA | NA | Yes | Good | |
|  | Lee & Lee, 2013 | Yes | Yes | Yes | Yes | NR | NA | NA | Yes | Yes | NA | Yes | NA | NA | Yes | Good | |
|  | Lee & Williams, 2023 | Yes | Yes | Yes | Yes | Yes | NA | NA | Yes | Yes | NA | Yes | NA | NA | Yes | Good | |
|  | Lee et al., 2014 | Yes | Yes | NR | Yes | No | NA | NA | Yes | Yes | NA | Yes | NA | NA | Yes | Good | |
|  | Liu et al., 2016 | Yes | Yes | Yes | Yes | No | NA | NA | Yes | Yes | NA | Yes | NA | NA | Yes | Good | |
|  | Liu et al., 2020 | Yes | Yes | Yes | Yes | Yes | NA | NA | Yes | Yes | NA | Yes | NA | NA | Yes | Good | |
|  | Lu & Chang, 1997 | Yes | Yes | NR | No | No | NA | NA | Yes | Yes | NA | Yes | NA | NA | Yes | Good | |
|  | Lu et al., 2020 | Yes | Yes | Yes | Yes | Yes | NA | NA | Yes | Yes | NA | Yes | NA | NA | Yes | Good | |
|  | Luo et al., 2023 | Yes | Yes | Yes | Yes | Yes | NA | NA | Yes | Yes | NA | Yes | NA | NA | Yes | Good | |
|  | Mellor & Edelmann, 1988 | Yes | Yes | NR | No | No | NA | NA | Yes | Yes | NA | Yes | NA | NA | No | Good | |
|  | Newsom & Schulz, 1996 | Yes | Yes | Yes | Yes | Yes | NA | NA | Yes | Yes | NA | Yes | NA | NA | Yes | Good | |
|  | Ni Mhaolain et al., 2011 | Yes | Yes | No | Yes | Yes | NA | NA | Yes | Yes | NA | Yes | NA | NA | Yes | Good | |
|  | Nilsson et al., 2007 | Yes | Yes | Yes | Yes | Yes | NA | NA | Yes | Yes | NA | Yes | NA | NA | No | Good | |
|  | Noh et al., 2017 | Yes | Yes | Yes | Yes | No | NA | NA | Yes | Yes | NA | Yes | NA | NA | Yes | Good | |
|  | Onal et al., 2022 | Yes | Yes | NR | Yes | Yes | NA | NA | Yes | Yes | NA | Yes | NA | NA | Yes | Good | |
|  | Park & Sok, 2020 | Yes | Yes | NR | Yes | Yes | NA | NA | Yes | Yes | NA | Yes | NA | NA | Yes | Good | |
|  | Park, 2024 | Yes | Yes | Yes | Yes | Yes | NA | NA | Yes | Yes | NA | Yes | NA | NA | Yes | Good | |
|  | Perkins et al., 2007 | Yes | Yes | No | Yes | Yes | NA | NA | Yes | Yes | NA | Yes | NA | NA | Yes | Good | |
|  | Pinto et al., 2016 | Yes | Yes | NR | Yes | Yes | NA | NA | Yes | Yes | NA | Yes | NA | NA | Yes | Good | |
|  | Pynnönen et al., 2021 | Yes | Yes | NR | Yes | Yes | NA | NA | Yes | Yes | NA | Yes | NA | NA | No | Good | |
|  | Rochelle, 2022 | Yes | Yes | No | Yes | Yes | NA | NA | Yes | Yes | NA | Yes | NA | NA | Yes | Good | |
|  | Roh et al., 2015 | Yes | Yes | Yes | Yes | No | NA | NA | Yes | Yes | NA | Yes | NA | NA | Yes | Good | |
|  | Sahin et al., 2019 | Yes | Yes | NR | Yes | Yes | NA | NA | Yes | Yes | NA | Yes | NA | NA | Yes | Good | |
|  | Schilling et al., 2013 | Yes | Yes | NR | Yes | Yes | NA | Yes | Yes | Yes | Yes | Yes | NA | No | Yes | Good | |
|  | Sexton et al., 2017 | Yes | Yes | Yes | Yes | Yes | NA | NA | Yes | Yes | NA | Yes | NA | NA | Yes | Good | |
|  | Shin & Sok, 2011 | Yes | Yes | NR | Yes | Yes | NA | NA | Yes | Yes | NA | Yes | NA | NA | Yes | Good | |
|  | Tavares, 2022 | Yes | Yes | Yes | Yes | Yes | NA | NA | Yes | No | NA | Yes | NA | NA | Yes | Fair | |
|  | Tümer et al., 2021 | Yes | Yes | Yes | Yes | Yes | NA | NA | Yes | Yes | NA | Yes | NA | NA | No | Fair | |
|  | Wang et al., 2002 | Yes | Yes | NR | Yes | No | NA | NA | Yes | Yes | NA | Yes | NA | NA | No | Fair | |
|  | Wangliu, 2023 | Yes | Yes | Yes | Yes | Yes | NA | NA | Yes | Yes | NA | Yes | NA | NA | Yes | Good | |
|  | Windle & Woods, 2004 | Yes | Yes | Yes | Yes | Yes | NA | NA | Yes | Yes | NA | Yes | NA | NA | Yes | Good | |

**Supplementary F. Sensitivity analysis**

1. **Physical capability and life satisfaction**


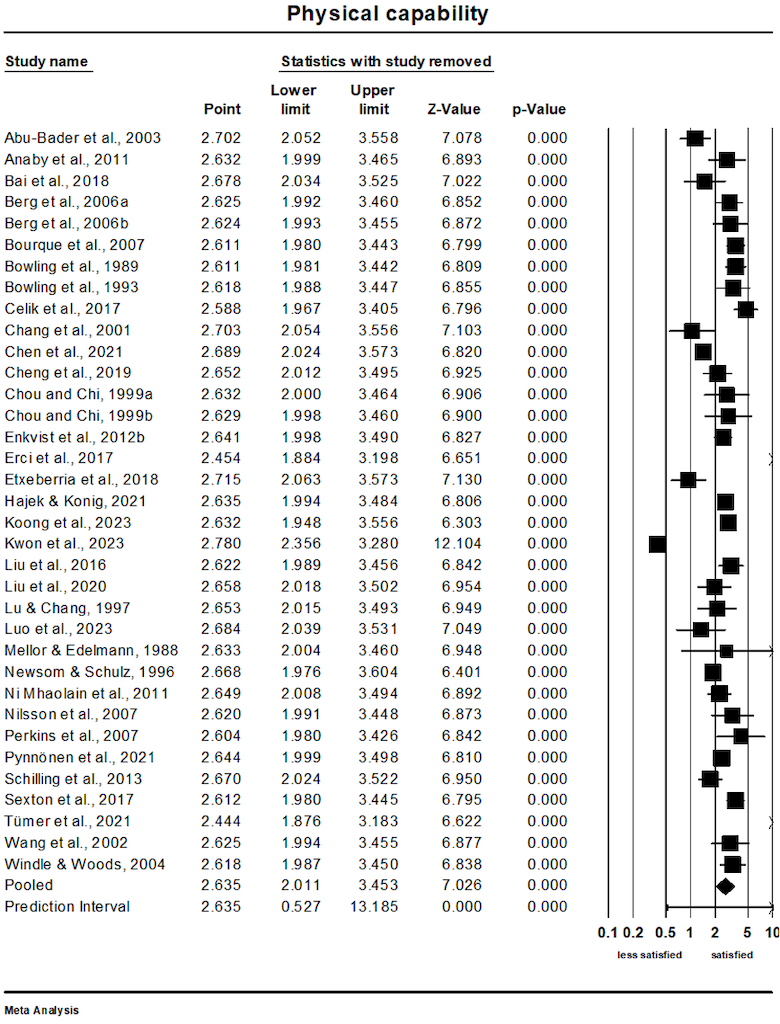


Figure S 1. Physical capability and life satisfaction: one study removed


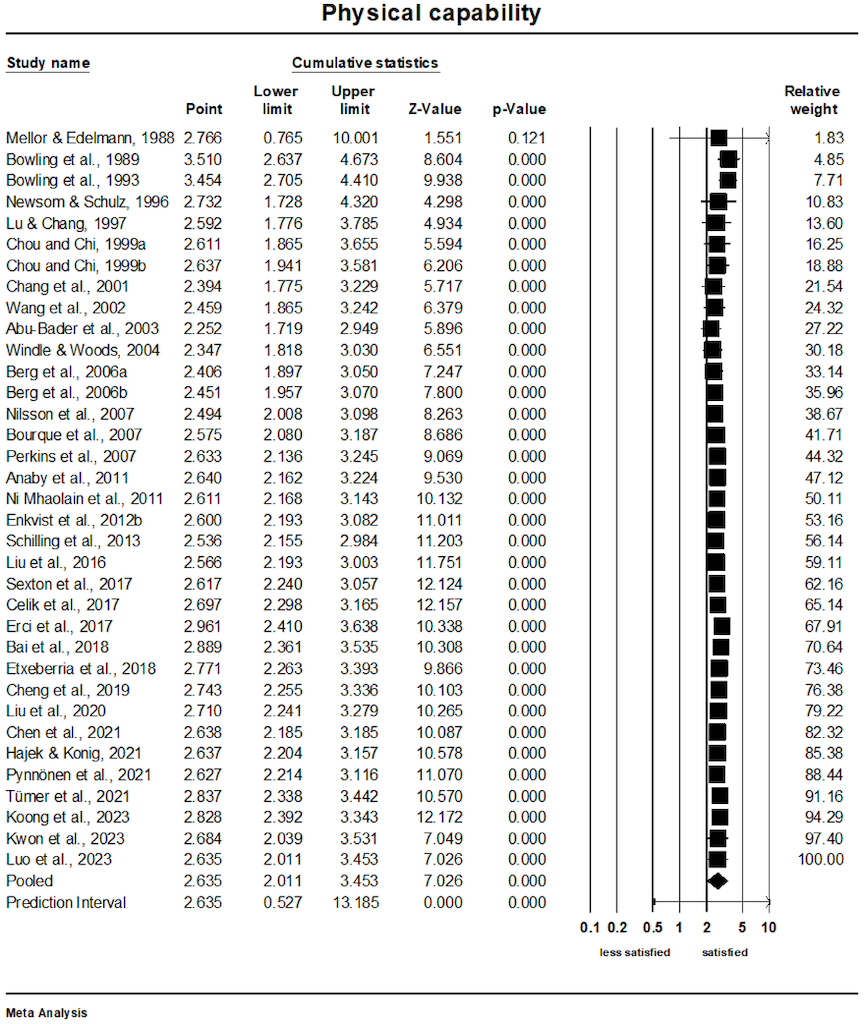


Figure S 2. Physical capability and life satisfaction: cumulative analysis

**II. Social support and life satisfaction**


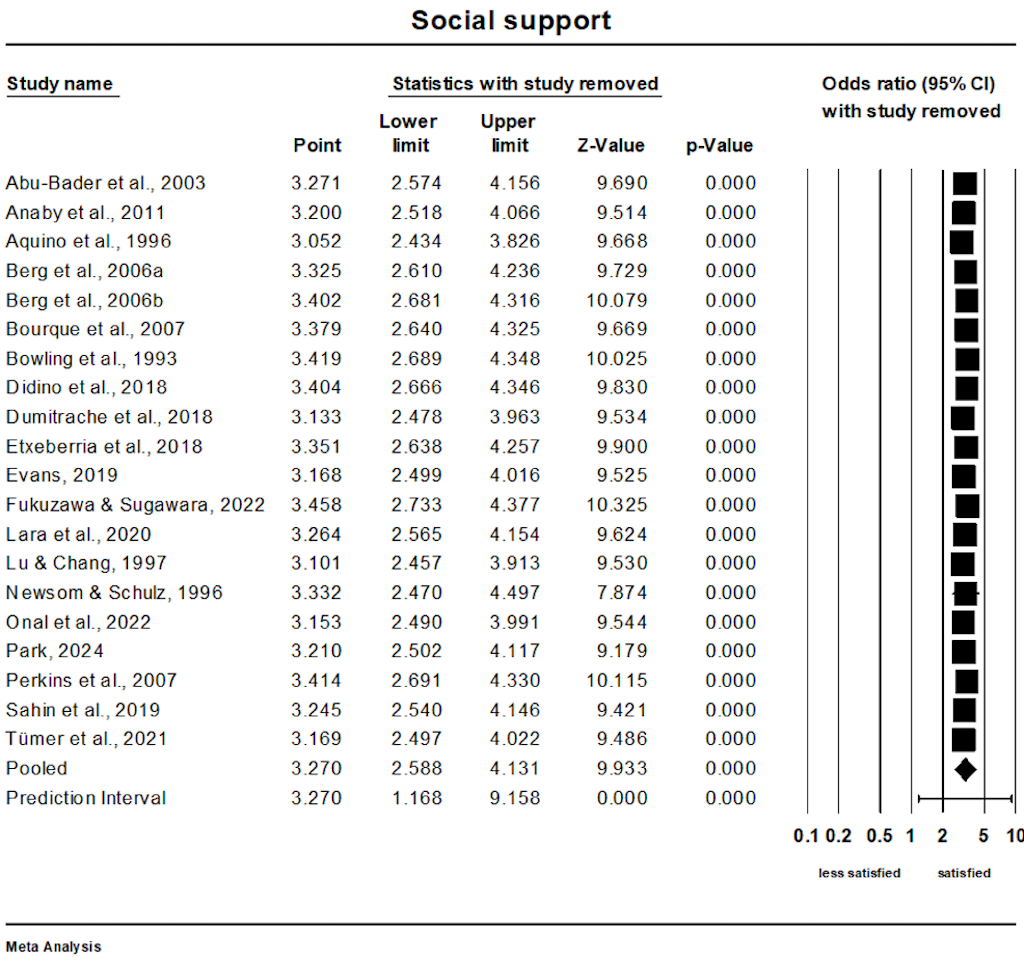


Figure S 3. Social support and life satisfaction: one study removed


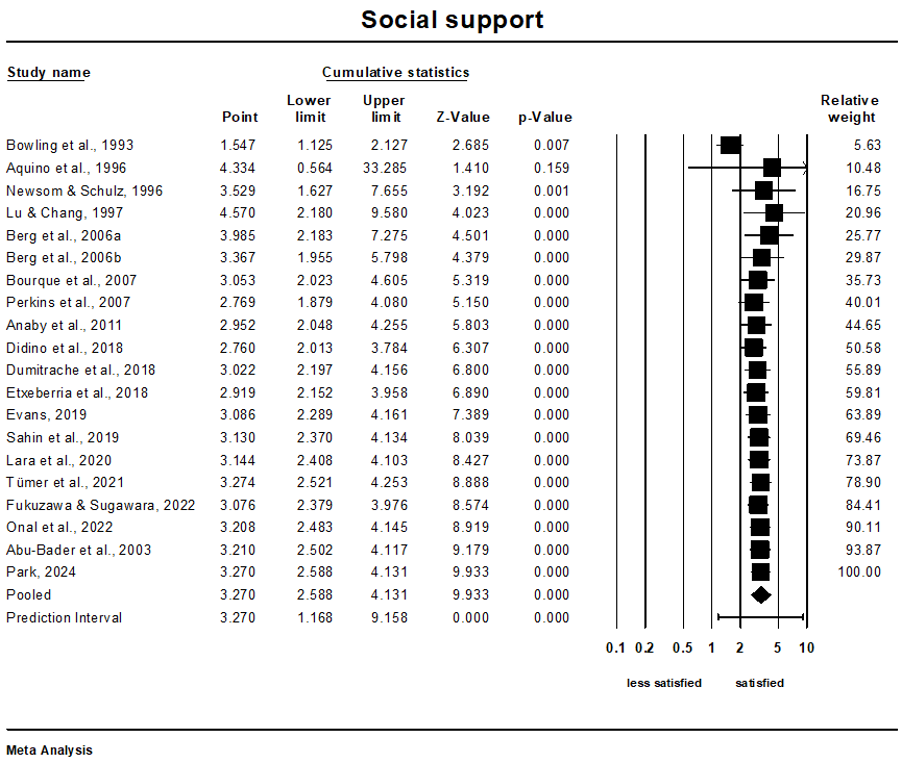


Figure S 4. Social support and life satisfaction: cumulative analysis

1. **Loneliness and life satisfaction**


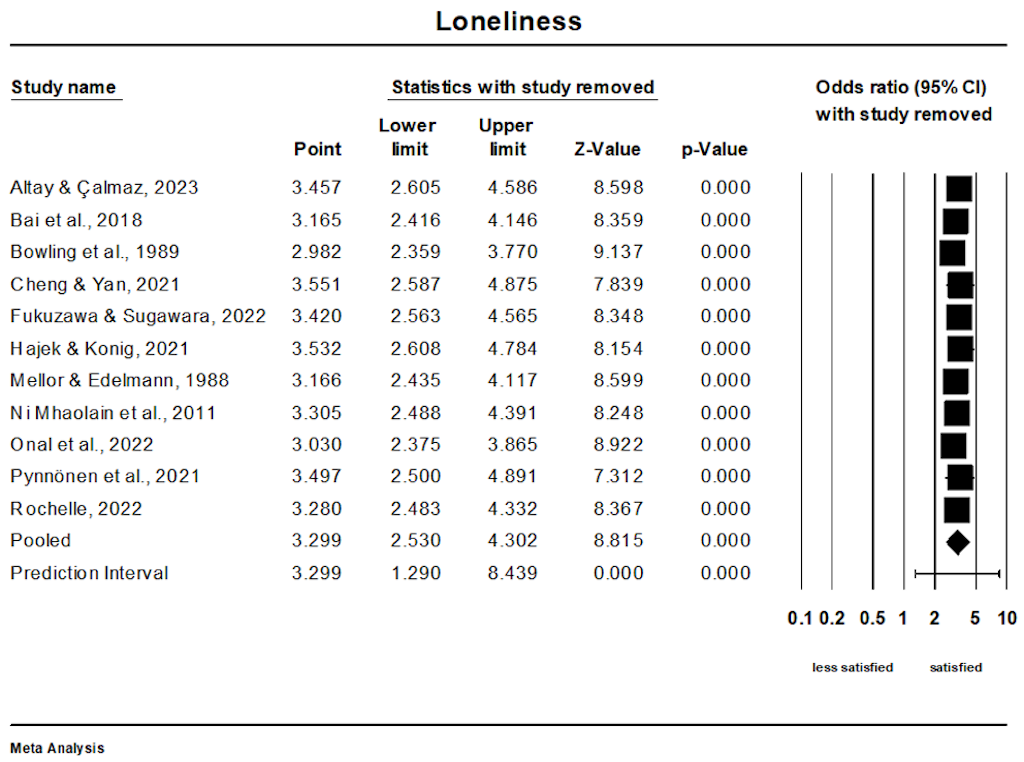


Figure S 5. Loneliness and life satisfaction: one study removed


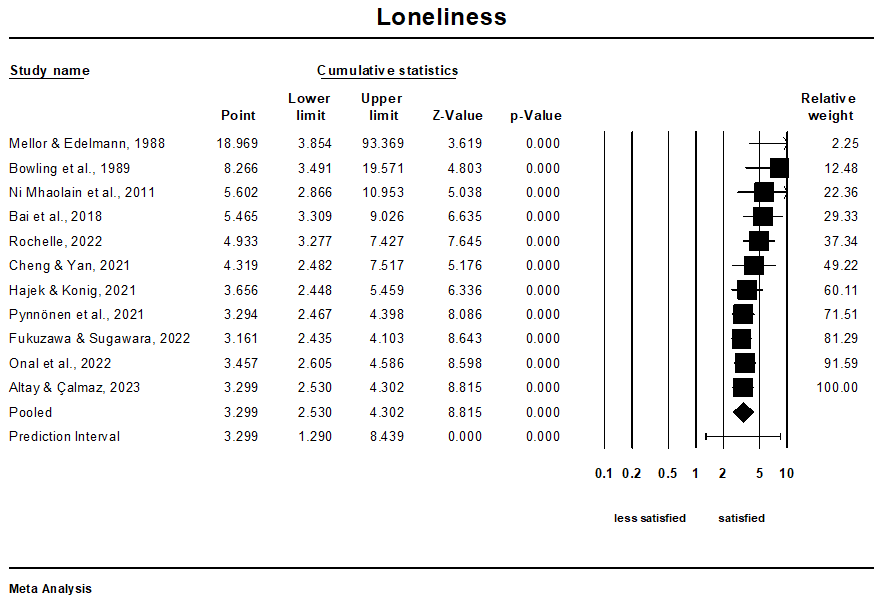


Figure S 6. Loneliness and life satisfaction: cumulative analysis

1. **Depression and life satisfaction**


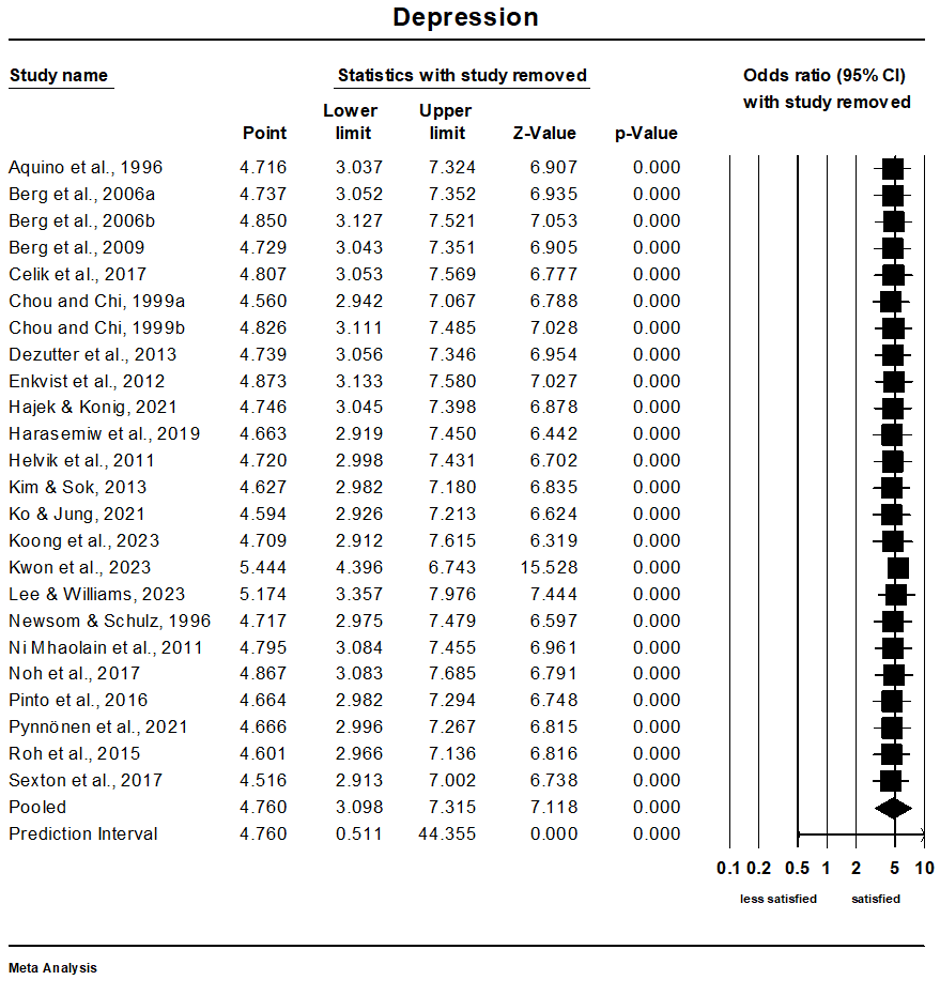


Figure S 7. Depression and life satisfaction: one study removed


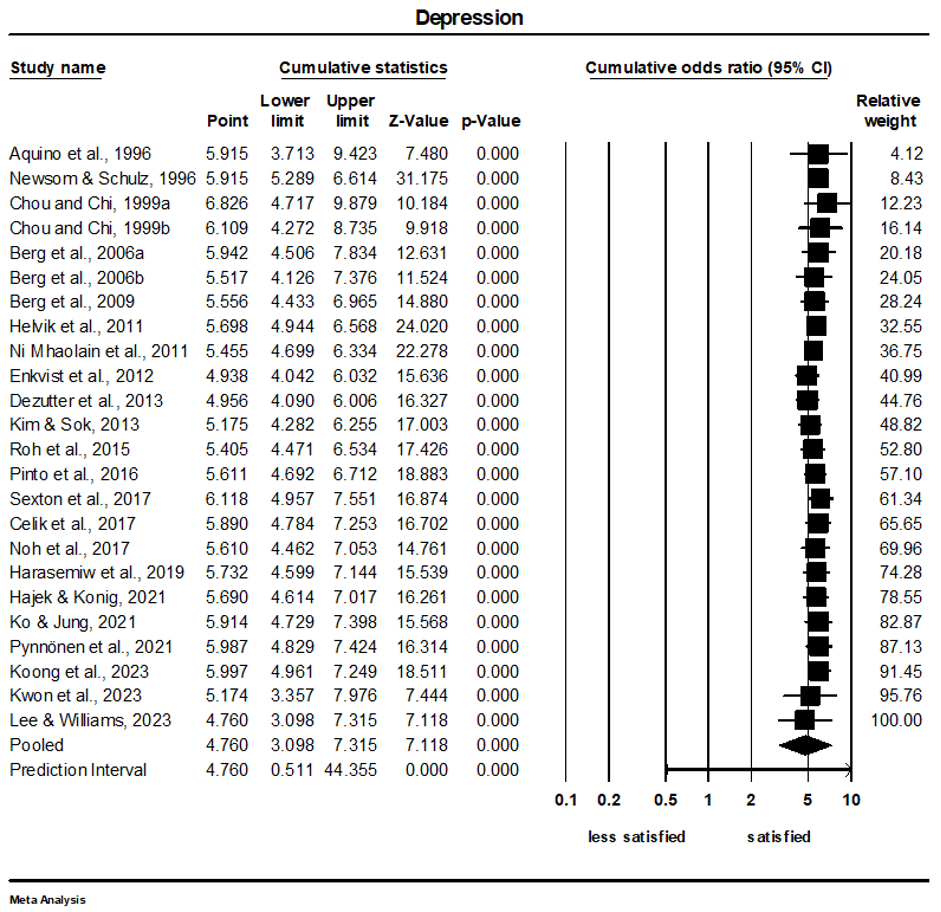


Figure S 8. Depression and life satisfaction: cumulative analysis

1. **Anxiety and life satisfaction**


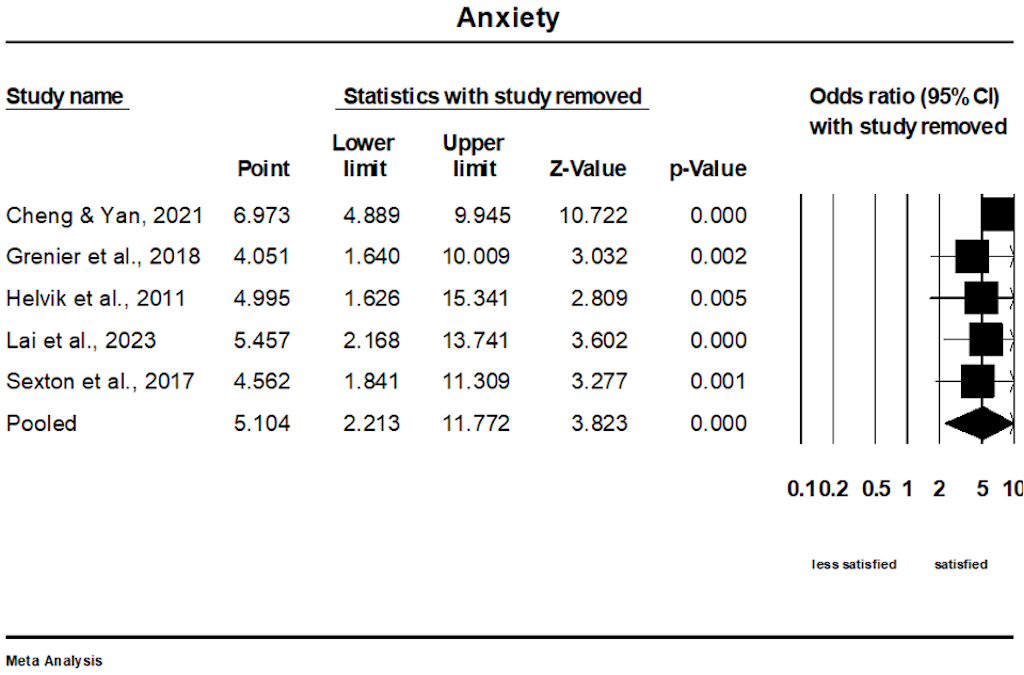


Figure S 9. Anxiety and life satisfaction: one study removed

Figure S 10. Anxiety and life satisfaction: cumulative anal
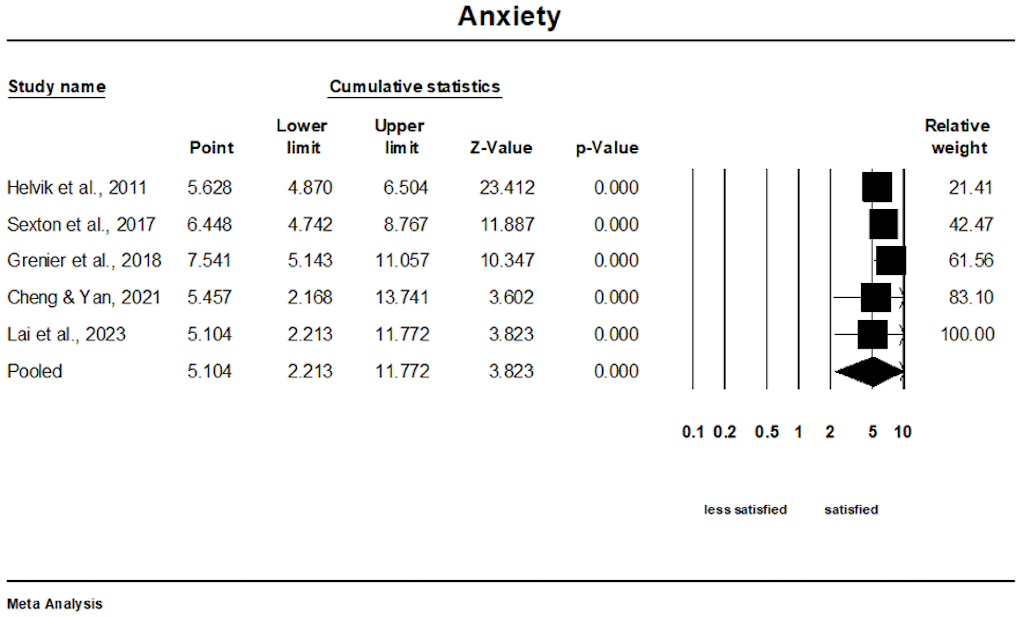
ysis

**Supplementary G. The standardized mean difference**

**Table S6. Outcome (life satisfaction) analysed using SMD**

| **Variable** | **No. of study** | **SMD** | **95% CI [95% PI]** |
| --- | --- | --- | --- |
| Physical capabilities | 35 | 0.53** | 0.39-0.69 |
| Social support | 20 | 0.65** | 0.09-1.22 |
| Loneliness | 11 | 0.66** | 0.51-0.80 |
| Depression | 24 | 0.86** | 0.62-1.10 |
| Anxiety | 5 | 0.90** | 0.44-1.36 |

**** p<0.001**


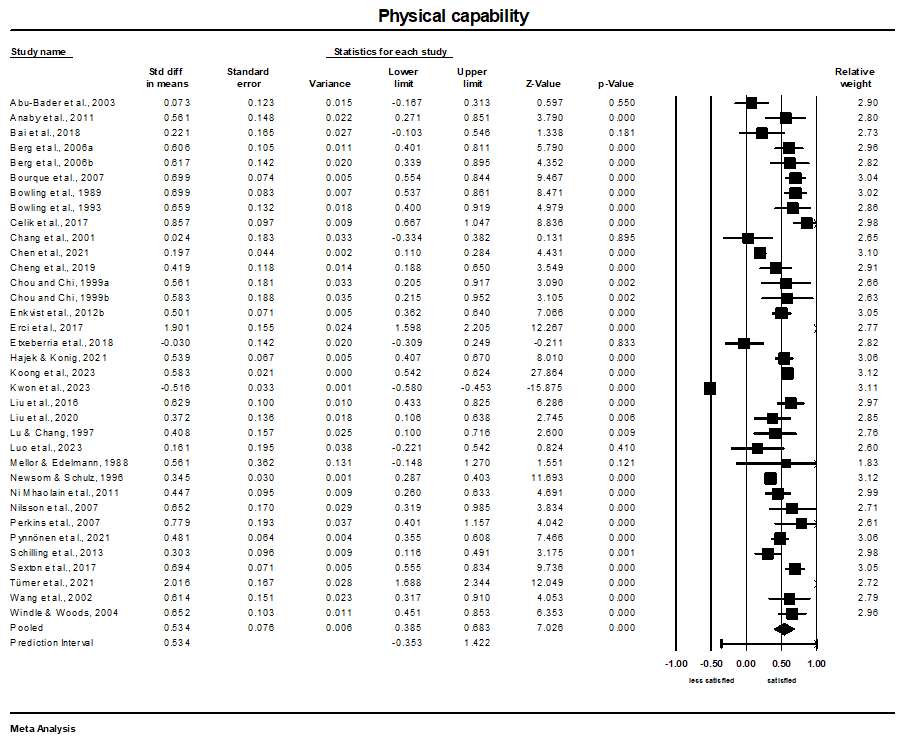


Figure S 11. The standardized mean difference of the association of physical capability and life satisfaction


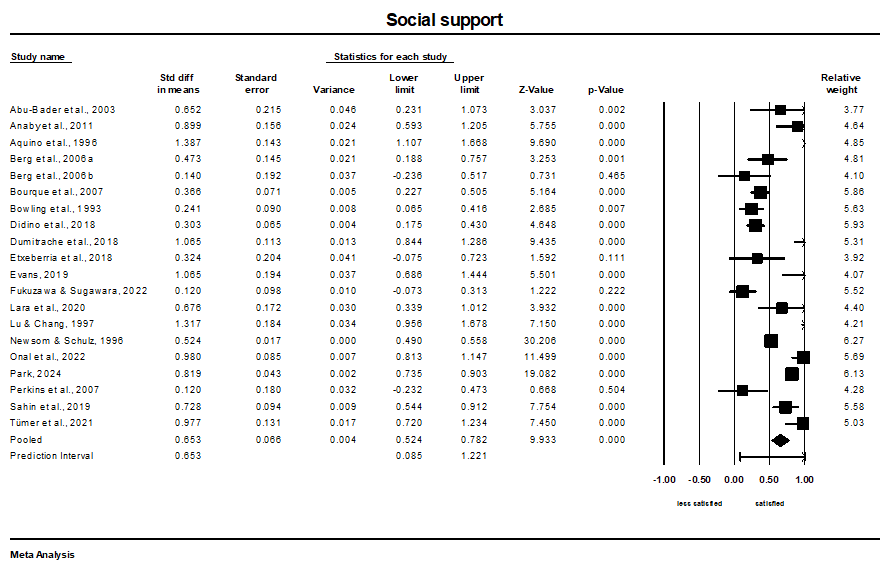


Figure S 12. The standardized mean difference of the association of social support and life satisfaction


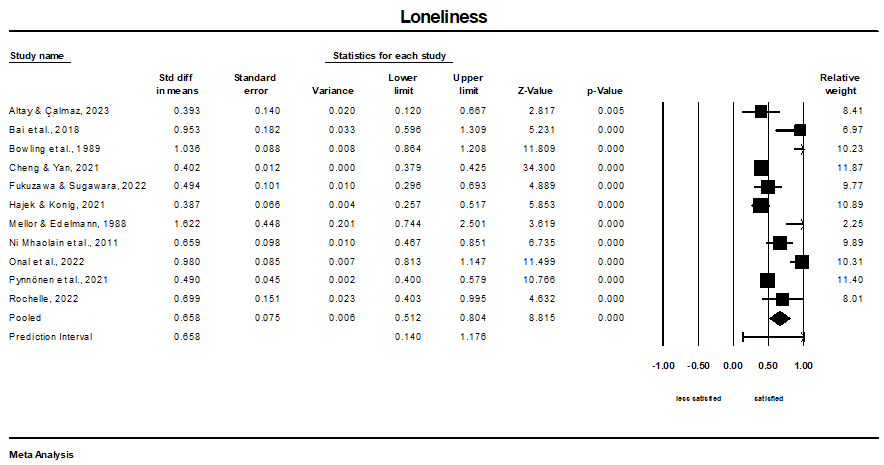


Figure S 13. The standardized mean difference of the association of loneliness and life satisfaction


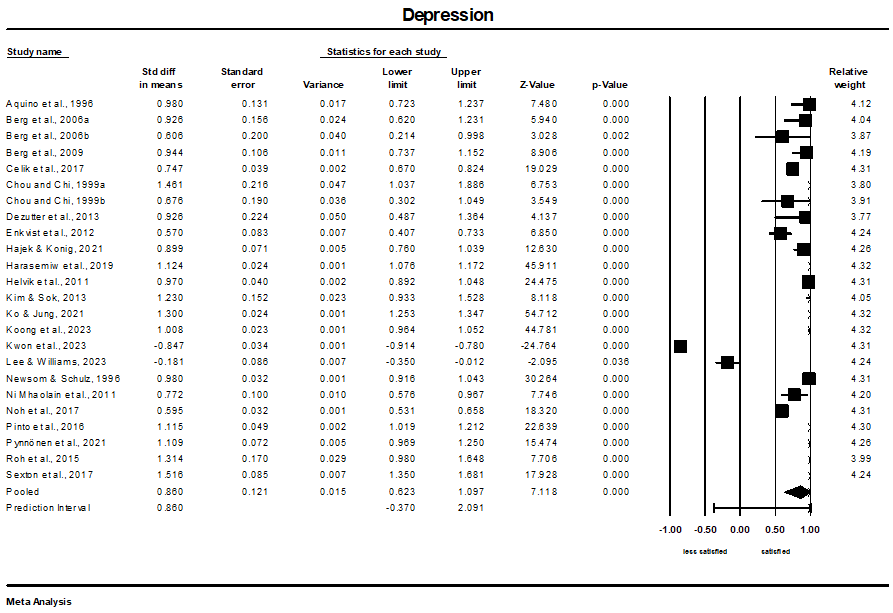


Figure S 14. The standardized mean difference of the association of depression and life satisfaction


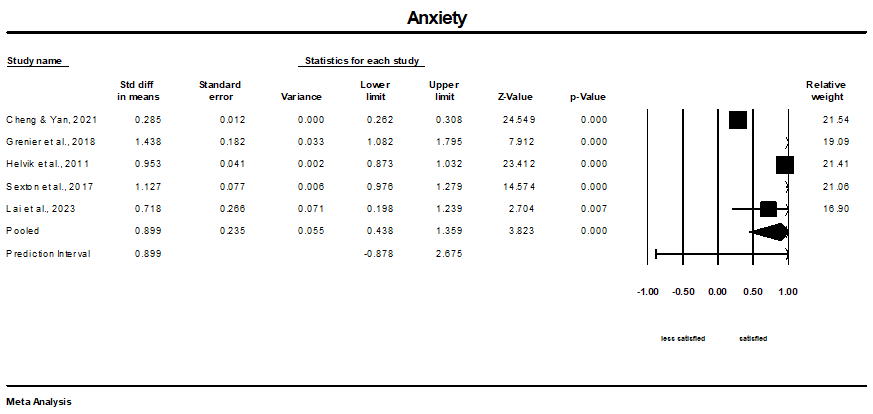


Figure S 15. The standardized mean difference of the association of anxiety and life satisfaction

**Supplementary H. Sub-group analysis (Western vs Eastern)**


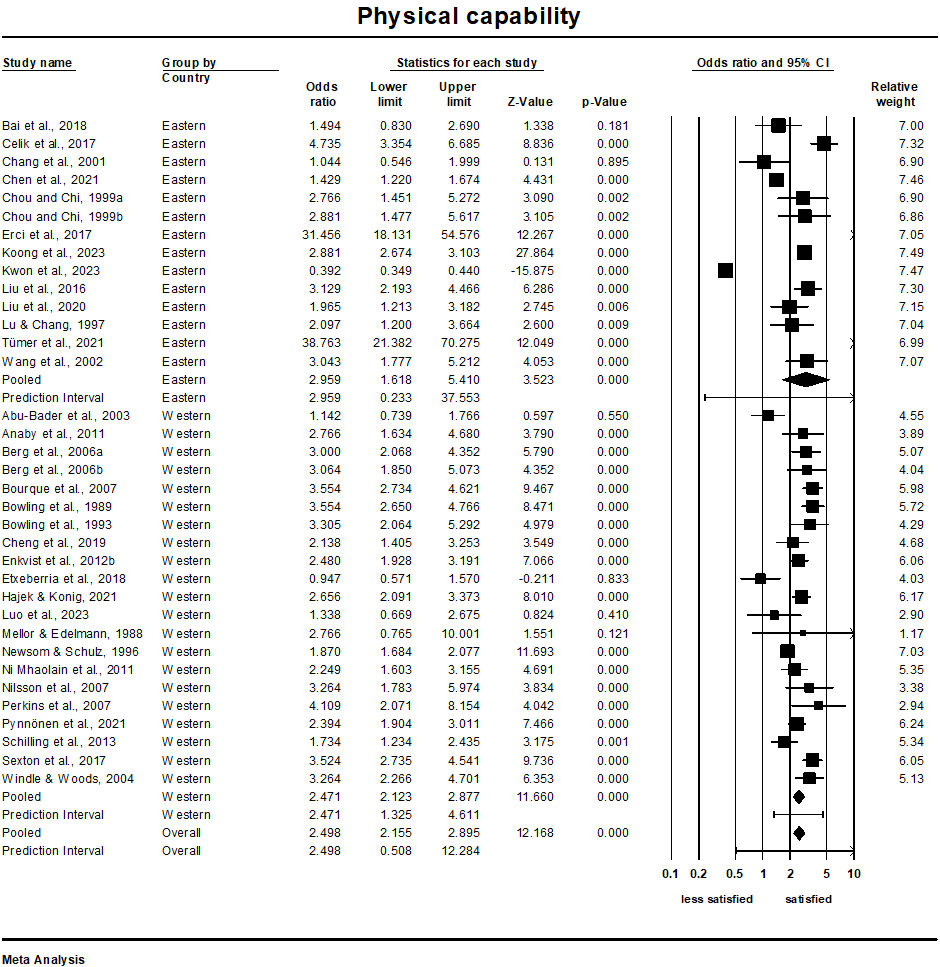


Figure S 16. Forest plot of the association of physical capability and life satisfaction, grouped by country


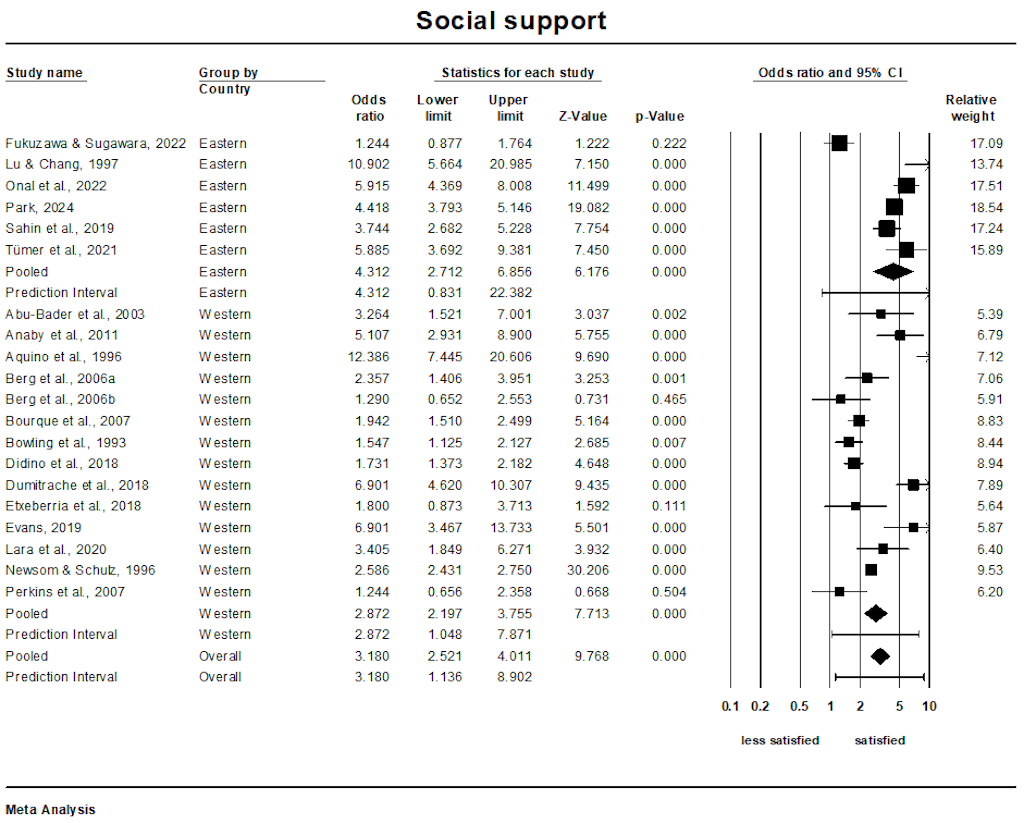


Figure S 17. Forest plot of the association of social support and life satisfaction, grouped by country


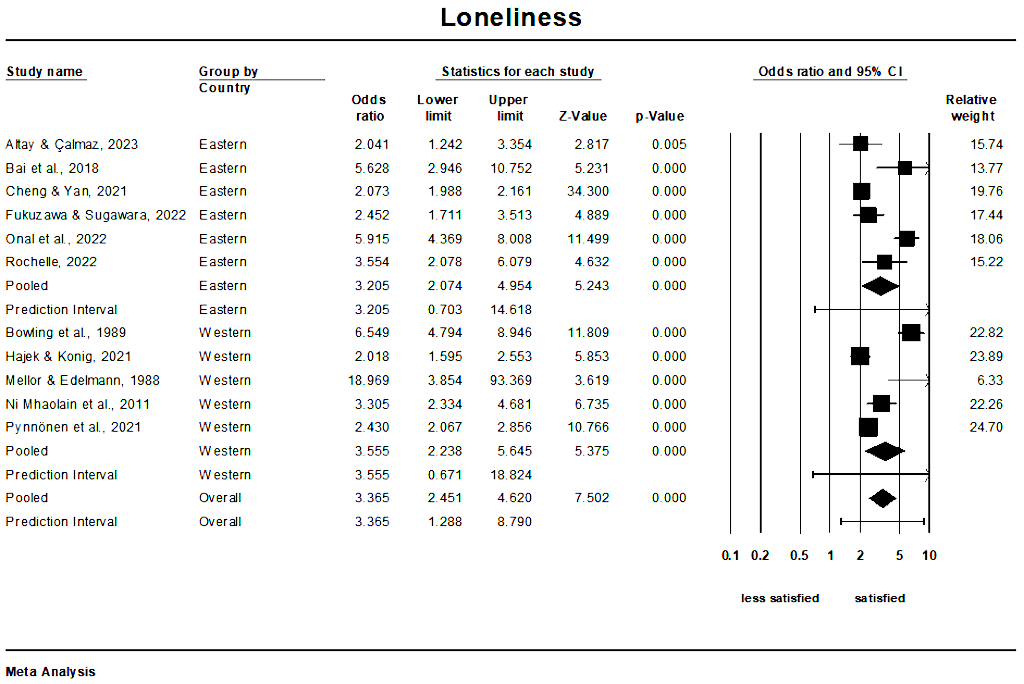


Figure S 18. Forest plot of the association of loneliness and life satisfaction, grouped by country


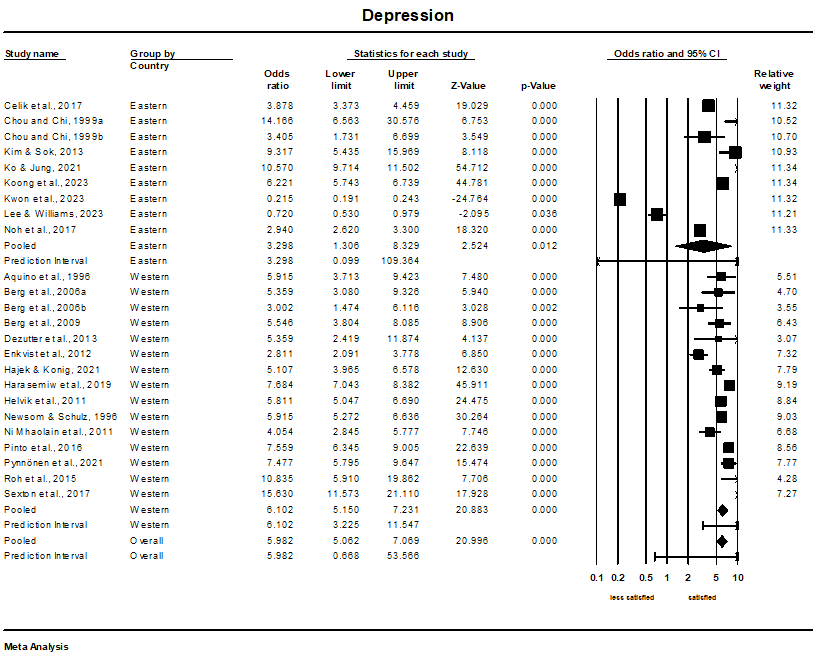


Figure S 19. Forest plot of the association of depression and life satisfaction, grouped by country

**Supplementary I. Sub-group analysis (Quality assessment: Good vs Fair)**


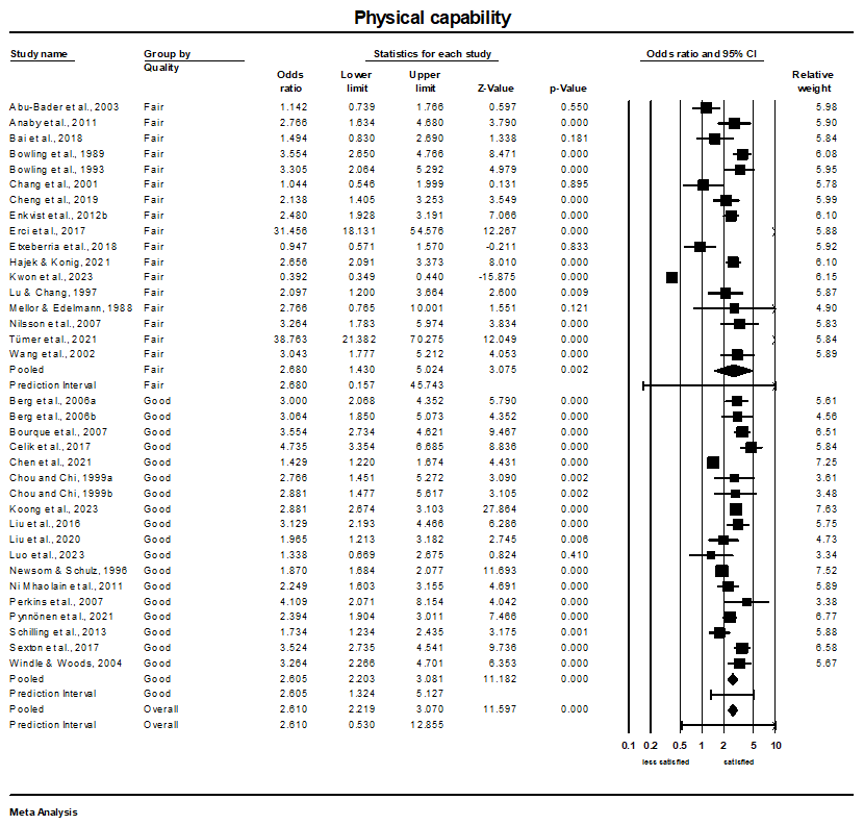


Figure S 20. Forest plot of the association of physical capability and life satisfaction, grouped by quality assessment


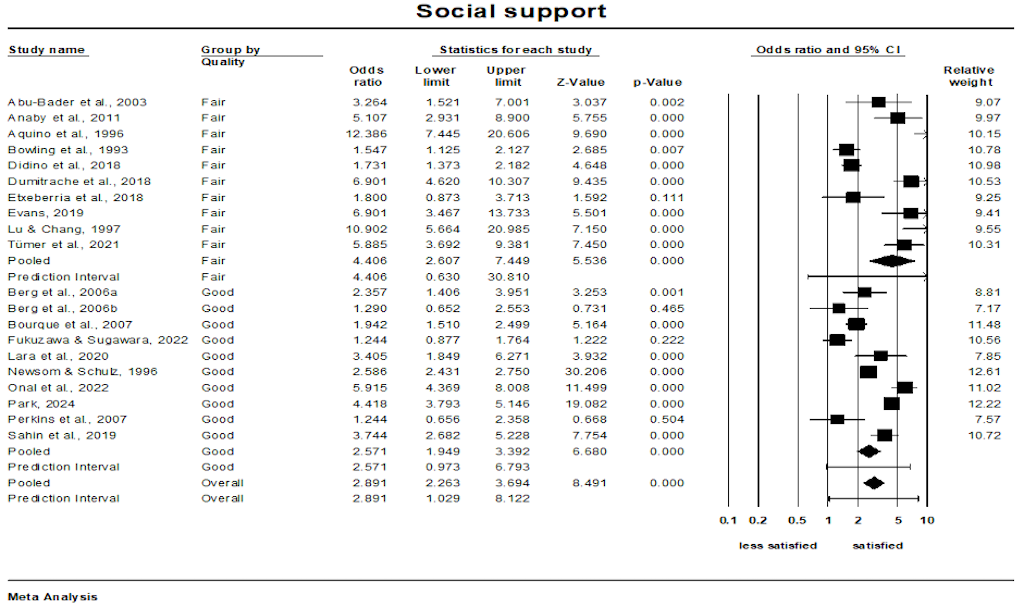


Figure S 21. Forest plot of the association of social support and life satisfaction, grouped by quality assessment


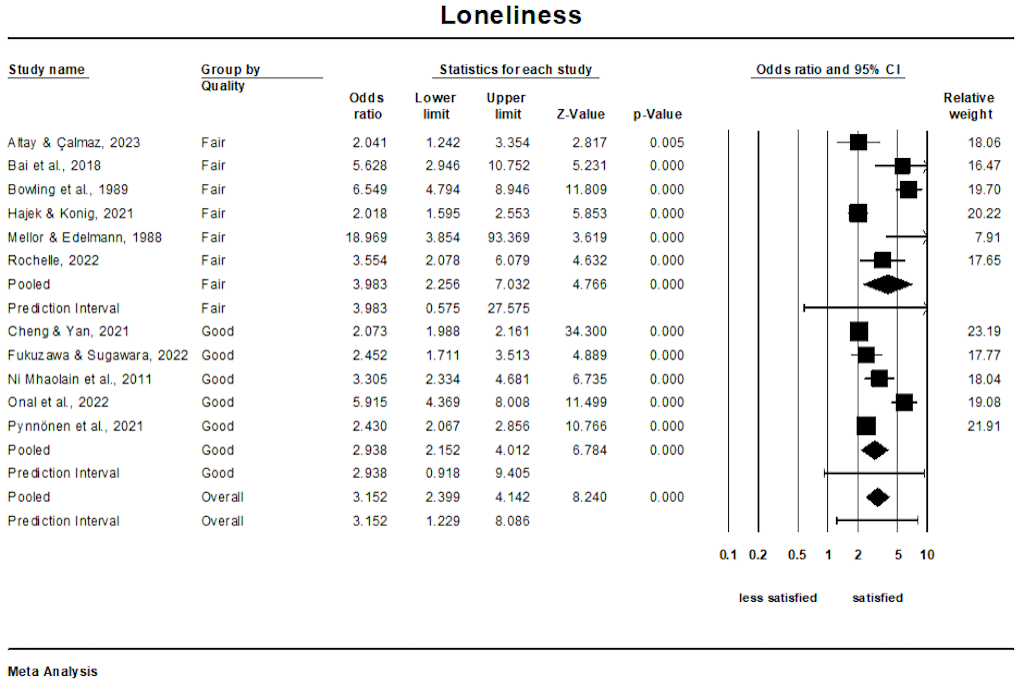


Figure S 22. Forest plot of the association of loneliness and life satisfaction, grouped by quality assessment


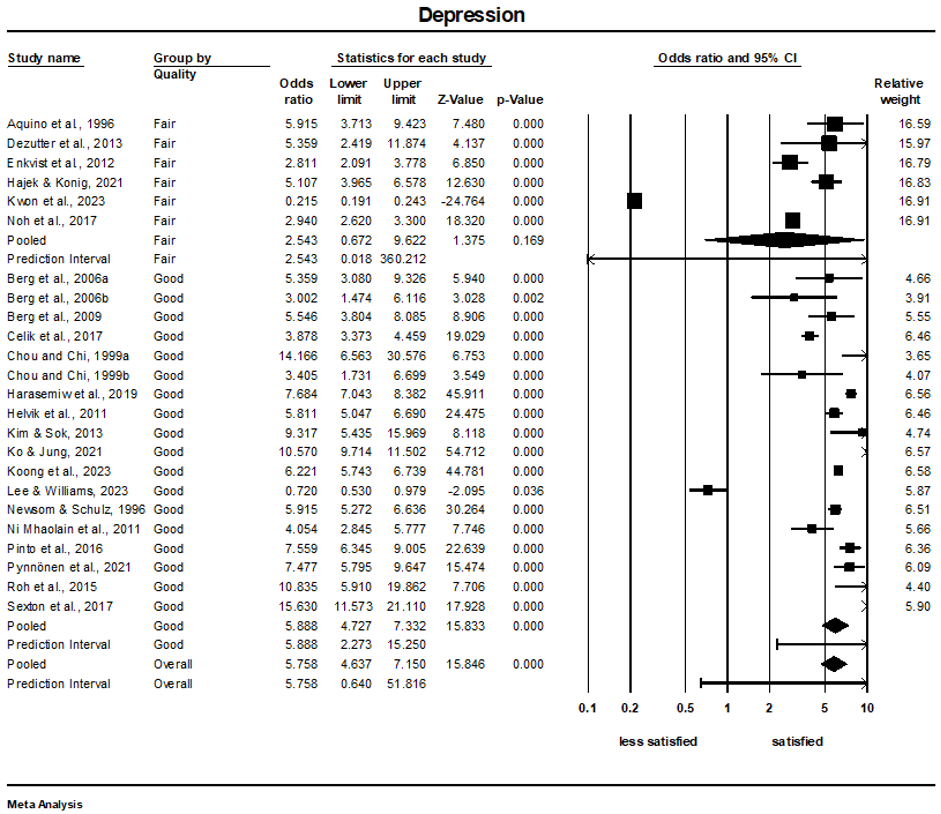


Figure S 23. Forest plot of the association of depression and life satisfaction, grouped by quality assessment

**Supplementary J. Scatterplot of the meta-regression**

1. **Scatterplot of the meta-regression of mean age for the association of social support and life satisfaction**


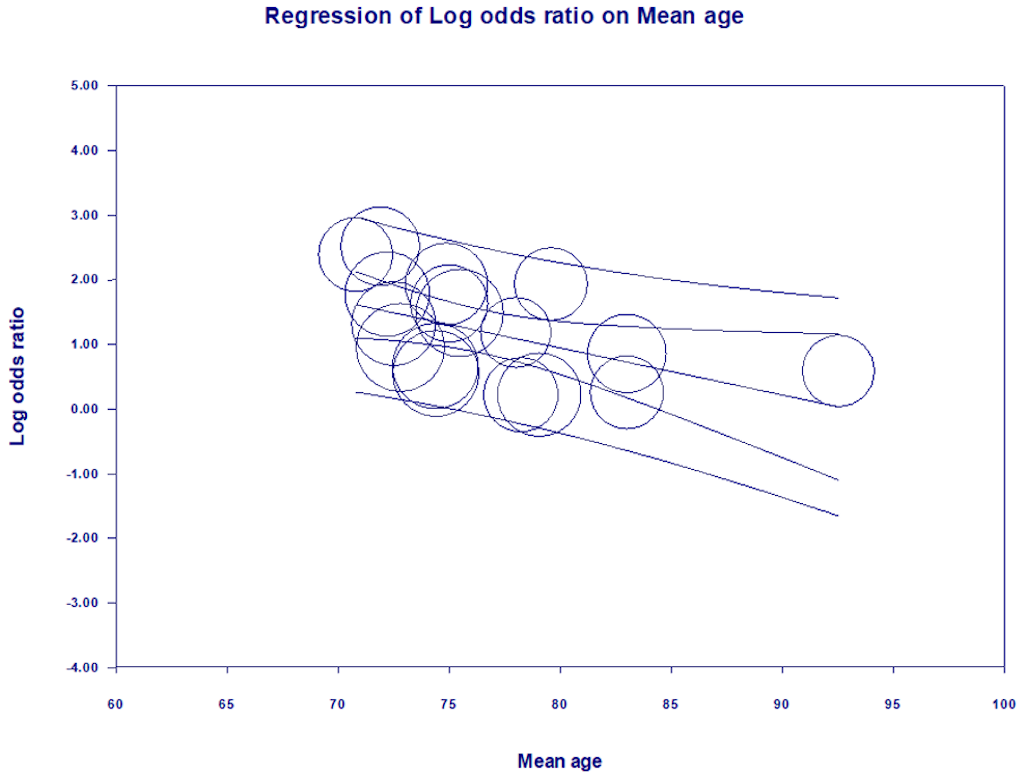


Figure S 24.Scatterplot of the meta-regression of mean age of participants which moderated the association between social support and life satisfaction

1. **Scatterplot of the meta-regression of proportion of female participants for the association of loneliness and life satisfaction**


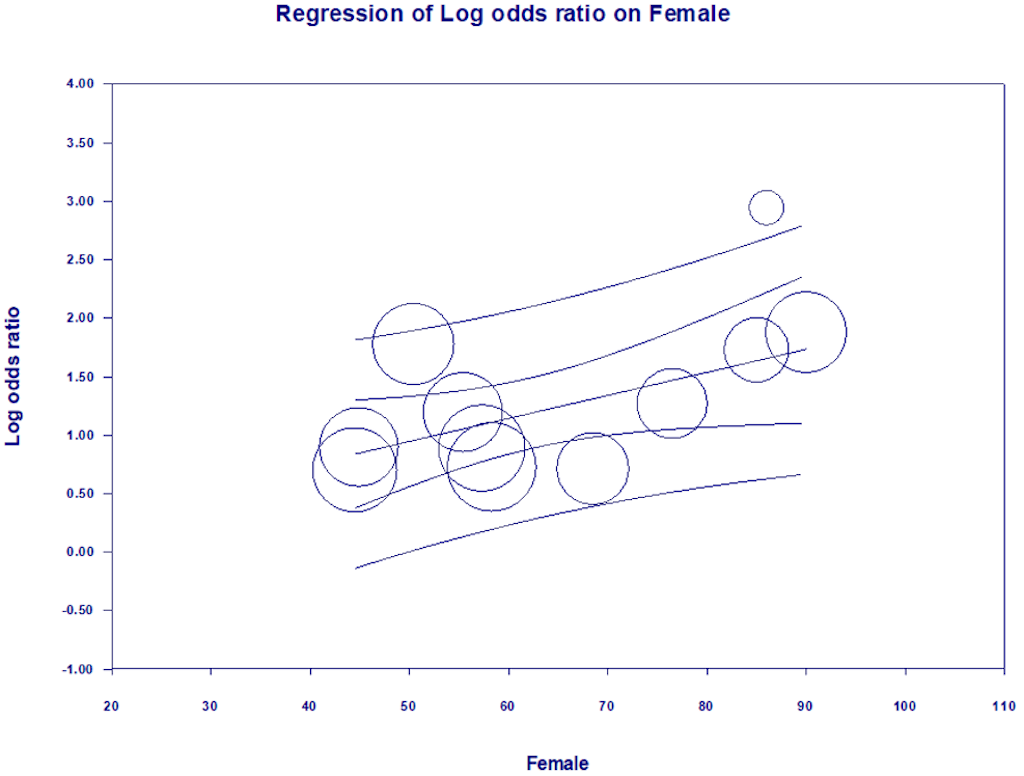


Figure S 25. Scatterplot of the meta-regression of the proportion of female participants which moderated the association between loneliness and life satisfaction

**Supplementary K. Publication bias**

*Publication bias studies of physical capability and life satisfaction*

Publication bias assessment for the physical capability: evident from the analysis of the funnel plot, which reveals a predominantly symmetrical distribution. A funnel plot with symmetrically distributed combined effect size indicated the absence of publication bias. If bias existed, the lower portion of the plot would exhibit a greater density of studies on one side of the mean compared to the other. Smaller studies tend to be positioned towards the lower end of the publication hierarchy, to have a greater likelihood of being published if they exhibit larger-than-average effects. Consequently, these studies are more prone to satisfying the criterion for statistical significance. It is worth noting that only one study with smaller sample size but was still consistent with the overall trend and which had the largest sample size exhibited larger and varied effect sizes, therefore, the observed phenomenon cannot be attributed to the influence of ‘small study effects’ (See Supplementary J, Figure S26). Hence, the studies incorporated in the analysis seems did not exhibit indications of publication bias. The potential reason for the observed differences between the effect sizes among the larger studies may be attributed to variations in populations or protocols employed in these respective studies (Borenstein, et al., 2022). Moreover, the classic fail-safe N was 8,602; *z*-value of 30.79; *p*-value of < 0.001, suggested that a substantial number of unpublished studies with null results would need to be present in order to diminish the overall pooled effect estimate to a null value. This means that we would need to locate and include 8,602 'null' studies in order for the combined 2-tailed p-value to exceed 0.050. Additionally, the Egger’s test identified that the intercept (B0) is 2.25 (95% CI: 0.99-5.49), *t=*1.42, *df* = 33, p=0.08. Duval and Tweedie’s “fill and trim” method employs a random effects model to identify missing studies (See Supplementary J, Figure S27). Specifically, it focuses on missing studies exclusively to the left side of the mean effect. This method indicates that there are no studies that have been overlooked or omitted. Using Trim and Fill, the values are unchanged from the original overall random-effects OR of 2.64 (95% CI: 2.01–3.46). Moreover, using Begg and Mazumdar rank correlation test suggested that Kendall’s tau *b* is -0.12 (*p*=0.15).

*Publication bias studies of social support and life satisfaction*

Evident from the analysis of the funnel plot, which reveals a predominantly symmetrical distribution. Both smaller and larger sample sizes studies spread on the left and right side of the funnel plot, therefore, it cannot be attributed to the influence of ‘small study effects’ (See Supplementary J, Figure S28). Hence, the studies incorporated in the analysis did not exhibit indications of publication bias. The potential reason for the observed differences between the effect sizes among the larger studies may be attributed to variations in populations or protocols employed in these respective studies (Borenstein, et al., 2022). The classic fail-safe N was 5,116; *z*-value of 31.41; *p*-value of < 0.001, suggested that a substantial number of unpublished studies with null results would need to be present in order to diminish the overall pooled effect estimate to a null value. This means that we would need to locate and include 5,116 'null' studies in order for the combined 2-tailed p-value to exceed 0.050. Additionally, the Egger’s test identified that the intercept (B0) is 1.12 (95% CI: -1.17-3.41), *t=*1.03, *df*=18, *p*=0.16. Duval and Tweedie’s “fill and trim” method employs a random effects model to identify missing studies (See Supplementary J, Figure S29). Specifically, it focuses on missing studies exclusively to the left side of the mean effect. This method indicates that there are no studies that have been overlooked or omitted. Using Trim and Fill, the values are unchanged from the original overall random-effects OR of 3.27 (95% CI: 2.59–4.13). Moreover, using Begg and Mazumdar rank correlation test suggested that Kendall’s tau *b* is 0.05 (*p*=0.39).

*Publication bias studies of loneliness and life satisfaction*

Evident from the analysis of the funnel plot, which reveals a fairly symmetrical distribution in the top plot, However, it is worth to note that there is one study in the bottom of the funnel plot that placed in the right side (See Supplementary J, Figure S30). Therefore, publication of studies investigating the association between loneliness and life satisfaction have possibility to be biased in the direction of showing a stronger association than actually present. The classic fail-safe N was 2,706; z-value of 30.80; p-value of < 0.001, suggested that a substantial number of unpublished studies with null results would need to be present in order to diminish the overall pooled effect estimate to a null value. This means that we would need to locate and include 2,706 'null' studies in order for the combined 2-tailed p-value to exceed 0.050. Additionally, the Egger’s test identified that the intercept (B0) is 3.03 (95% CI: 0.85-5.21), *t=*3.14, *df*=9, *p*=0.006. Duval and Tweedie’s “fill and trim” method employs a random effects model to identify missing studies (See Supplementary J, Figure S31). Specifically, it focuses on missing studies exclusively to the left side of the mean effect. This method indicates that there are two studies that have been overlooked or omitted. The imputed overall pooled random-effects OR that takes publication bias into account was OR of 2.90 (95% CI: 2.25–3.74). This imputed OR is lower but not significantly different to the original overall random-effects OR of 3.30 (95% CI: 2.53–4.30). Moreover, using Begg and Mazumdar rank correlation test suggested that Kendall’s tau *b* is 0.25 (*p*=0.14).

*Publication bias studies of depression and life satisfaction*

Evident from the analysis of the funnel plot, which a fairly asymmetrical distribution in the right plot, however, this plot seems to spread out both at the top and the bottom of the funnel plot (See Supplementary J, Figure S32). Therefore, publication of studies investigating the association between depression and life satisfaction have possibility to be biased in the direction of showing a stronger association than actually present. The classic fail-safe N was 31,777; *z*-value of 71.35; *p*-value of < 0.001, suggested that a substantial number of unpublished studies with null results would need to be present in order to diminish the overall pooled effect estimate to a null value. This means that we would need to locate and include 31,777 'null' studies in order for the combined 2-tailed p-value to exceed 0.050. Additionally, the Egger’s test identified that the intercept (B0) is -1.32 (95% CI: -10.21-7.57), *t=*0.31, *df*=22, *p*=0.38. Duval and Tweedie’s “fill and trim” method (See Supplementary J, Figure S33) employs a random effects model to identify missing studies. Specifically, it focuses on missing studies exclusively to the left side of the mean effect. This method indicates that there are seven studies that have been overlooked or omitted. The imputed overall pooled random-effects OR that takes publication bias into account was OR of 3.46 (95% CI: 2.35–5.09). This imputed OR is lower than the original overall random-effects OR of 4.76 (95% CI: 3.10–7.32). Moreover, using Begg and Mazumdar rank correlation test suggested that Kendall’s tau *b* is 0.09 (*p*=0.19).

*Publication bias studies of anxiety and life satisfaction*

Evident from the analysis of the funnel plot, which reveals a symmetrical distribution (See Supplementary J, Figure S34). Therefore, it cannot be attributed to the influence of ‘small study effects’. Hence, the studies incorporated in the analysis did not exhibit indications of publication bias. The classic fail-safe N was 1,388; *z*-value of 32.71; *p*-value of < 0.001, suggested that a substantial number of unpublished studies with null results would need to be present in order to diminish the overall pooled effect estimate to a null value. This means that we would need to locate and include 1,388 'null' studies in order for the combined 2-tailed p-value to exceed 0.050. Additionally, the Egger’s test identified that the intercept (B0) is 9.05 (95% CI: -4.51-22.61), *t=*2.12, *df*=2, *p*=0.06. Duval and Tweedie’s “fill and trim” method (See Supplementary J, Figure S35) employs a random effects model to identify missing studies. Specifically, it focuses on missing studies exclusively to the left side of the mean effect. This method indicates that there are no studies that have been overlooked or omitted. Using Trim and Fill, the values are unchanged from the original overall random-effects OR of 5.10 (95% CI: 2.21–11.78). Moreover, using Begg and Mazumdar rank correlation test suggested that Kendall’s tau *b* is -0.10 (*p*=0.81).

1. **Physical capability and life satisfaction**

**
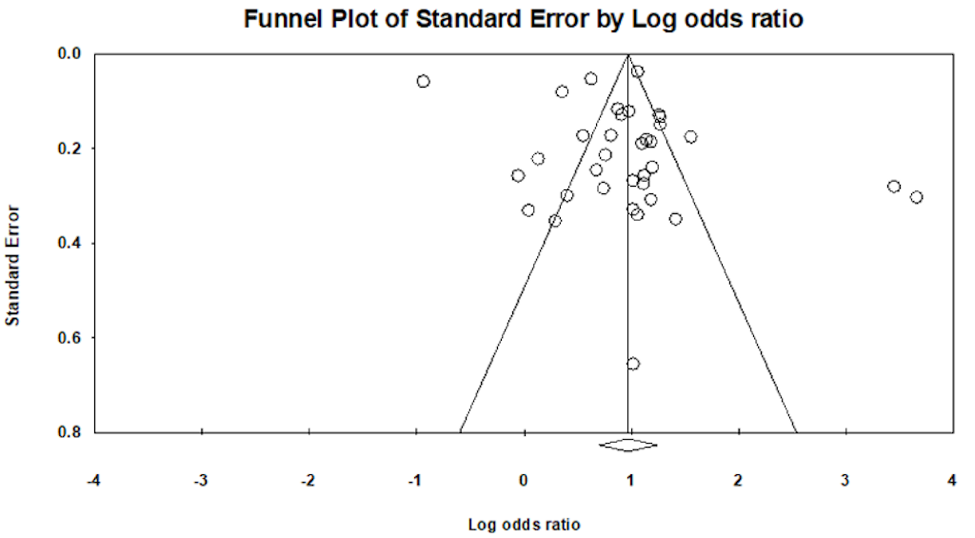
**

Figure S 26. Publication bias Funnel plot for studies of physical capability and life satisfaction

**Duval and Tweedie’s “fill and trim”**


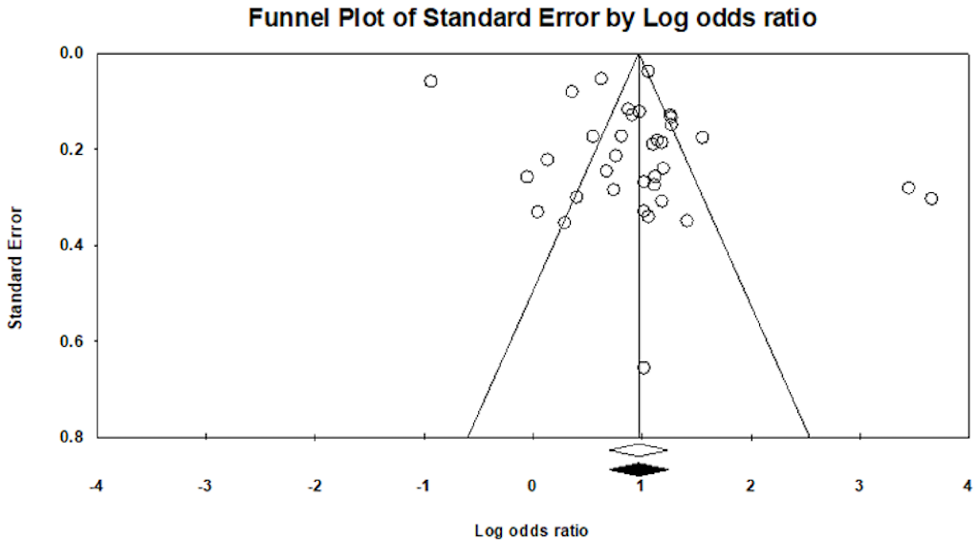


Figure S 27. Duval and Tweedie’s “fill and trim” for studies of physical capability and life satisfaction

1. **Social support and life satisfaction**


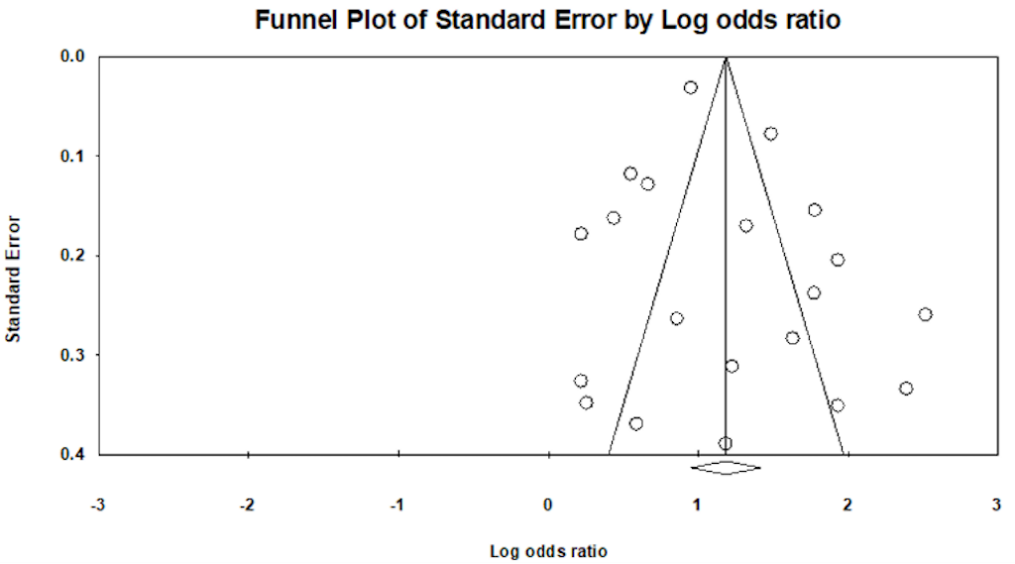


Figure S 28. Publication bias Funnel plot for studies of social support and life satisfaction

**Duval and Tweedie’s “fill and trim”**


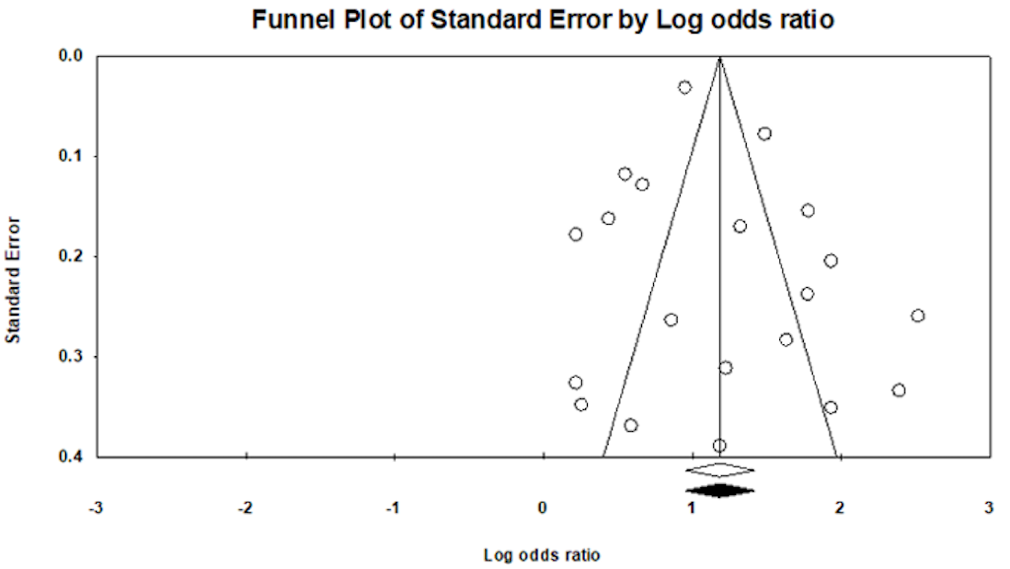


Figure S 29. Duval and Tweedie’s “fill and trim” for studies of social support and life satisfaction

1. **Loneliness and life satisfaction**


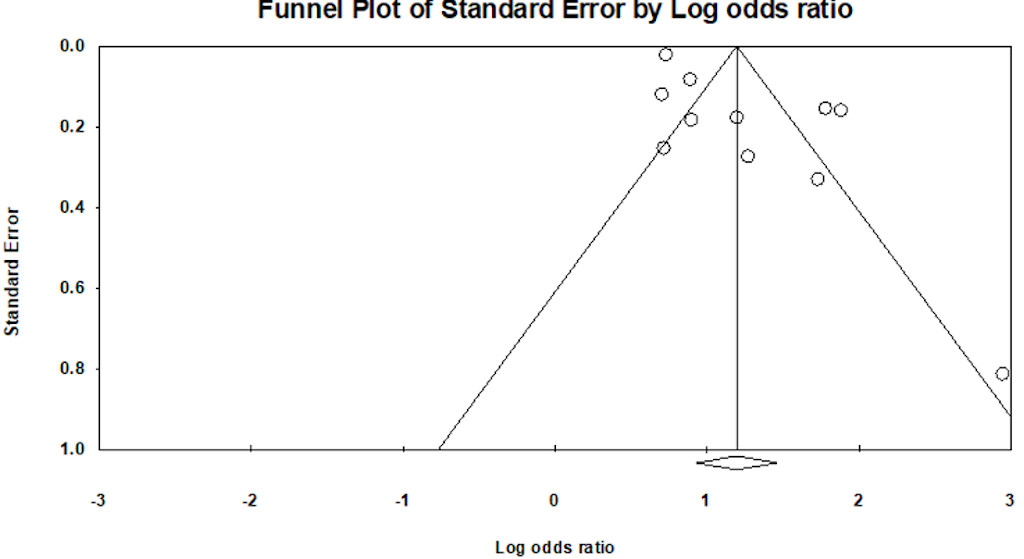


Figure S 30. Publication bias Funnel plot for studies of loneliness and life satisfaction

**Duval and Tweedie’s “fill and trim”**

**
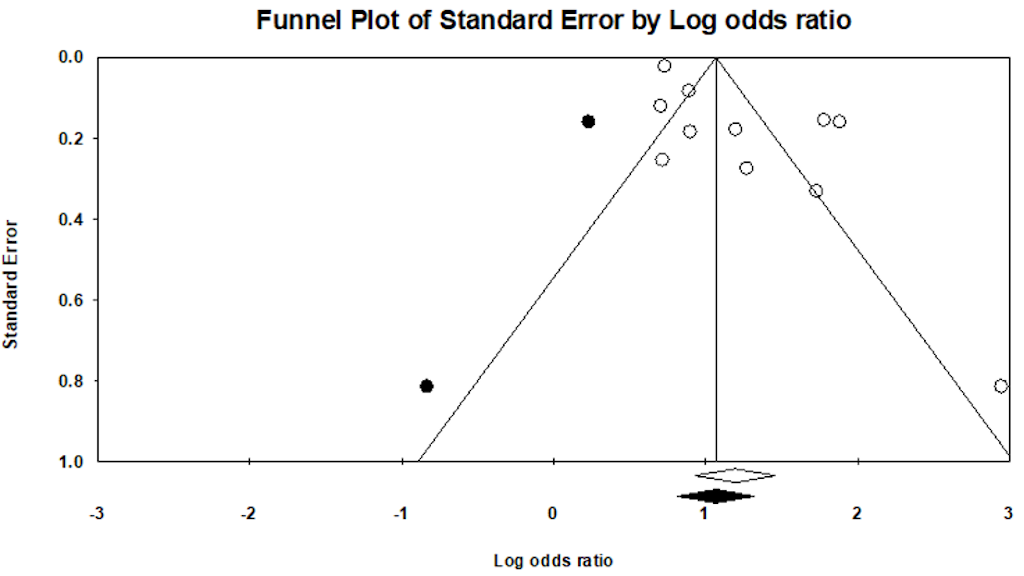
**

Figure S 31. Duval and Tweedie’s “fill and trim” for studies of loneliness and life satisfaction

1. **Depression and life satisfaction**


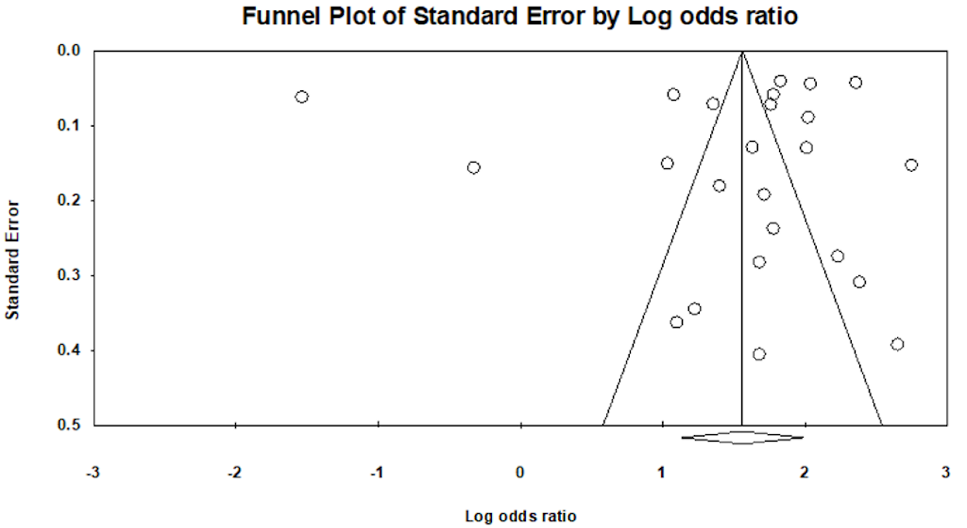


Figure S 32. Publication bias Funnel plot for studies of depression and life satisfaction

**Duval and Tweedie’s “fill and trim”**

**
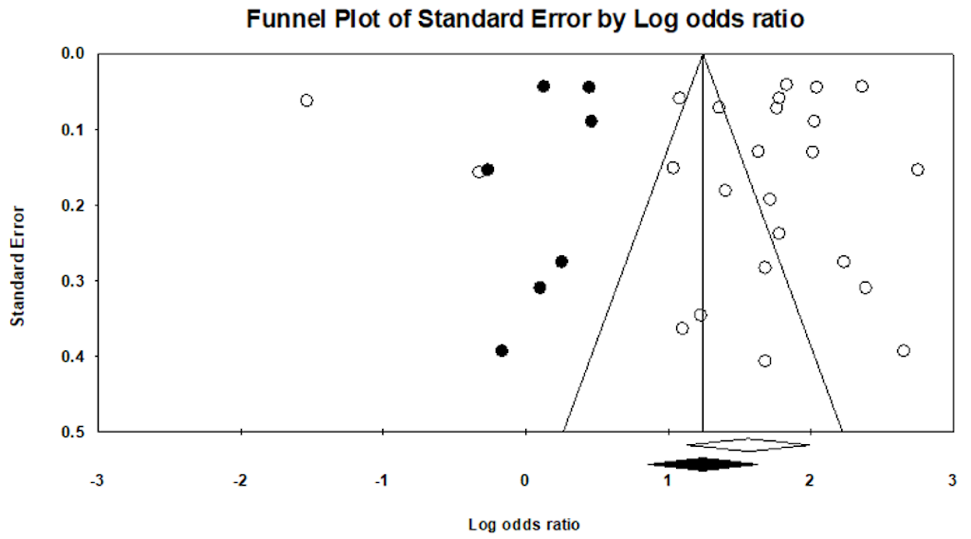
**

Figure S 33. Duval and Tweedie’s “fill and trim” for studies of depression and life satisfaction

1. **Anxiety and life satisfaction**


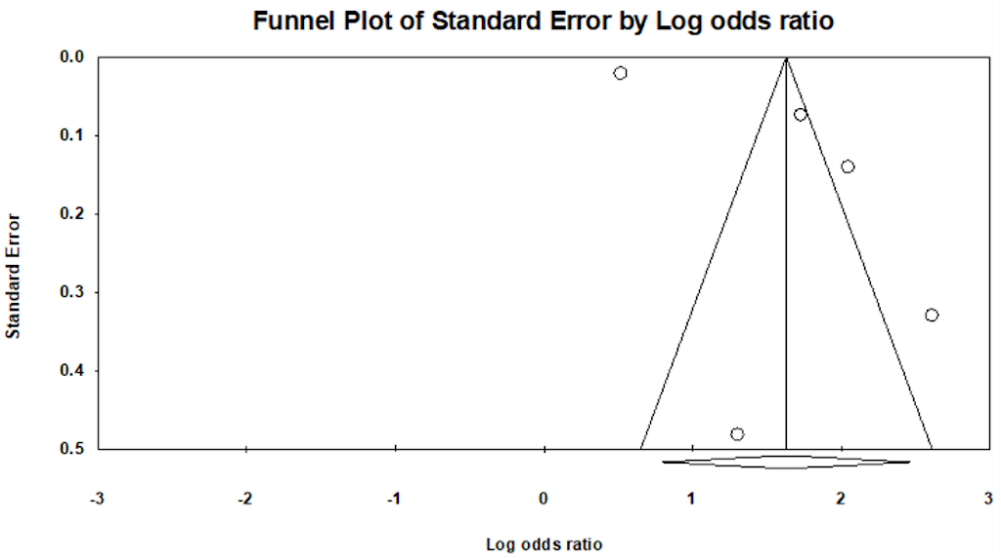


Figure S 34. Publication bias Funnel plot for studies of anxiety and life satisfaction

**Duval and Tweedie’s “fill and trim”**


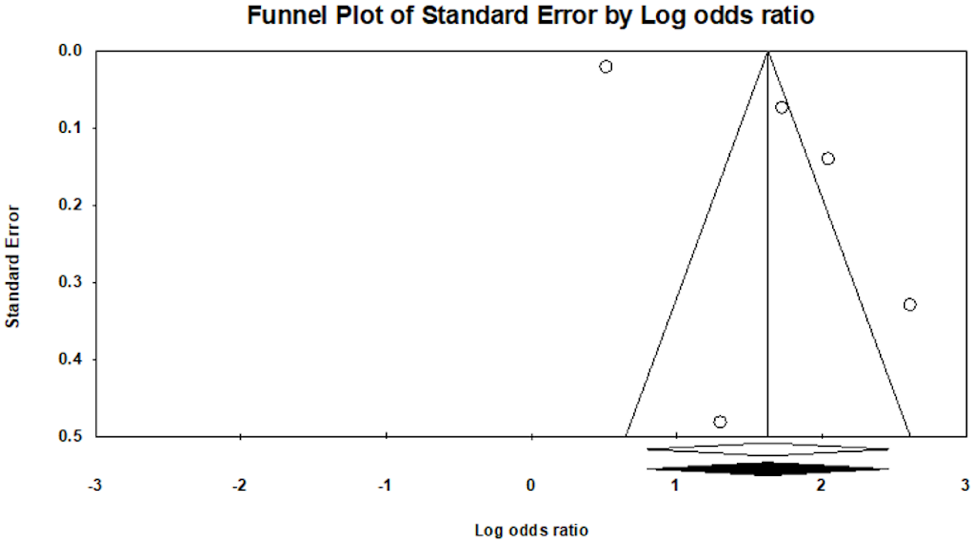


Figure S 35. Duval and Tweedie’s “fill and trim” for studies of anxiety and life satisfaction
